# Brominated B_1_-Polycyclic Aromatic Hydrocarbons for the Synthesis of Deep-Red to Near-Infrared Delayed Fluorescence Emitters

**DOI:** 10.1021/acs.orglett.3c02167

**Published:** 2023-07-27

**Authors:** Kang Yuan, Abhishek Kumar Gupta, Changfeng Si, Marina Uzelac, Eli Zysman-Colman, Michael James Ingleson

**Affiliations:** † EaStCHEM School of Chemistry, The University of Edinburgh, Edinburgh, EH9 3FJ, U.K.; ‡ Organic Semiconductor Centre and EaStCHEM School of Chemistry, University of St Andrews, St Andrews KY16 9ST, U.K.

## Abstract

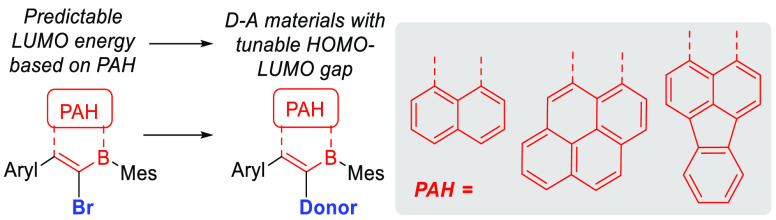

Bromo-functionalized B_1_-polycyclic aromatic hydrocarbons (PAHs) with LUMOs of less than −3.0 eV were synthesized and used in cross-couplings to form donor–acceptor materials. These materials spanned a range of S_1_ energies, with a number showing thermally activated delayed fluorescence and significant emission in the near-infrared region of the spectrum. These B_1_-PAHs represent a useful family of acceptors that can be readily synthesized and functionalized.

The incorporation of boron into polycyclic aromatic hydrocarbons (PAHs) has attracted significant attention as a method for modulating key optoelectronic properties.^1^ Due to the electron deficiency of the boron center in C_3_B units, PAHs containing these moieties (termed B-PAHs) often have stabilized LUMOs (relative to all carbon PAH analogues).^2^ This is exemplified by comparing anthracene (LUMO ≈ −2.4 eV)^3^ to 9,10-diboranthracene [DBA (Figure 1a)], with the latter having a much lower energy LUMO (approximately −3 eV).^4^ Recent breakthroughs have enabled access to B-doped PAHs with deep LUMOs (defined herein as less than −3.0 eV).^5^ This has led to their use in organic electronics applications that require strong acceptors.^6^ However, accessing deep LUMO B-PAHs generally requires the incorporation of multiple boron centers into the PAH core,^5,7^ which increases the synthetic complexity. Furthermore, the use of deep LUMO B-PAHs as strong acceptor units in donor–acceptor (D–A) materials remains underexplored despite the ubiquity of these materials in applications, including in deep-red- and near-infrared-emitting OLEDs.^8^ This paucity is in part due to the shortage of readily accessible deep LUMO B-PAHs that contain a group amenable to functionalization, particularly through cross-coupling reactions.^9^

**Figure 1:**
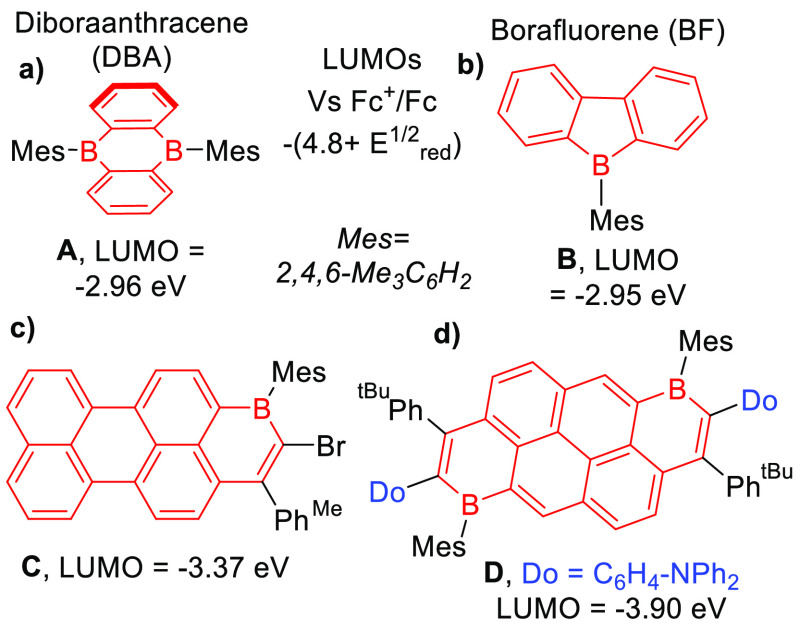
Select previous work on low-LUMO B-PAHs (top) and previous work accessing and using deep LUMO B-PAHs (bottom).

An analysis of reported low-energy LUMO B-PAH-based D–A emissive materials reveals that most are based on DBA or borafluorene (BF) cores (Figure 1b).^10^ While notable D–A materials have been reported using DBA and BF, including red thermally activated delayed fluorescence (TADF) emitters,^11^ the LUMO of these two B-doped PAHs is approximately −3.0 eV (in the absence of electron-withdrawing groups).^12^ This LUMO energy makes it challenging to access D–A materials with emission maxima shifted far into the deep-red and near-infrared (near-IR) region of the spectrum. Furthermore, when DBA and BF are used in D–A materials, the donor unit is often installed as part of the exocyclic group on boron.^10,11^ An alternative approach that we reported recently involves the formation of brominated B-PAHs via the treatment of alkyne-substituted PAHs with BBr_3_.^13^ This one-pot procedure produced bench-stable deep LUMO compounds, such as **C** (Figure 1c). This method was applied to the synthesis of several brominated B-PAHs, with the vinyl bromide unit being attractive for use in cross-coupling reactions. However, in our initial work, only one D–A material was synthesized, and while this compound [**D** (Figure 1d)] had a small HOMO–LUMO gap, it could be synthesized in only poor overall yield (12% from the alkyne) and it did not display delayed fluorescence. The latter was attributed from calculations to an overly large singlet–triplet excited state gap, Δ*E*_ST_. The low yield of compounds such as **D** complicated attempts to access other D–A materials based on these B_2_-PAHs, precluding structure–property relationship studies.

Herein, we report that B_1_-PAHs made through this methodology are useful for accessing families of D–A materials with tunable HOMO–LUMO gaps and S_1_ energies. The deep S_1_ energies of these materials indicate that this family of acceptors can help meet the call set out in a recent review on near-IR emitters that stated, “the development of new functionalized acceptor units with simple structures is still imperative”.^8^

Our previous work used alkynyl-naphthalene, -pyrene, and -perylene derivatives, which were transformed into B_1_-PAHs using BBr_3_.^13b^ At the outset of this study, three B_1_-PAHs were analyzed by cyclic voltammetry (CV), which revealed that the LUMO energy of the B_1_-PAH correlated to the LUMO energy of the PAH core (red, Figure 2). In each case, calculations indicated that the LUMO is localized across the PAH and the boracycle. If this correlation is general, then a PAH with a LUMO energy comparable to that of perylene should give a B_1_-PAH with a deep LUMO. Indeed, a B_1_-PAH derived from fluoranthene was synthesized and found to possess a LUMO energy comparable to that of the perylene analogue (Figure 2).

**Figure 2:**
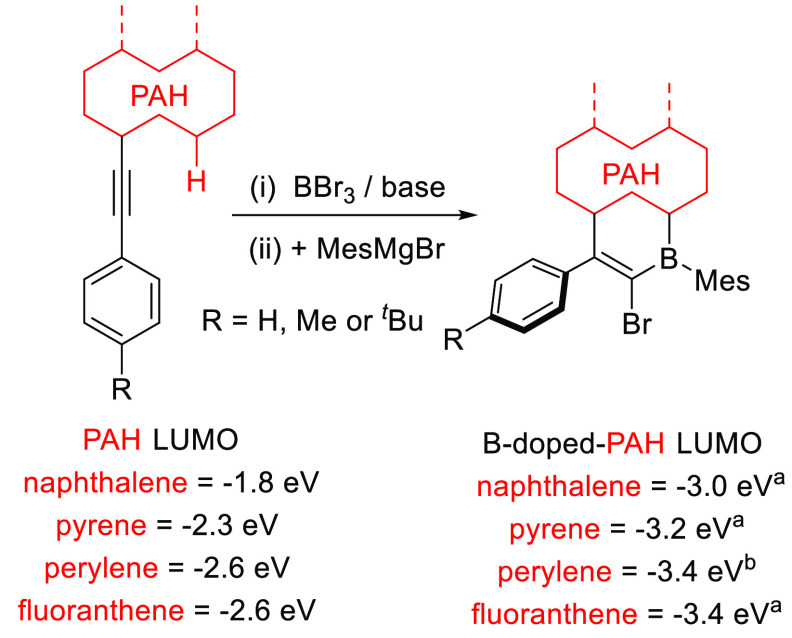
Effect of varying the PAH on the LUMO energy of B_1_-PAH. *E*_LUMO_ = −*E*_1/2_^red^ = 4.8 eV. ^a^This work and ^b^ref (13b).

Three B_1_-PAHs with different LUMO energies were studied further. To assess the effect bromide has on the LUMO energy, a phenyl was installed to form compounds **1–3** (Figure 3) via Negishi cross-coupling reactions. This had a minimal effect on the LUMO energy, with **1–3** being harder to reduce than the bromo derivatives by only 0.05–0.1 V (Figures S43–S50 and Table S1). In each case, the calculated LUMO remained localized over the boracycle and PAH units (Figure S40). This indicated that low-HOMO–LUMO gap/deep S_1_ energy materials would be accessible by installing donor units at this position. Therefore, a number of D–A materials were modeled to guide synthetic studies. Triphenylamine (TPA) was selected on the basis of calculations (Figure S38), which revealed that **4–6** (Figure 3) span a range of S_1_ energies, with all having an appreciable oscillator strength (*f*) for the S_0_–S_1_ transition. Furthermore, small (≤0.25 eV) Δ*E*_ST_ values were calculated, and thus, TADF behavior was expected. The calculated small Δ*E*_ST_ is consistent with both S_1_ and T_1_ states for **4–6** possessing significant charge transfer (CT) character (Figures S39 and S41). Calculations were also performed on **7** (Figure 3), which contains a *p*-phenoxazine-phenyl (PXZ-Ph) donor. While **7** had a calculated S_1_ energy similar to that of **6**, the *f* value associated with the CT-dominated S_0_–S_1_ transition was negligible, attributed to the strongly twisted conformation. Analysis of **4–6** revealed that the HOMO is distributed on both the donor and the boracycle (Figures S38 and S41); thus, there is appreciable spatial overlap with the LUMO, essential for an appreciable *f*. However, for **7**, the HOMO is localized on the PXZ unit, dramatically reducing the extent of spatial overlap with the LUMO (Figure S42). Thus, while the calculated Δ*E*_ST_ was very small for **7**, it was expected to be a very weak near-IR emitter. Finally, a PXZ-Ph fluoranthene derivative, **8**, was calculated. Analogous to **7**, **8** has a CT-dominated S_0_–S_1_ transition with a very low *f*. Nevertheless, the calculated small HOMO–LUMO gap of **8** indicates it may have potential in non-emissive applications.^14^

**Figure 3:**
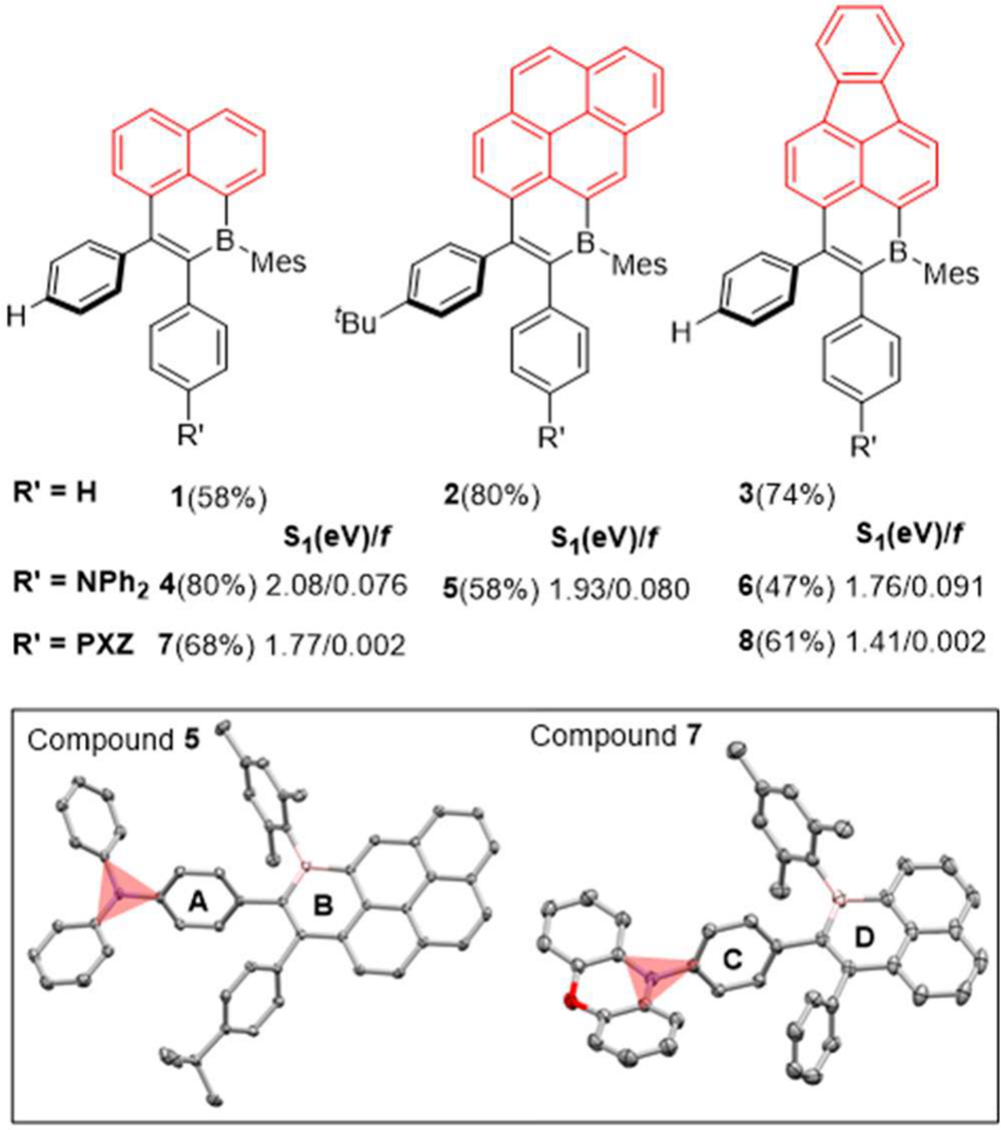
Compounds **1–8**. Yields (in parentheses) are for the Negishi coupling, with calculated S_1_ energy and *f* values for **4–8** (top). Solid state structures of **5** and **7** (bottom), where the ellipsoids are drawn at the 50% probability level with hydrogens omitted.

Compounds **4–8** were synthesized using the same Negishi cross-coupling conditions in respectable overall yields (35–45% from the alkyne). The twisted D–A nature of **4–8** suggested by the calculations was supported by single-crystal X-ray diffraction studies of **5** and **7** (Figure 3, bottom). This revealed that the B_1_-PAH and the phenyl-N ring are twisted, with the angle between the planes of rings A and B in **5** being 58.1° and that between rings C and D in **7** being 61.5°. Both the boron and nitrogen centers are effectively planar (Σ_C–N–C_ values of 357.5° and 355.4° for **5** and **7**, respectively). Another informative metric is the angle between the C_3_N plane (red triangle in Figure 3) and ring A/C, which is 53.1° and 78.9° for **5** and **7**, respectively. This suggests less effective orbital mixing between the NAr_2_ unit and the bridging phenyl for PXZ in **7** relative to NPh_2_ in **5**, consistent with the lower *f* for the S_0_–S_1_ HOMO–LUMO-dominated transition for the PXZ derivatives.

The frontier orbital energies for **4–8** were probed by CV, with all displaying a reversible first reduction wave (Figures S51–S57). The LUMO energy is deepest for the fluoranthene B_1_-PAH derivatives (Table 1), with the LUMO energy effectively identical across the Ph-, TPA-, and PXZ-Ph-substituted derivatives (e.g., **3** vs **6** vs **8**). This is consistent with the LUMO being localized on B_1_-PAH in each case. Notably, the LUMOs are deeper for the B_1_-PAHs containing pyrene and fluoranthene (e.g., **5** and **6**) than those of DBA and BF (with B-Mes substituents). The donor dominates the first oxidation process for **4–8**, with the PXZ-Ph derivatives having a ∼0.15 eV destabilized HOMO energy relative to the TPA congeners.

**Table 1:** Optoelectronic Properties of **3–8**

	*E*_1/2_^red^ (V)^a^	*E*_1/2_^ox^ (V)^a^	LUMO^exp^ (eV)^b^	optical gap (eV)^c^	λ_PL_^Tol^ (nm)^d^	Φ_PL_^Tol^ (%)^e^	λ_PL_^CBP^ (nm)^f^	Φ_PL_ (%)^g^	S_1_/T_1_ (eV)^h^	τ (ns)^f^	τ (μs)^e^
**3**	–1.46	–	–3.34	2.09	501	5 (5)	495	25.4 (25.2)	2.82/2.43	5.7	–
**4**	–1.84	0.50	–2.96	2.23	630	42 (36)	623	39.0 (38.5)	2.26/2.23	80	2.4
**5**	–1.69	0.50	–3.11	1.94	700	3 (3)	682	6.2 (5.8)	2.10/2.08	2.8	1.1
**6**	–1.48	0.50	–3.32	1.87	745	4 (4)	738	2.8 (2.1)	1.95/1.89	2.1	1.0
**7**	–1.79	0.34	–3.01	2.41	660	9 (5)	614	31.7 (23.6)	2.29/2.12	52	1.9
**8**	–1.45	0.34	–3.35	2.06	780	4 (4)	673	4.2 (4.0)	2.09/2.01	–	–

a At 298 K in THF with 0.1 M [^*n*^Bu_4_N]PF_6_ (vs Fc^+^/Fc).

b *E*_LUMO_ = −*E*_1/2_^red^ – 4.8 eV.

c From the onset of the absorption in toluene.

d At 298 K, in degassed toluene at 1 × 10^–5^ M.

e At 298 K, in degassed toluene with values in parentheses under aerated conditions.

f In spin-coated 5 wt % doped films in CBP at 298 K.

g Spin-coated films of 5 wt % emitters doped in CBP under N_2_ with the values in parentheses in air.

h Obtained from the onset of the steady state photoluminescence and phosphorescence spectra (1–9 ms) at 77 K of 5 wt % films in CBP.

With regard to the solution state photophysical properties, weak broad absorption bands are observed in **4–6** (Figure 4a), which are assigned to CT transitions based on calculations (Figure S39). There is the expected shift to lower energies of the CT band across these three compounds, which is aligned with the increasing acceptor strength of the B_1_-PAH. Compared to **4**–**6**, there are no obvious CT absorption bands for **7** and **8** consistent with the negligible *f* calculated for the S_0_–S_1_ transitions in these two compounds. All D–A compounds exhibit unstructured and broad photoluminescence (PL) spectra in toluene (Figure 4a), indicative of an excited state with strong CT character. For the TPA series, the emission bands are red-shifted with maxima (λ_PL_) at 630, 700, and 745 nm for **4**–**6**, respectively, consistent with the progressively stabilized computed S_1_ energies. As expected, the use of the stronger donor PXZ in **7** and **8** produces a more red-shifted emission (λ_PL_ at 660 and 780 nm) in toluene compared to TPA analogues **4** and **6**, respectively. The PL spectra of each of the D–A compounds exhibit strong positive solvatochromism (Figure S58) and become broader with an increase in solvent polarity, consistent with the CT nature of the emissive excited state.

**Figure 4:**
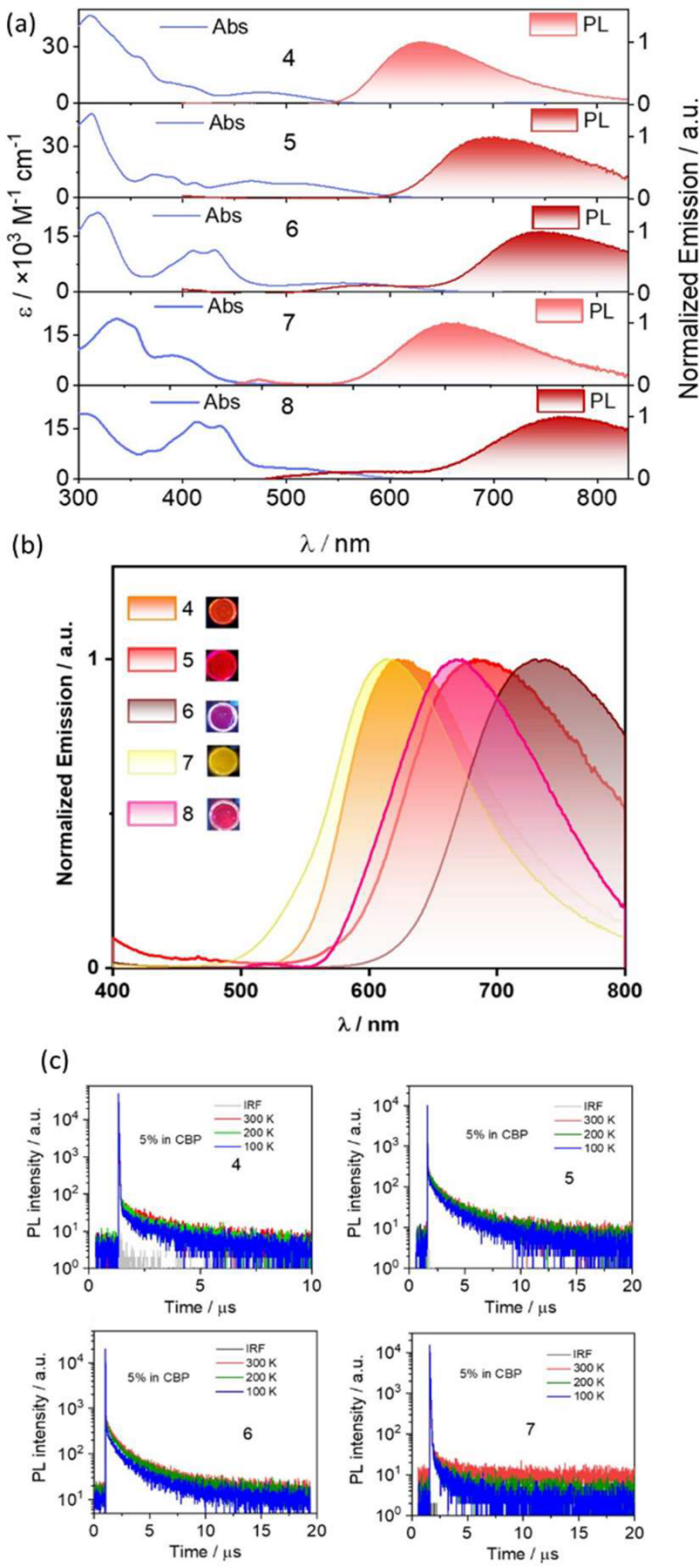
(a) UV–vis absorption and PL spectra of **4**–**8** in toluene (λ_exc_ = 330 nm). (b) Emission spectra of 5 wt % doped films of **4–8** in CBP (λ_exc_ = 330 nm). (c) Time-correlated single-photon counting plots for **4–7** (λ_exc_ = 375 nm).

Next, the photoluminescence behavior of **4**–**8** was measured as thin films in PMMA (Figure S60). **4–6** exhibited λ_PL_ values similar to those in toluene; however, **7** and **8** showed blue-shifted emission compared to those in toluene (λ_PL_ at 580 and 670 nm, respectively). This is tentatively assigned to PXZ adopting a different conformation in PMMA, which has been previously observed.^15^ We then investigated the photophysical behavior of the emitters in 4,4′-bis(*N*-carbazolyl)-1,1′-biphenyl (CBP) that has a high triplet energy (2.55 eV).^16^ Compounds **4–6** emit at 623, 682, and 738 nm (Figure 4b), respectively, with Φ_PL_ values of 39.0%, 6.2%, and 2.8%, respectively (Table 1). The time-resolved PL of **4–6** reveals (Figure 4c) biexponential decay kinetics consisting of a nanosecond prompt component and a microsecond delayed component (Table 1). The delayed emission tended to increase with temperature, which is consistent with TADF for these three compounds. With regard to the PXZ derivatives, the λ_PL_ again is significantly blue-shifted compared to the emission of **7** and **8** in toluene (Table 1); this is tentatively assigned to PXZ adopting a different conformation in CBP films.^15^ The TADF nature of the emission from **7** was confirmed by the temperature-dependent nature of the delayed emission (Figure 4c). Delayed emission was not detected for **8** in CBP. Finally, the S_1_/T_1_ energies were probed at 77 K for **4–8 (**Figure S66), with the S_1_/T_1_ states being close in energy, consistent with the observed TADF (Table 1).

In summary, this work introduces a series of bench-stable bromo-functionalized B_1_-PAHs that have LUMOs ranging between −3.0 and −3.4 eV. They can be used to form D–A materials all under the same Negishi coupling conditions in moderate yields. A number of the D–A materials displayed TADF with significant emission in the near-IR region of the spectrum. This work demonstrates the promise of using these readily accessible boron-doped arenes as strong acceptors to produce NIR-emitting TADF compounds.

## Data Availability

The data underlying this study are available in the published article and its Supporting Information.

## Supporting Information Available

Full experimental details, NMR spectra for all new compounds, crystal structures, and plots of optoelectronic data (PDF)
Cartesian coordinates (XYZ)
Cartesian coordinates (XYZ)
Cartesian coordinates (XYZ)
Cartesian coordinates (XYZ)
Cartesian coordinates (XYZ)
Cartesian coordinates (XYZ)
Cartesian coordinates (XYZ)
Cartesian coordinates (XYZ)

## Supplementary Material

ol3c02167_si_001.pdf

ol3c02167_si_002.xyz

ol3c02167_si_003.xyz

ol3c02167_si_004.xyz

ol3c02167_si_005.xyz

ol3c02167_si_006.xyz

ol3c02167_si_007.xyz

ol3c02167_si_008.xyz

ol3c02167_si_009.xyz
