# Correction to “Brominated B_1_-Polycyclic Aromatic Hydrocarbons for the Synthesis of Deep-Red to Near-Infrared Delayed Fluorescence Emitters”

**DOI:** 10.1021/acs.orglett.3c02942

**Published:** 2023-09-19

**Authors:** Kang Yuan, Abhishek Kumar Gupta, Changfeng Si, Marina Uzelac, Eli Zysman-Colman, Michael James Ingleson

A Data Availability Statement is added to include the data underpinning the calculations and photophysics described in this paper.

## Data Availability

The research data supporting this publication can be accessed at 10.17630/fd1c4863-0606-4595-9c89-f40ef45fef4d.

