# Supplementary material for: Brominated B1-Polycyclic Aromatic Hydrocarbons for the Synthesis of Deep-Red to Near-Infrared Delayed Fluorescence Emitters
Source: Org Lett. 2023 Jul 27;25(31):5880–4. doi: 10.1021/acs.orglett.3c02167 (PMC10425980; doi:10.1021/acs.orglett.3c02167)
Supplement: Supplementary file 1 — ol3c02167_si_001.pdf [file ol3c02167_si_001.pdf]

Supporting information

Brominated B_1-Polycyclic Aromatic Hydrocarbons for the Synthesis of Deep-Red to Near-IR Delayed Fluorescence Emitters.

Kang Yuan[a], Abhishek Kumar Gupta[b], Changfeng Si[b], Marina Uzelac[a], Eli Zysman-Colman[b,*], Michael James Ingleson[a,*]

a EaStCHEM School of Chemistry, The University of Edinburgh, Edinburgh, EH9 3FJ

b Organic Semiconductor Centre and EaStCHEM School of Chemistry, University of St Andrews, KY16 9ST

Table of Contents

S1. General Description

S2. Synthetic Procedures

S3. NMR Spectra

S4. Crystal Structures

S5. DFT Calculations

S6. Electrochemical Properties

S7. Photophysical Properties

S8. References

S1. General description

All experiments were performed under a nitrogen atmosphere in oven-dried glassware. Solvents were obtained from an Inert PureSolv MD5 SPS. All solvents were stored over 3 Å molecular sieves. All chemicals were purchased from commercial sources and used as received. Column chromatography was performed using a CombiFlash NextGen 300+ AutoColumn or manually (40-63 μm silica). Solution ^{1}H, $^{13}C\{^{1}H\}$ and ^{11}B NMR spectra were recorded on 400 MHz and 500 MHz Bruker Spectrometers. ^{1}H and ^{13}C chemical shifts were referenced to residual solvent signals. ^{11}B chemical shift was referenced to external BF_3□OEt_2. High resolution mass spectrometry was performed at the Resource Centre for Advanced Mass Spectrometry based in the School of Chemistry at the University of Edinburgh. High resolution mass spectra were recorded on a VG autospec, or Thermo/Finnigan MAT 900, mass spectrometer. Electron Impact (EI+) spectra were performed at 70 eV using methane as the carrier gas, with either a double focusing sector field (DFSF) or time-of-flight (TOF) mass analyzer. Chemical Ionization (CI+) spectra were performed with methane reagent gas, with either a double focusing sector field (DFSF) or time-of-flight (TOF) mass analyzer. Electrospray Ionization (ESI) spectra were performed using a time-of-flight (TOF) mass analyzer. Data are reported in the form of m/z. All exact masses were calculated using the predominant isotopes, which (for the heteroatoms) are: ^{11}B and ^{79}Br.

Electrochemistry

Cyclic voltammetry (CV) measurements were performed under a N_2 atmosphere using a CH-Instrument 1110C Electrochemical/Analyzer potentiostat. Experiments were conducted using a 1 mM analyte solution with 0.1 M tetra-*n*-butylammonium hexafluorophosphate as the supporting electrolyte in THF with a scan rate of 100 mV s^{-1}. A glassy carbon electrode with a circular surface (diameter 3 mm) was used as the working electrode with platinum wires as the counter electrode and the reference electrode. The working electrode was polished with alumina slurry, then washed with deionised water and acetone and dried under a stream of pure nitrogen. All potentials were calibrated against the ferrocene/ferrocenium (Fc/Fc^{+}) redox couple.

Photophysical Measurements

All samples were prepared in HPLC grade hexane, toluene (PhMe), diethyl ether (Et_2O) and dichloromethane (DCM) with varying concentrations on the order of 10^{-5} or 10^{-6} M for

absorption and emission studies. Absorption spectra were recorded at RT using a Shimadzu UV-2600 double beam spectrophotometer. Molar absorptivity determination was verified by linear least-squares fit of values obtained from at least five independent solutions at varying concentrations with absorbance ranging from 1.7×10^{-5} to 6.9×10^{-5} M^{-1} cm^{-1} for **1**, 1.5×10^{-5} to 6.2×10^{-5} M^{-1} cm^{-1} for **2**, 2.2×10^{-5} to 9.0×10^{-5} M^{-1} cm^{-1} for **3**, 1.2×10^{-5} to 4.5×10^{-5} M^{-1} cm^{-1} for **4**, 1.2×10^{-5} to 6.8×10^{-5} M^{-1} cm^{-1} for **5**, 9.3×10^{-6} to 3.7×10^{-5} M^{-1} cm^{-1} for **6**, 1.8×10^{-6} to 6.8×10^{-5} M^{-1} cm^{-1} for **7**, 5.8×10^{-6} to 2.9×10^{-5} M^{-1} cm^{-1} for **8**.

Steady-state emission and time-resolved PL decay

Steady-state emission and time-resolved emission spectra were recorded at 298 K using Edinburgh Instruments F980 and FS5 fluorimeters. All the samples for the steady-state measurements were excited at 330 nm using a Xenon lamp, while the samples for the time-resolved measurements were excited at 378 nm using a pico-second laser (PicoQuant, LDH-D-C-375) driven by a laser driver (PDL 800-D). The short-time range (500 ns – 20 μs) of PL decays were measured using time-correlated single photon counting (TCSPC) mode.

Photoluminescence quantum yields (PLQY) in solid thin film

A Hamamatsu C9920-02 integrating sphere was employed for Φ_{PL} measurements for thin film samples. A xenon lamp coupled to a monochromator enabled selective excitation, chosen here to be 305 nm. The output was then fed into the integrating sphere via a fibre, exciting the sample. PL spectra were collected with a multimode fibre and detected with a back-thinned CCD. Doped thin films were prepared by mixing 10 wt% sample and PMMA or varying doping concentrations of emitter and CBP in chloroform solution, followed by spin-coating on a quartz substrate. The quantum yields of the films were measured in air and N_2 atmosphere by purging the integrating sphere with flowing N_2 gas.

ΔE_{ST} measurement

The singlet-triplet splitting energy, ΔE_{ST}, was estimated by recording the steady state emission (SSE) and the phosphorescence spectra at 77 K. The films for ΔE_{ST} measurements were prepared by spin-coating of a 5 wt% of **4-8** and 2 wt% of **1-3** in 4,4′-bis(*N*-carbazolyl)-1,1′-biphenyl, (CBP) chloroform solution onto a cleaned sapphire substrates. All samples were loaded inside a cold finger cryostat (Oxford Instruments) and samples placed under vacuum, with the temperature controlled from 300 K – 77 K. For steady-state emission, all samples were

photoexcited using a xenon lamp at 340 nm. Phosphorescence spectra of thin films were measured using the time-gated method, where the samples were excited at 340 nm by a 5 W xenon microsecond flash lamp in the FS5 fluorimeter. The energy values of the lowest singlet and triplet states were determined from the onsets of steady-state photoluminescence and phosphorescence spectra at 77 K, respectively.

Fitting of the time-resolved luminescence measurements:

Time-resolved PL measurements were fitted to a sum of exponentials decay model, with chi-squared (χ^2) values of between 1 and 2, using the EI FS5 software. Each component of the decay is assigned a weight, (w_i), which is the contribution of the emission from each component to the total emission.

The average lifetime was then calculated using the following:

- Two exponential decay model:

$$\tau_{AVG} = \tau_1 w_1 + \tau_2 w_2$$

with weights defined as $w_1 = \frac{A1\tau_1}{A1\tau_1 + A2\tau_2}$ and $w_2 = \frac{A2\tau_2}{A1\tau_1 + A2\tau_2}$ where A1 and A2 are the preexponential-factors of each component.

- Three exponential decay model:

$$\tau_{AVG} = \tau_1 w_1 + \tau_2 w_2 + \tau_3 w_3$$

with weights defined as $w_1 = \frac{A1\tau_1}{A1\tau_1 + A2\tau_2 + A3\tau_3}$, $w_2 = \frac{A2\tau_2}{A1\tau_1 + A2\tau_2 + A3\tau_3}$ and $w_3 = \frac{A3\tau_3}{A1\tau_1 + A2\tau_2 + A3\tau_3}$ where A1, A2 and A3 are the preexponential-factors of each component.

Theoretical Calculation

Density functional theoretical (DFT) calculations and time-dependent density functional theoretical (TDDFT) calculations were performed using Gaussian 09 Revision D.01 software[1] in the gas phase. The ground state geometries were optimized employing the PBE0[2] functional with the Pople 6-31G(d,p) basis set,[3] in the gas phase. Transitions to excited singlet states and

triplet states were calculated using TDDFT within the Tamm-Dancoff approximation (TDA) based on the optimized ground-state geometries.[4]

S2. Synthetic procedures

Synthesis of BPAH1

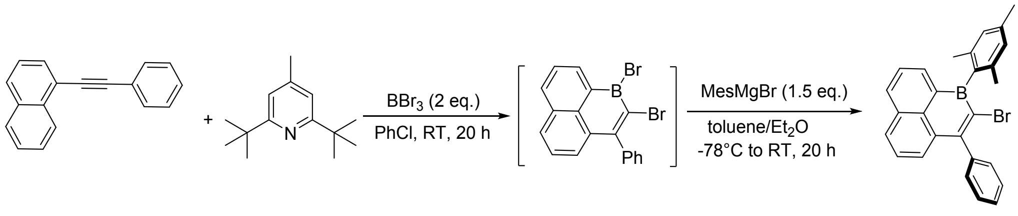

To a solution of 1-(2-phenylethynyl)naphthalene (456 mg, 2 mmol) and 2,6-di-*tert*-butyl-4-methylpyridine (410 mg, 2 mmol) in chlorobenzene (6 mL) was added boron tribromide (4 mL, 1 M in heptane) dropwise. The obtained solution was stirred at room temperature for 20 hours to give a deep yellow suspension. The suspension was filtered under an inert atmosphere. The solid residue was washed with toluene (3 mL) and the filtration was repeated until a white solid (pyridinium salt) was left. All volatiles were removed from the filtrate in *vacuo* and then the oily mixture was dissolved in 6 mL toluene. MesMgBr (3 mL, 1 M in Et_2O) was added to the solution dropwise at -78 °C. The resulting mixture was allowed to gradually warm to room temperature and stirred overnight. The reaction was then quenched with saturated aqueous NH_4Cl and extracted with ethyl acetate. The organic layer was collected and dried over Na_2SO_4. The pure product (490 mg, 1.12 mmol, 56%) was obtained as a yellow solid after column chromatography (silica, dichloromethane: petroleum ether 1:100 to 1:10).

^{1}H NMR (500 MHz, $CDCl_3$) δ = 8.32 (dd, *J* = 8.1, 1.5 Hz, 1H), 8.23 (dd, *J* = 7.0, 1.5 Hz, 1H), 8.11 (dd, *J* = 8.0, 1.4 Hz, 1H), 7.71 (dd, *J* = 8.2, 6.9 Hz, 1H), 7.64 – 7.58 (m, 1H), 7.56 – 7.48 (m, 4H), 7.43 – 7.37 (m, 2H), 6.93 (s, 2H), 2.38 (s, 3H), 2.12 (s, 6H). **^{11}B NMR (160 MHz, $CDCl_3$)** δ = 58.5. **^{13}C{^{1}H} NMR (126 MHz, $CDCl_3$)** δ = 159.7, 144.8, 141.7, 138.4, 137.2, 137.2, 136.5(br, B-***C***), 135.0, 134.7 (br, B-***C***), 134.2, 132.8, 132.5 (br, B-***C***), 132.1, 129.7, 129.4, 129.1, 128.2, 127.9, 127.0, 126.2, 23.2, 21.6. **HRMS (EI)** *m/z* calcd for $C_{27}H_{22}BBr$ $[M]^+$ 436.0992, found 436.0991.

Synthesis of BPAH2

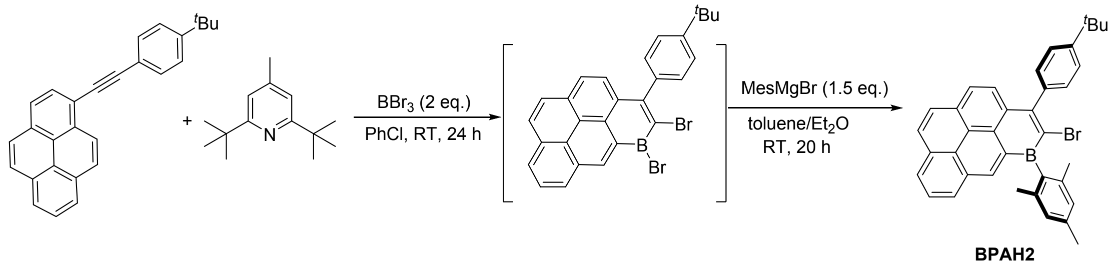

BPAH2

To a solution of 1-((4-(*tert*-butyl)phenyl)ethynyl)pyrene (816 mg, 2.28 mmol) and 2,6-di-*tert*-butyl-4-methylpyridine (467 mg, 2.28 mmol) in chlorobenzene (8 mL) was added boron tribromide (5 mL, 1 M in heptane) dropwise. The obtained solution was stirred at room temperature for 24 hours to give a dark red suspension. The suspension was filtered under an inert atmosphere. The solid residue was washed with toluene (10 mL) and the filtration repeated until a white solid (pyridinium salt) was left. Then the volume of the filtrate was reduced by half. To the mixture was added MesMgBr (3.42 mL, 1 M in Et_2O) dropwise at room temperature. After being stirred at room temperature overnight, the reaction was then quenched with saturated aqueous NH_4Cl and extracted with ethyl acetate. The organic layer was collected and dried over Na_2SO_4. The pure product **BPAH2** (1 g, 1.76 mmol, 79%) was obtained after column chromatography (silica, dichloromethane: petroleum ether 1:100 to 1:10).

^{1}H NMR (500 MHz, $CDCl_3$) δ = 9.06 (s, 1H), 8.49 (d, *J* = 7.7 Hz, 2H), 8.22 – 8.11 (m, 4H), 7.98 (d, *J* = 8.3 Hz, 1H), 7.60 (d, *J* = 8.3 Hz, 2H), 7.40 (d, *J* = 8.3 Hz, 2H), 7.01 (s, 2H), 2.45 (s, 3H), 2.21 (s, 6H), 1.47 (s, 9H). **^{11}B NMR (160 MHz, $CDCl_3$)** δ = 58.4. **^{13}C{^{1}H} NMR (126 MHz, $CDCl_3$)** δ = 161.6, 150.7, 149.8, 141.2 (br, B-***C***), 139.2, 138.8, 137.2, 135.9 (br, B-***C***), 134.7 (br, B-***C***), 133.2, 131.4, 131.3, 131.2, 131.1, 130.9, 130.0, 128.9, 128.4, 128.1, 127.4, 127.1, 126.7, 125.8, 125.2, 124.8, 123.8, 34.9, 31.7, 23.4, 21.6. **HRMS (LDI)** *m/z* calcd for $C_{37}H_{32}BBr$ $[M]^+$ 566.1780, found 566.1789.

Synthesis of BPAH3

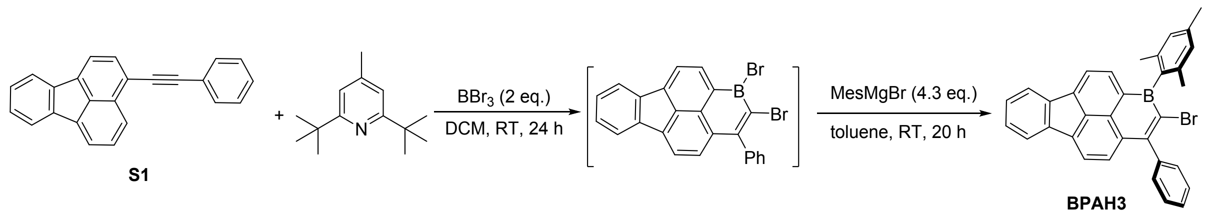

To a solution of 3-(2-phenylethynyl)fluoranthene (565 mg, 1.87 mmol) and 2,6-di-*tert*-butyl-4-methylpyridine (383 mg, 1.87 mmol) in 15 mL dichloromethane was added boron tribromide (3.8 mL, 1 M in DCM) dropwise. The obtained solution was stirred at room temperature for 24 hours. All volatiles were removed under vacuum and 10 mL toluene was added to the resultant solids to give a dark red suspension (due to the poor solubility of the intermediate in toluene, attempts to separate the intermediate from the protonated base failed). To the suspension was slowly added 8 mL MesMgBr solution (1 M in Et_2O) and the resulting homogeneous solution was stirred at room temperature for 20 h. The reaction was then quenched with saturated NH_4Cl aqueous solution and extracted with ethyl acetate. The organic layer was collected and dried over Na_2SO_4. The pure product **BPAH3** was obtained as a dark red solid after column chromatography (silica, ethyl acetate: petroleum ether 0 to 1:10). Yield: 380 mg, 74%.

^{1}H NMR (500 MHz, CD_2Cl_2) δ = 7.86 (d, *J* = 7.0 Hz, 1H), 7.67 (d, *J* = 7.0 Hz, 1H), 7.65 – 7.60 (m, 1H), 7.58 – 7.56 (m, 1H), 7.55 – 7.52 (m, 2H), 7.50 – 7.48 (m, 1H), 7.45 (d, *J* = 7.4 Hz, 1H), 7.39 – 7.37 (m, 2H), 7.23 – 7.19 (m, 2H), 7.15 (d, *J* = 7.4 Hz, 1H), 6.90 (s, 2H), 2.35 (s, 3H), 2.16 (s, 6H). **^{11}B NMR (160 MHz, CD_2Cl_2)** δ = 59.1. **^{13}C{^{1}H} (126 MHz, CD_2Cl_2)** δ = 159.5, 146.4, 146.1, 141.8, 141.5, 141.1, 140.8(br, B-***C***), 138.6, 137.7, 137.0 (br, B-***C***), 136.0, 135.9, 135.8 (br, B-***C***), 133.9, 130.3, 129.7, 129.7, 129.5, 128.8, 128.4, 127.4(127.39), 127.4(127.36), 123.7, 123.4, 121.1, 120.9, 23.1, 21.6. **HRMS (EI)** *m/z* calcd for $C_{33}H_{24}BBr$ $[M]^+$ 510.1149, found 510.1164.

General procedure A: Negishi cross-coupling reactions of BPAH with Ph_2Zn

To an ampoule charged with **BPAH** (1 equiv.), $Pd_2(dba)_3$ (0.03 eq.), SPhos (0.11 equiv.) and THF (7 mL per mmol) was added Ph_2Zn (0.7 eq., in THF). The reaction was heated at 70 °C in an oil bath for 20 hours. The reaction was then quenched with saturated NH_4Cl aqueous solution and extracted with ethyl acetate. The organic layer was collected and dried over Na_2SO_4. The pure product was obtained after column chromatography (silica, dichloromethane: petroleum ether 1:100 to 1:10).

Synthesis of compound 1

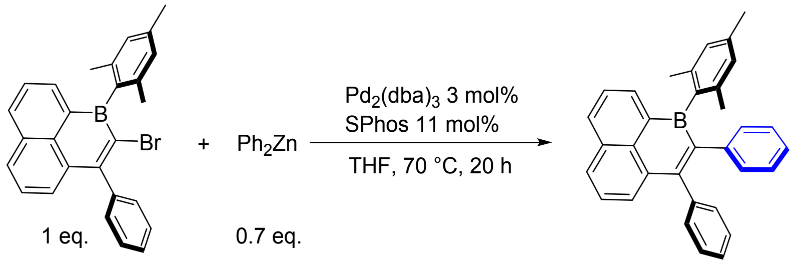

The reaction was performed according to **General procedure A** using **BPAH1** (306 mg, 0.7 mmol), $Pd_2(dba)_3$ (19 mg, 0.021 mmol), SPhos (33 mg, 0.08 mmol), THF (5 mL) and Ph_2Zn (110 mg, 0.5 mmol, in 5 mL THF). The pure product was obtained as a yellow solid after column chromatography (silica, dichloromethane: petroleum ether 1:100 to 1:10). Yield: 177 mg (0.41 mmol, 58%), amorphous yellow solid.

^{1}H NMR (500 MHz, CD_2Cl_2) δ = 8.33 (dd, *J* = 8.1, 1.5 Hz, 1H), 8.11 (dd, *J* = 8.1, 1.3 Hz, 1H), 8.09 (dd, *J* = 6.9, 1.4 Hz, 1H), 7.73 (dd, *J* = 8.0, 6.9 Hz, 1H), 7.67 (dd, *J* = 7.5, 1.2 Hz, 1H), 7.58 (t, *J* = 7.7 Hz, 1H), 7.32 – 7.17 (m, 5H), 7.00 – 6.80 (m, 5H), 6.73 (s, 2H), 2.26 (s, 3H), 2.01 (s, 6H). **^{11}B NMR (160 MHz, CD_2Cl_2)** δ = 59.2. **^{13}C{^{1}H} (126 MHz, CD_2Cl_2)** δ = 157.8, 149.0 (br, B-***C***), 145.2, 143.6, 142.0 (br, B-***C***), 141.1, 138.4, 136.8, 136.7, 135.6 (br, B-***C***), 135.0, 134.7, 132.6, 132.4, 131.4, 130.6, 129.7, 127.7, 127.2 (127.22), 127.2 (127.19), 127.1, 127.0, 126.3, 125.0, 23.7, 21.4. **HRMS (LDI)** *m/z* calcd for $C_{33}H_{27}B$ $[M]^+$ 434.2206, found 434.2203.

Synthesis of compound 2

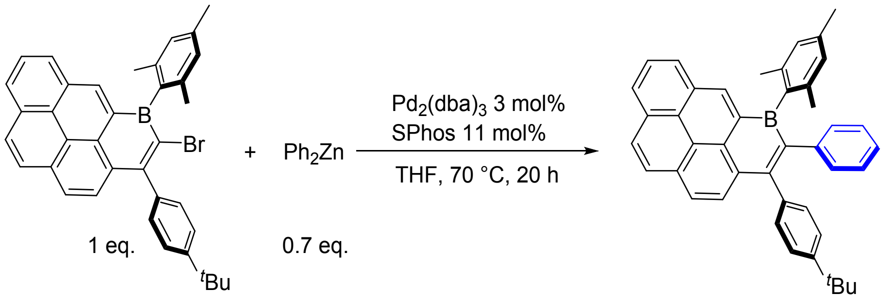

The reaction was performed according to **General procedure A** using **BPAH2** (354 mg, 0.62 mmol), $Pd_2(dba)_3$ (17 mg, 0.019 mmol), SPhos (33 mg, 0.08 mmol), THF (5 mL) and Ph_2Zn (103 mg, 0.47 mmol, in 5 mL THF). The pure product was obtained as a yellow solid after column chromatography (silica, dichloromethane: petroleum ether 1:100 to 1:10). Yield: 279 mg (0.49 mmol, 80%), amorphous red solid.

^{1}H NMR (400 MHz, CD_2Cl_2) δ = 8.92 (s, 1H), 8.48 (ddd, *J* = 12.1, 7.7, 1.1 Hz, 2H), 8.28 – 8.16 (m, 3H), 8.13 (t, *J* = 7.6 Hz, 1H), 8.08 (d, *J* = 8.3 Hz, 1H), 7.33 (d, *J* = 8.4 Hz, 2H), 7.22 (d, *J* = 8.3 Hz, 2H), 7.00 – 6.83 (m, 5H), 6.80 (s, 2H), 2.32 (s, 3H), 2.08 (s, 6H), 1.34 (s, 9H). **^{11}B NMR (128 MHz, CD_2Cl_2)** δ = 61.5. **^{13}C{^{1}H} NMR (126 MHz, CD_2Cl_2)** δ = 159.6, 150.2, 148.6(br, B-***C***), 148.3, 145.5, 142.2 (br, B-***C***), 138.8, 138.5, 136.7, 135.3 (br, B-***C***), 133.3, 132.0, 131.4, 131.4, 131.1, 131.0, 130.6, 129.9, 128.5, 128.4, 128.3, 128.3, 127.1, 127.1, 127.0, 126.2, 124.9, 124.8, 124.7, 123.9, 35.0, 31.7, 23.8, 21.5. **HRMS (LDI)** *m/z* calcd for $C_{43}H_{37}B$ $[M]^+$ 564.2988, found 564.2955.

Synthesis of compound 3

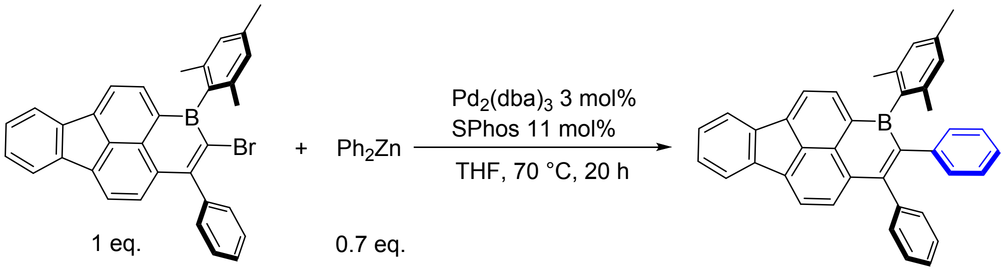

The reaction was performed according to **General procedure A** using **BPAH3** (307 mg, 0.6 mmol), $Pd_2(dba)_3$ (17 mg, 0.019 mmol), SPhos (30 mg, 0.072 mmol), THF (5 mL) and Ph_2Zn (99 mg, 0.45 mmol, in 5 mL THF). The pure product was obtained as a yellow solid after column chromatography (silica, dichloromethane: petroleum ether 1:100 to 1:10). Yield: 227 mg (0.45 mmol, 74%), amorphous red solid.

^{1}H NMR (500 MHz, CD_2Cl_2) δ = 7.78 (d, *J* = 6.9 Hz, 1H), 7.70 (d, *J* = 6.9 Hz, 1H), 7.68 – 7.64 (m, 1H), 7.62 – 7.58 (m, 1H), 7.52 (d, *J* = 7.4 Hz, 1H), 7.27 – 7.19 (m, 8H), 6.91 – 6.84 (m, 5H), 6.71 (s, 2H), 2.24 (s, 3H), 2.06 (s, 6H). **^{11}B NMR (128 MHz, CD_2Cl_2)** δ = 60.3. **^{13}C{^{1}H} NMR (126 MHz, CD_2Cl_2)** δ = 156.8, 149.4 (br, B-***C***), 145.5, 144.9, 144.5, 141.7, 141.6, 141.4 (br, B-***C***), 141.1, 140.2, 138.1, 136.8, 136.4, 136.0 (br, B-***C***), 135.4, 133.7, 131.0, 130.0, 130.0, 129.6, 129.4, 127.9, 127.2, 127.2, 127.1, 125.1, 123.4, 123.2, 121.0, 120.8, 23.4, 21.4. **HRMS (LDI)** *m/z* calcd for $C_{39}H_{29}B$ $[M]^+$ 508.2357, found 508.2376.

General procedure B: Lithium-Halogen Exchange and Negishi cross-coupling reactions

An oven-dried J Young flask was charged with the bromoarylamine compound (1 equiv.). The flask was evacuated and backfilled with nitrogen gas three times. The solid was then dissolved in dry THF (~ 2 mL per mmol). The solution was cooled to -78 °C and *n*-BuLi (1.1 equiv., 1.6 M in hexane) was added dropwise. The reaction was stirred at -78 °C for 1 hour. Then a solution of $ZnCl_2$ (1.1 equiv., ~ 0.8 M in THF) was added dropwise at -78 °C. The reaction was stirred at -78 °C for 20 minutes and brought to room temperature and stirred for another 10 minutes. The mixture was then transferred to another J Young flask charged with **BPAH** (0.67 equiv.), $Pd_2(dba)_3$ (0.02 equiv.), and SPhos (0.08 equiv.). The reaction was heated at 70 °C in an oil bath for 20 hours and then quenched with saturated NH_4Cl aqueous solution. The product was extracted with ethyl acetate and the organic layer was dried over Na_2SO_4 and concentrated in *vacuo*. The crude product was purified by column chromatography on silica gel.

Synthesis of compound 4

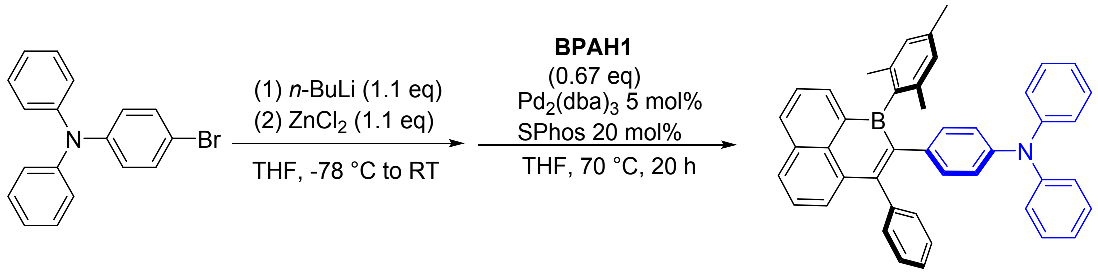

The synthesis was performed according to the **General Procedure B** using 4-bromotriphenylamine (243 mg, 0.75 mmol), *n*-BuLi (0.5 mL, 0.8 mmol), $ZnCl_2$ (112 mg, 0.83 mmol), **BPAH1** (219 mg, 0.5 mmol), $Pd_2(dba)_3$ (14 mg, 0.015 mmol) and SPhos (25 mg, 0.06 mmol). The desired product was obtained as an orange solid after column chromatography (silica, dichloromethane: hexane 1:100 to 1:10). Yield: 241 mg (0.4 mmol, 80%).

^{1}H NMR (500 MHz, CD_2Cl_2) δ = 8.33 (dd, *J* = 8.1, 1.5 Hz, 1H), 8.13 (dd, *J* = 6.9, 1.5 Hz, 1H), 8.11 (dd, *J* = 8.1, 1.2 Hz, 1H), 7.75 – 7.72 (m, 2H), 7.59 (t, *J* = 7.7 Hz, 1H), 7.33 – 7.29 (m, 3H), 7.26 – 7.24 (m, 2H), 7.19 – 7.15 (m, 4H), 6.93 (tt, *J* = 7.3, 1.2 Hz, 2H), 6.87 – 6.81 (m, 4H), 6.77 (s, 2H), 6.75 – 6.73 (m, 2H), 6.61 – 6.58 (m, 2H), 2.30 (s, 3H), 2.00 (s, 6H). **^{11}B NMR (160 MHz, CD_2Cl_2)** δ = 59.7. **^{13}C{^{1}H} NMR (126 MHz, CD_2Cl_2)** δ = 157.7, 148.8 (br, B-***C***), 148.5, 144.7, 143.6, 142.0 (br, B-***C***), 141.3, 140.6, 138.5, 136.8 (136.82), 136.8 (136.79), 135.6 (br, B-***C***), 134.8, 134.6, 132.6, 132.4, 131.6, 130.8, 130.6, 129.5, 127.7, 127.2, 127.1 (127.09), 127.1 (127.06), 126.3, 124.0, 123.8, 122.6, 23.7, 21.5. **HRMS (LDI)** *m/z* calcd for $C_{45}H_{36}BN$ $[M]^+$ 601.2943, found 601.2946.

Synthesis of compound 5

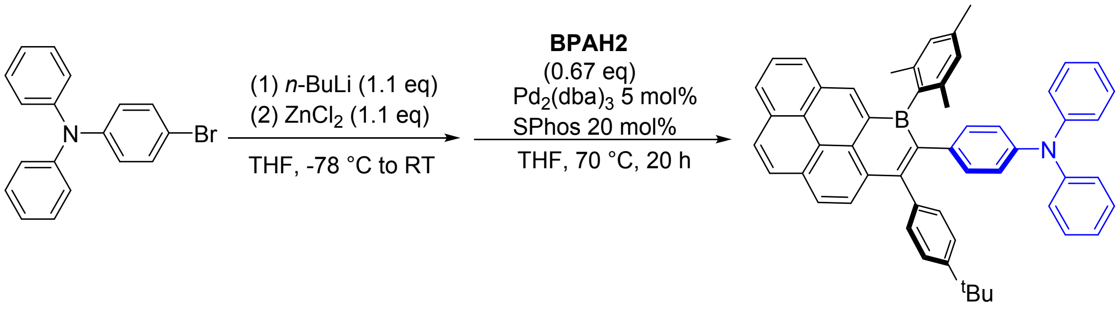

The synthesis was performed according to the **General Procedure B** using 4-bromotriphenylamine (389 mg, 1.2 mmol), *n*-BuLi (0.83 mL, 1.32 mmol), $ZnCl_2$ (180 mg, 1.32 mmol), **BPAH2** (454 mg, 0.8 mmol), $Pd_2(dba)_3$ (22 mg, 0.024 mmol) and SPhos (39 mg, 0.096 mmol). The desired product was obtained as a dark red solid after column chromatography (silica, dichloromethane: hexane 1:100 to 1:10). Yield: 340 mg (0.46 mmol, 58%). **^{1}H NMR (500 MHz, CD_2Cl_2)** δ = 9.00 (s, 1H), 8.56 – 8.42 (m, 2H), 8.24 – 8.19 (m, 4H), 8.14 (t, *J* = 7.6 Hz, 1H), 7.45 – 7.33 (m, 2H), 7.29 – 7.23 (m, 2H), 7.21 – 7.12 (m, 4H), 6.97 – 6.90 (m, 2H), 6.86 (dd, *J* = 8.6, 1.0 Hz, 4H), 6.83 (s, 2H), 6.80 – 6.74 (m, 2H), 6.65 – 6.57 (m, 2H), 2.36 (s, 3H), 2.08 (s, 6H), 1.39 (s, 9H). **^{11}B NMR (160 MHz, CD_2Cl_2)** δ = 59.7. **^{13}C{^{1}H} (126 MHz, CD_2Cl_2)** δ = 159.4, 150.3, 148.7 (br, B-***C***), 148.5, 148.4, 144.4, 142.2 (br, B-***C***), 141.0, 138.9, 138.6, 136.8, 135.4 (br, B-***C***), 133.3, 131.8, 131.4, 131.3, 131.2 (two peaks overlapped), 131.0 (131.03), 131.0 (130.99), 130.6, 129.5, 128.5, 128.5, 128.3, 127.1, 127.0, 126.2, 124.9, 124.7, 124.0, 123.9, 123.7, 122.5, 35.0, 31.8, 23.9, 21.6. **HRMS (LDI)** *m/z* calcd for $C_{55}H_{46}BN$ $[M]^+$ 731.3723, found 731.3738.

Synthesis of compound 6

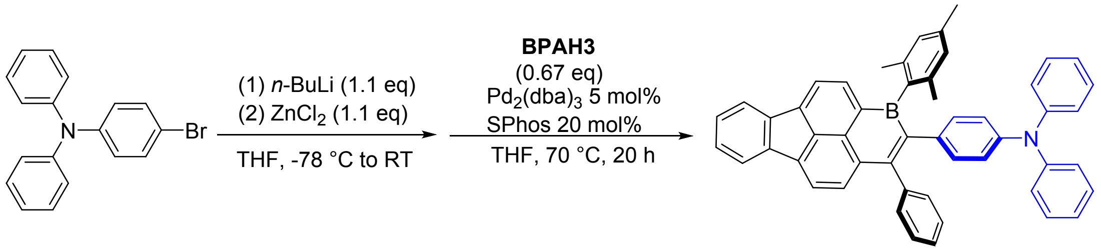

The synthesis was performed according to the **General Procedure B** using 4-bromotriphenylamine (340 mg, 1.05 mmol), *n*-BuLi (0.73 mL, 1.16 mmol), $ZnCl_2$ (160 mg, 1.16 mmol), **BPAH3** (359 mg, 0.7 mmol), $Pd_2(dba)_3$ (19 mg, 0.021 mmol) and SPhos (35 mg, 0.08 mmol). The crude product was purified by column chromatography (silica, dichloromethane: hexane 1:100 to 1:4) followed by washing with hexane. Yield: 223 mg (0.33 mmol, 47%), amorphous solid.

^{1}H NMR (500 MHz, CD_2Cl_2) δ = 7.83 (d, *J* = 6.9 Hz, 1H), 7.71 (d, *J* = 6.9 Hz, 1H), 7.66 – 7.64 (m, 1H), 7.62 – 7.60 (m, 1H), 7.53 (d, *J* = 7.3 Hz, 1H), 7.34 (d, *J* = 7.4 Hz, 1H), 7.32 – 7.28 (m, 3H), 7.25 – 7.21 (m, 4H), 7.19 – 7.15 (m, 4H), 6.93 (tt, *J* = 7.3, 1.1 Hz, 2H), 6.83 – 6.81 (m, 4H), 6.76 (s, 2H), 6.72 – 6.69 (m, 2H), 6.59 – 6.56 (m, 2H), 2.29 (s, 3H), 2.06 (s, 6H). **^{11}B NMR (128 MHz, CD_2Cl_2)** δ = 60.5. **^{13}C{^{1}H} NMR (126 MHz, CD_2Cl_2)** δ = 156.7, 149.2 (br, B-***C***), 148.5, 145.5, 144.8, 144.5, 141.7, 141.6, 141.5 (br, B-***C***), 141.0, 140.3, 140.2, 138.3, 136.9, 136.3, 136.1 (br, B-***C***), 135.3, 133.7, 131.2, 130.8, 130.1, 130.0, 129.5, 129.4, 127.9, 127.23, 127.1, 123.9 (123.89), 123.9 (123.87), 123.4, 123.2, 122.6, 121.0, 120.8, 23.5, 21.5. **HRMS (LDI)** *m/z* calcd for $C_{51}H_{38}BN$ $[M]^+$ 675.3092, found 675.3124.

Synthesis of compound 7

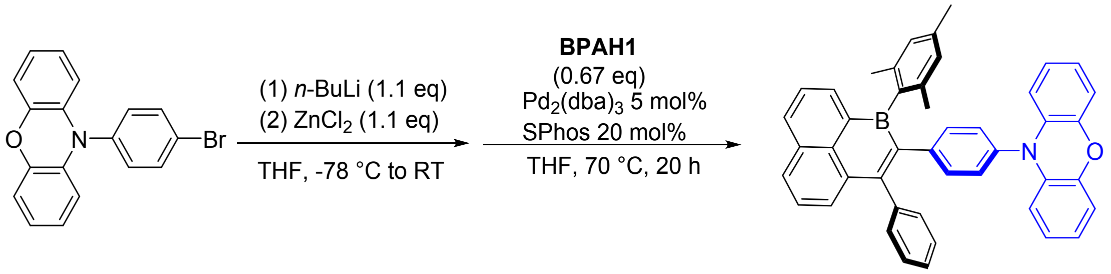

The synthesis was performed according to the **General Procedure B** using 10-(4-bromophenyl)-10*H*-phenoxazine (507 mg, 1.5 mmol), *n*-BuLi (1.03 mL, 1.65 mmol), $ZnCl_2$ (224 mg, 1.65 mmol), **BPAH1** (437 mg, 1 mmol), $Pd_2(dba)_3$ (29 mg, 0.03 mmol) and SPhos (50 mg, 0.12 mmol). The desired product was obtained as an orange solid after column chromatography (silica, dichloromethane: hexane 1:100 to 1:10). Yield: 420 mg (0.68 mmol, 68%). **^{1}H NMR (500 MHz, $CDCl_3$)** δ = 8.34 (dd, *J* = 8.1, 1.4 Hz, 1H), 8.29 (dd, *J* = 6.8, 1.4 Hz, 1H), 8.13 (dd, *J* = 8.1, 1.2 Hz, 1H), 7.83 (dd, *J* = 7.4, 1.2 Hz, 1H), 7.76 (dd, *J* = 8.0, 6.8 Hz, 1H), 7.62 (t, *J* = 7.8 Hz, 1H), 7.32 – 7.27 (m, 5H), 6.99 – 6.99 (m, 2H), 6.81 – 6.79 (m, 2H), 6.75 (s, 2H), 6.62 – 6.57 (m, 4H), 6.54 (ddd, *J* = 7.8, 6.9, 2.1 Hz, 2H), 5.53 (dd, *J* = 7.8, 1.5 Hz, 2H), 2.28 (s, 3H), 2.04 (s, 6H). **^{11}B NMR (128 MHz, $CDCl_3$)** δ = 59.0. **^{13}C{^{1}H} NMR (126 MHz, $CDCl_3$)** δ = 157.3, 148.3 (br, B-***C***), 145.2, 144.0, 143.7, 141.2 (br, B-***C***), 140.4, 138.1, 136.6, 136.5, 135.0 (br, B-***C***), 134.9, 134.7, 134.7, 133.8, 132.5, 132.0, 131.8, 131.2, 130.3, 128.8, 127.4, 126.9 (126.93), 126.9 (126.87), 126.7, 126.0, 123.2, 121.0, 115.2, 113.2, 23.7, 21.4. **HRMS (LDI)** *m/z* calcd for $C_{45}H_{34}BNO$ $[M]^+$ 615.2736, found 615.2750.

Synthesis of compound 8

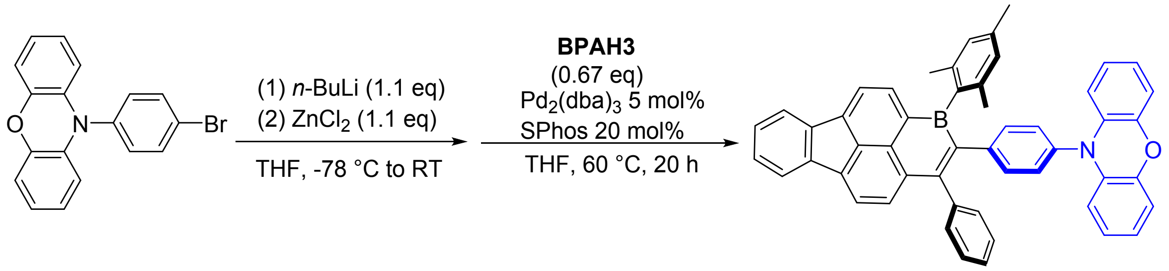

The synthesis was performed according to the **General Procedure B** using 10-(4-bromophenyl)-10*H*-phenoxazine (254 mg, 0.75 mmol), *n*-BuLi (0.83 mmol), $ZnCl_2$ (112 mg, 0.83 mmol), **BPAH3** (256 mg, 0.5 mmol), $Pd_2(dba)_3$ (14 mg, 0.015 mmol) and SPhos (25 mg, 0.03 mmol). The desired product was obtained as a red solid after column chromatography (silica, dichloromethane: hexane 1:100 to 1:10). Yield: 210 mg (0.3 mmol, 61%).

^{1}H NMR (500 MHz, CD_2Cl_2) δ = 7.92 (d, *J* = 6.9 Hz, 1H), 7.75 (d, *J* = 6.9 Hz, 1H), 7.68 – 7.66 (m, 1H), 7.64 -7.62 (m, 1H), 7.56 (d, *J* = 7.3 Hz, 1H), 7.41 (d, *J* = 7.3 Hz, 1H), 7.34 – 7.20 (m, 7H), 7.02 – 7.00 (m, 2H), 6.80 – 6.77 (m, 2H), 6.75 (s, 2H), 6.60 – 6.52 (m, 6H), 5.51 – 5.50 (m, 2H), 2.26 (s, 3H), 2.10 (s, 6H). **^{11}B NMR (160 MHz, CD_2Cl_2)** δ = 60.1. **^{13}C{^{1}H} NMR (126 MHz, CD_2Cl_2)** δ = 157.0, 149.1 (br, B-*C*), 145.7, 145.6, 144.8, 144.4, 141.7, 141.6, 141.4, 141.2 (br, B-*C*), 140.0, 138.3, 137.1, 136.0, 135.9 (br, B-*C*), 135.6, 135.5, 135.1, 133.8, 132.4, 131.2, 130.2, 130.1, 129.5, 129.2, 128.0, 127.5, 127.1, 123.7, 123.5, 123.3, 121.4, 121.1, 120.8, 115.5, 113.6, 23.6, 21.5. **HRMS (LDI)** *m/z* calcd for $C_{51}H_{36}BNO$ $[M]^+$ 689.2893, found 689.2904.

Synthesis of 3-(2-Phenylethynyl)fluoranthene

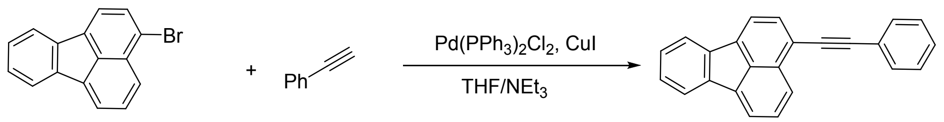

To a mixture of 3-bromofluoranthene (2.81 g, 10 mmol, 1 equiv.), copper(I) iodide (290 mg, 1.5 mmol, 0.15 equiv.) and $Pd(PPh_3)_2Cl_2$ (350 mg, 0.5 mmol, 0.05 equiv.) was added dry THF (23 mL), dry triethylamine (7 mL) and phenylacetylene (1.7 mL, 15 mmol, 1.5 equiv.). The mixture was heated to 80 °C in an oil bath for 40 hours in a sealed vessel. Volatiles were removed in *vacuo* and the mixture was taken up in dichloromethane. The reaction mixture was washed with saturated aqueous ammonium chloride, then brine and the organic layer was collected and dried with Na_2SO_4. The product was separated as a yellow solid by flash column chromatography using a DCM: hexane (1:100 to 1:5) eluent. Yield: 56%.

^{1}H NMR (500 MHz, $CDCl_3$) δ = 8.23 (dd, *J* = 8.3, 0.7 Hz, 1H), 7.94 (dd, *J* = 6.9, 0.6 Hz, 1H), 7.92 – 7.82 (m, 4H), 7.73 – 7.63 (m, 3H), 7.50 – 7.35 (m, 5H). **^{13}C{^{1}H} NMR (126 MHz, $CDCl_3$)** δ = 139.9, 139.1, 137.4, 137.3, 132.4, 132.3, 131.9, 130.6, 128.7, 128.6, 128.6, 128.0, 127.9, 125.5, 123.5, 121.9, 121.8, 120.7, 120.7, 119.9, 95.1, 87.5. **HRMS (EI)** *m/z* calcd for $C_{24}H_{14}$ $[M]^+$ 302.1090, found 302.1092.

S3. NMR Spectra

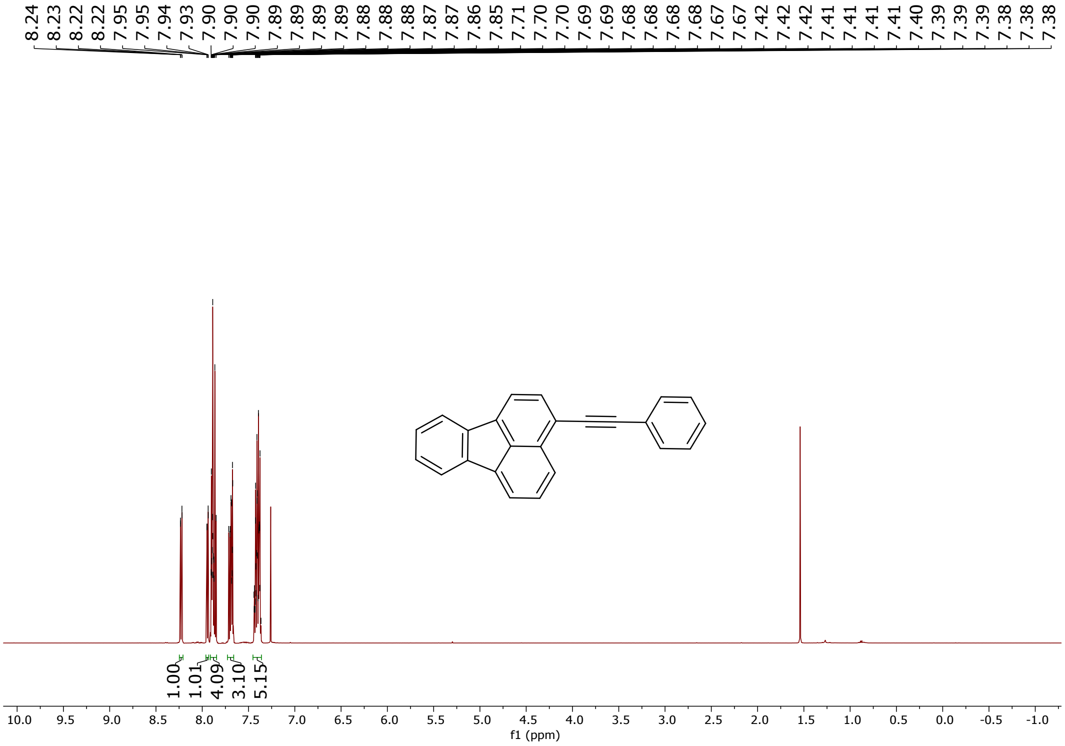

Figure **S1**. ^{1}H NMR spectrum of 3-(2-phenylethynyl)fluoranthene in $CDCl_3$ (500 MHz).

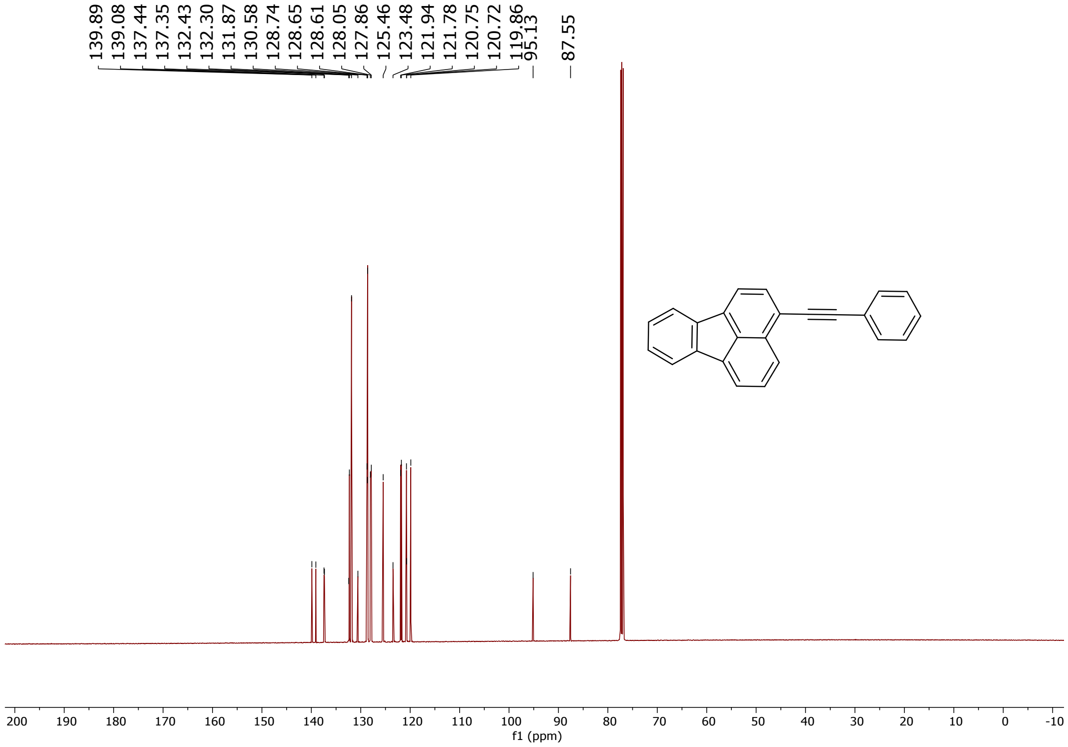

Figure **S2**. $^{13}C\{^{1}H\}$ NMR spectrum of 3-(2-phenylethynyl)fluoranthene in $CDCl_3$ (126 MHz).

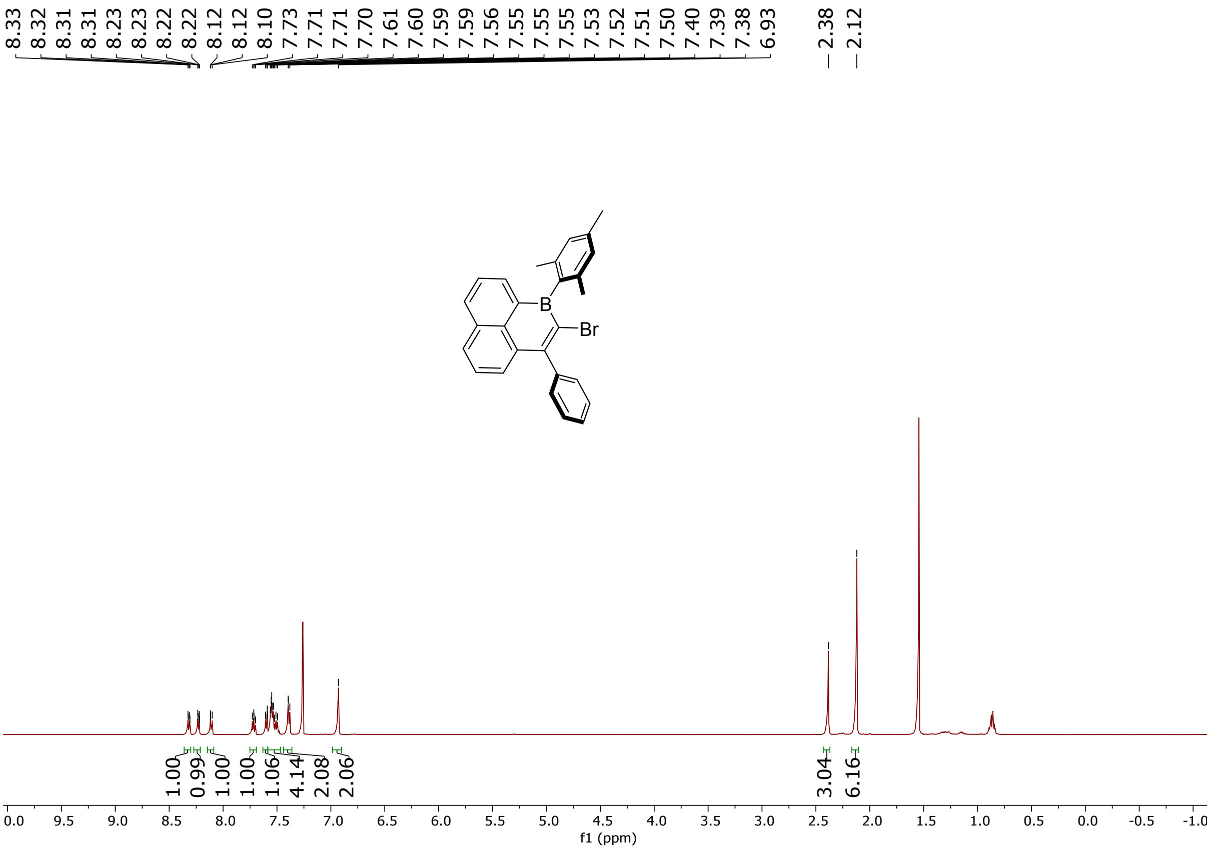

Figure **S3**. ^{1}H NMR spectrum of **BPAH1** in $CDCl_3$ (500 MHz).

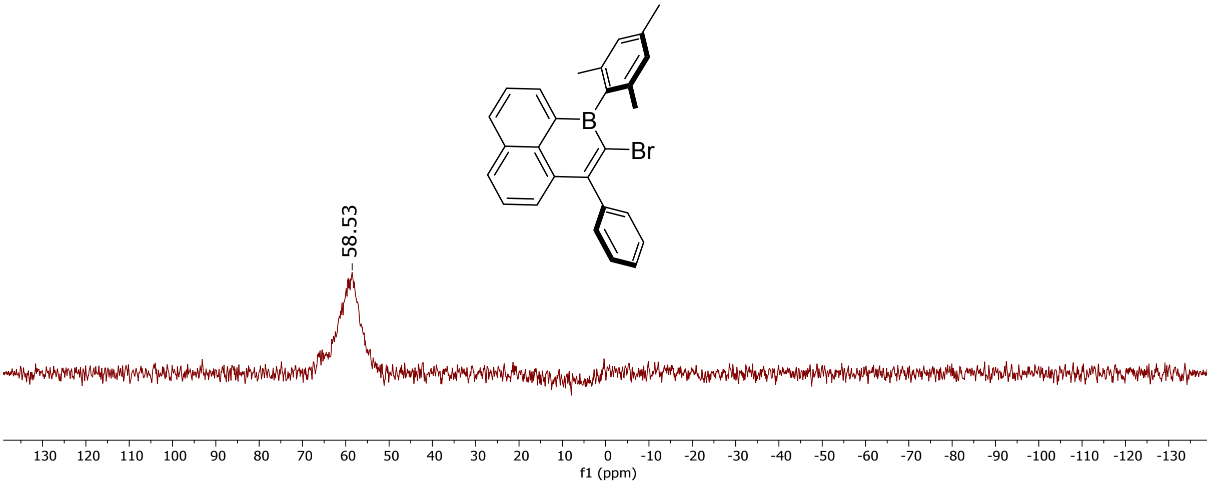

Figure **S4**. ^{11}B NMR spectrum of **BPAH1** in $CDCl_3$ (160 MHz).

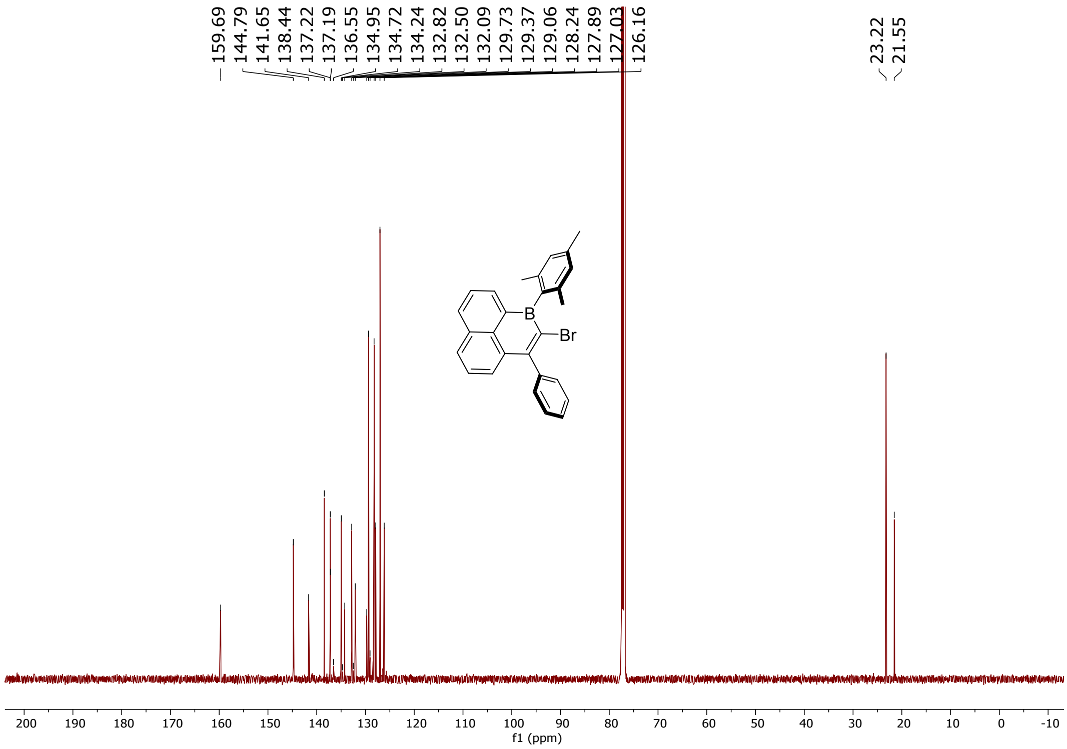

Figure **S5**. $^{13}C\{^{1}H\}$ NMR spectrum of **BPAH1** in $CDCl_3$ (126 MHz).

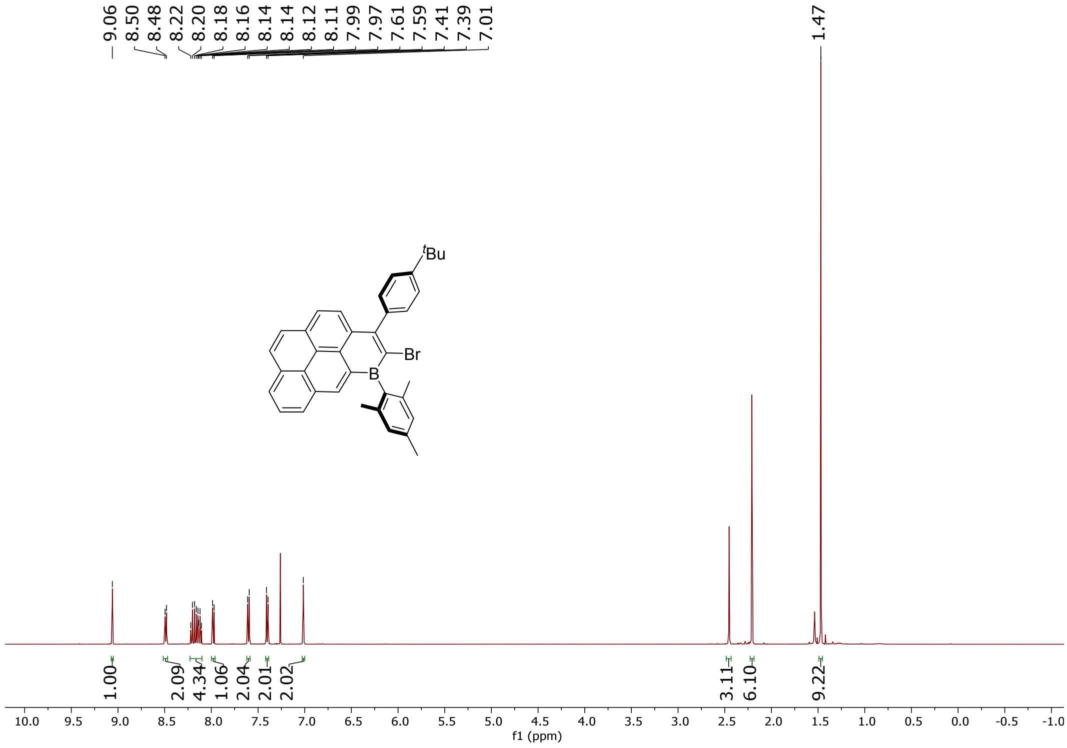

Figure **S6**. ^{1}H NMR spectrum of **BPAH2** in $CDCl_3$ (500 MHz).

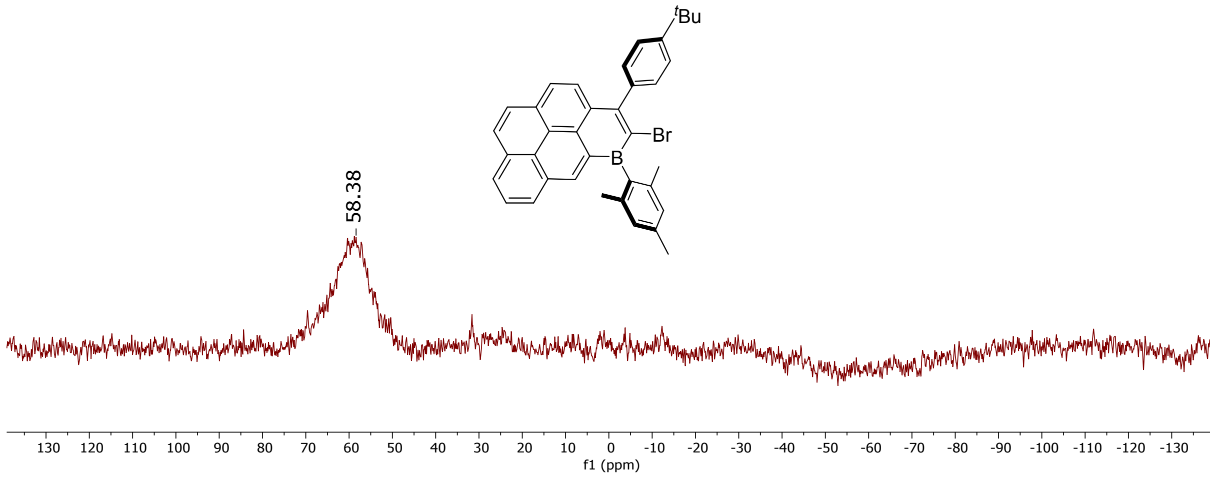

Figure **S7**. ^{11}B NMR spectrum of **BPAH2** in $CDCl_3$ (160 MHz).

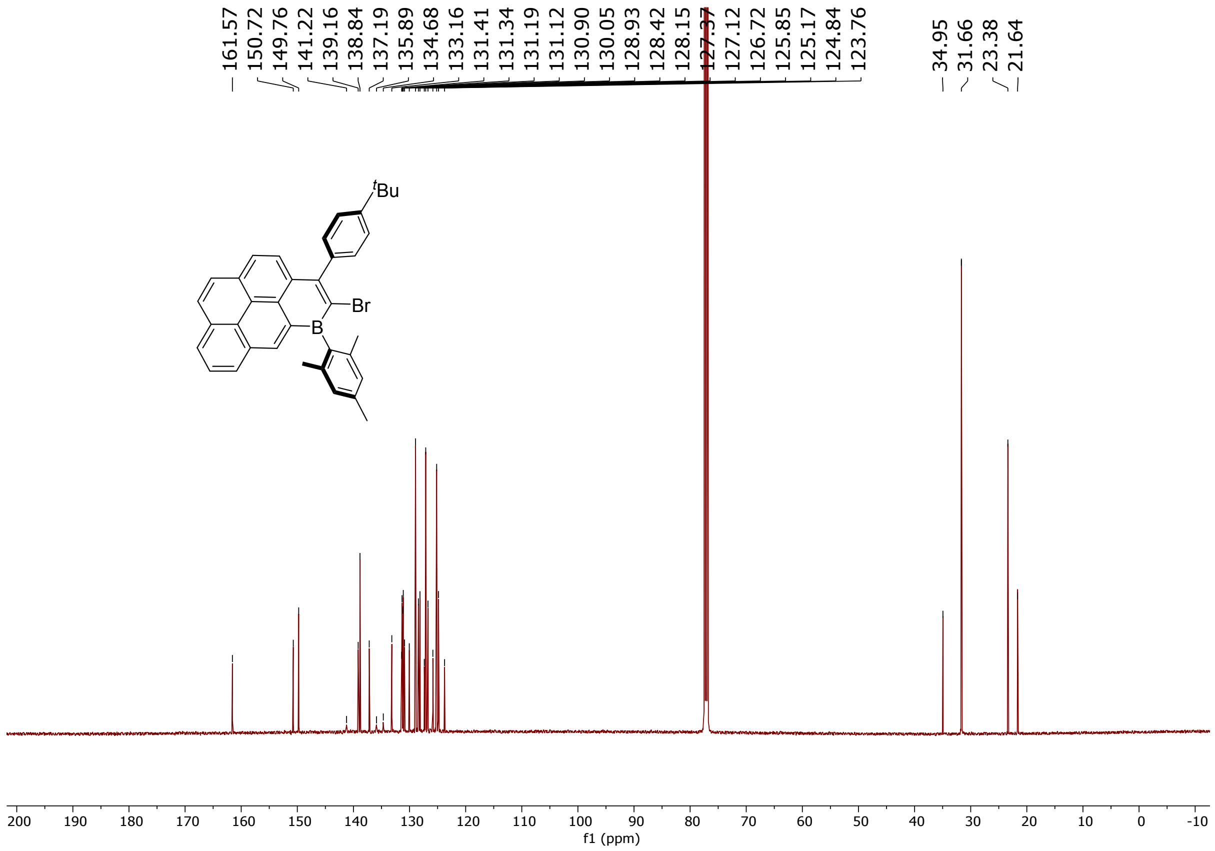

Figure **S8**. ^{13}C{^{1}H} NMR spectrum of **BPAH2** in $CDCl_3$ (126 MHz).

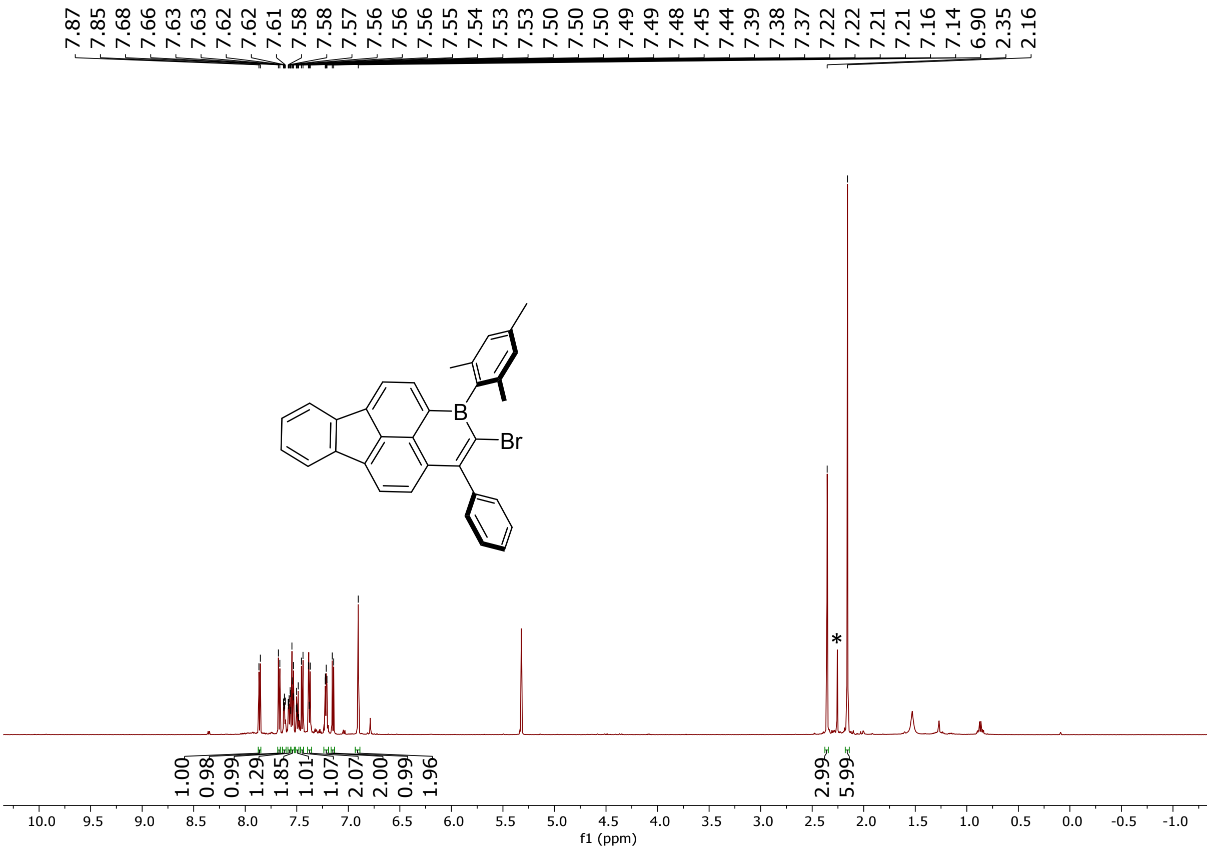

Figure **S9**. ^{1}H NMR spectrum of **BPAH3** in CD_2Cl_2(*contains 8% mesitylene) (500 MHz).

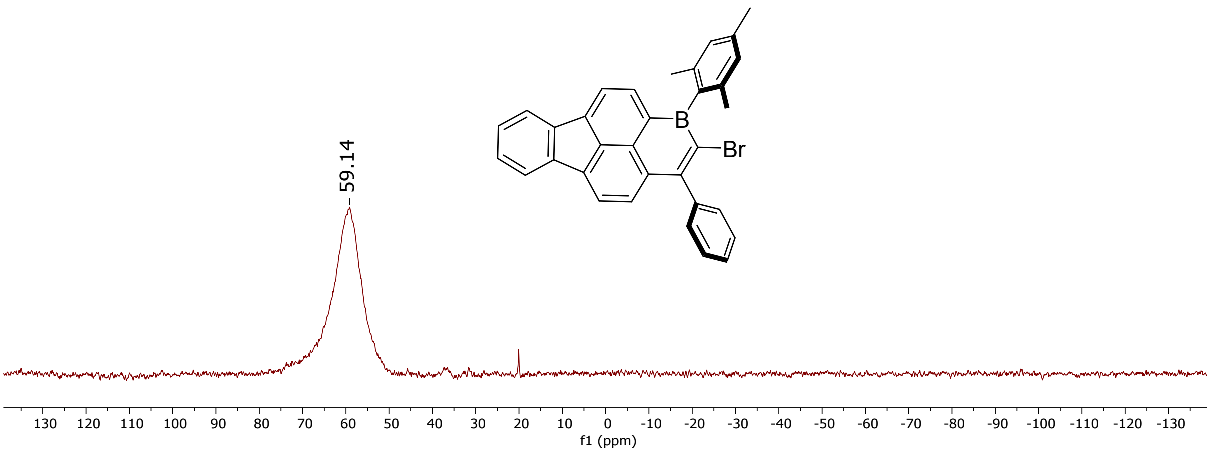

Figure **S10**. ^{11}B NMR spectrum of **BPAH3** in CD_2Cl_2 (160 MHz).

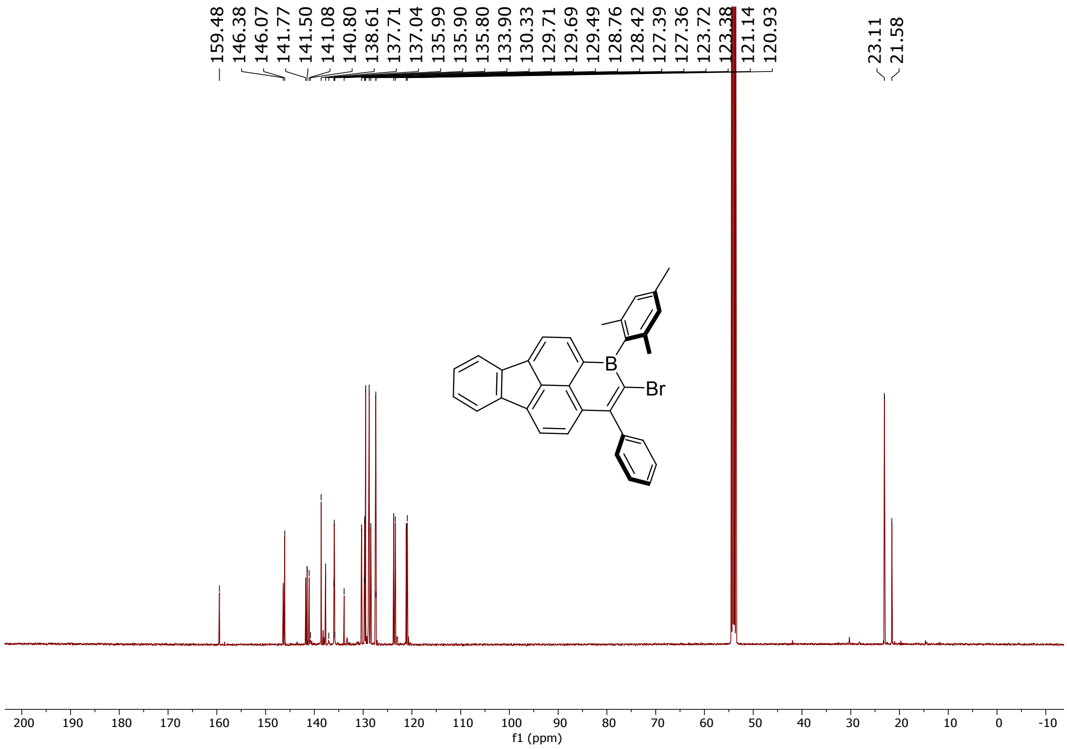

Figure **S11**. $^{13}C\{^{1}H\}$ NMR spectrum of **BPAH3** in CD_2Cl_2 (126 MHz).

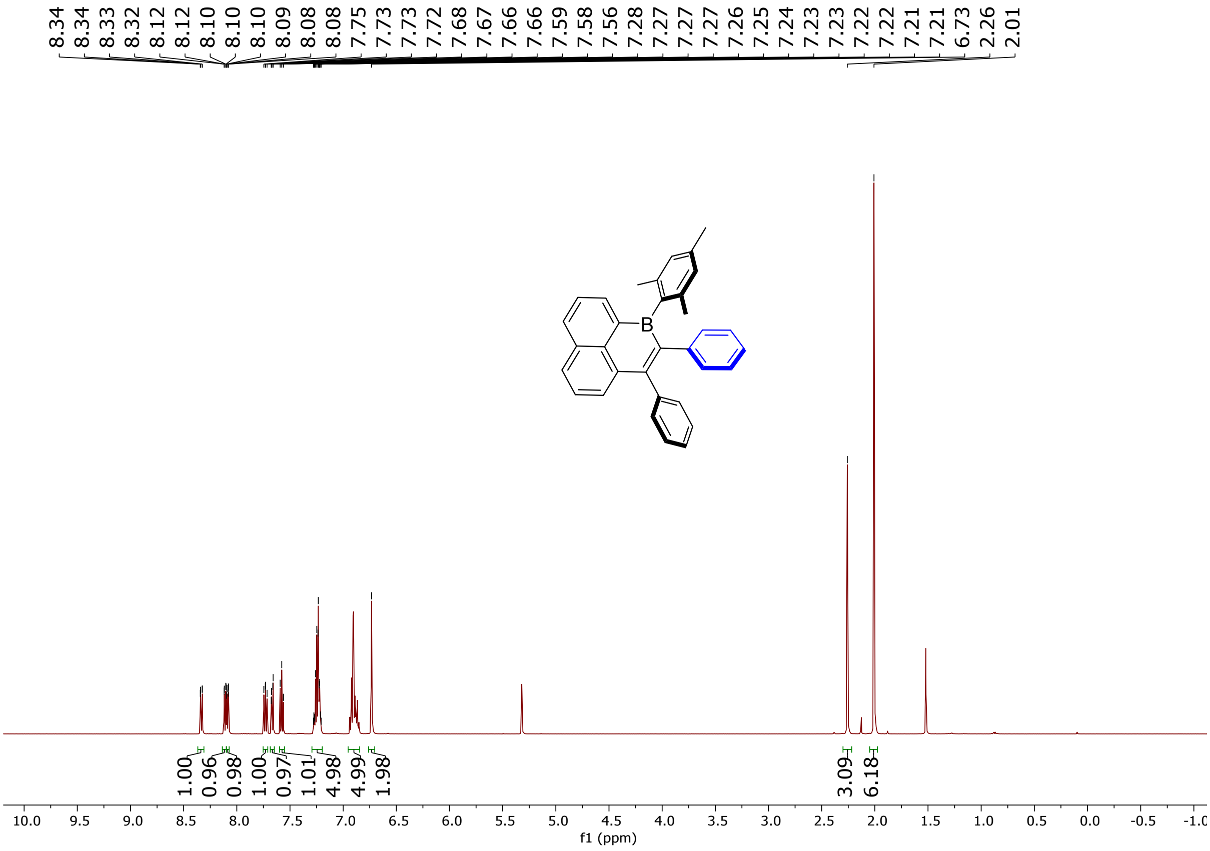

Figure **S12**. ^{1}H NMR spectrum of compound **1** in CD_2Cl_2 (500 MHz).

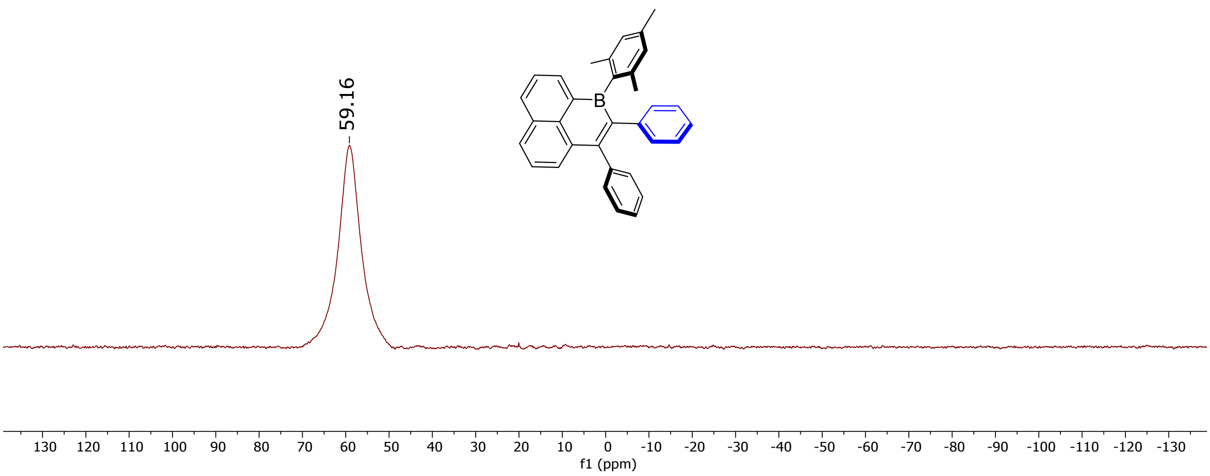

Figure **S13**. ^{11}B NMR spectrum of compound **1** in CD_2Cl_2 (160 MHz).

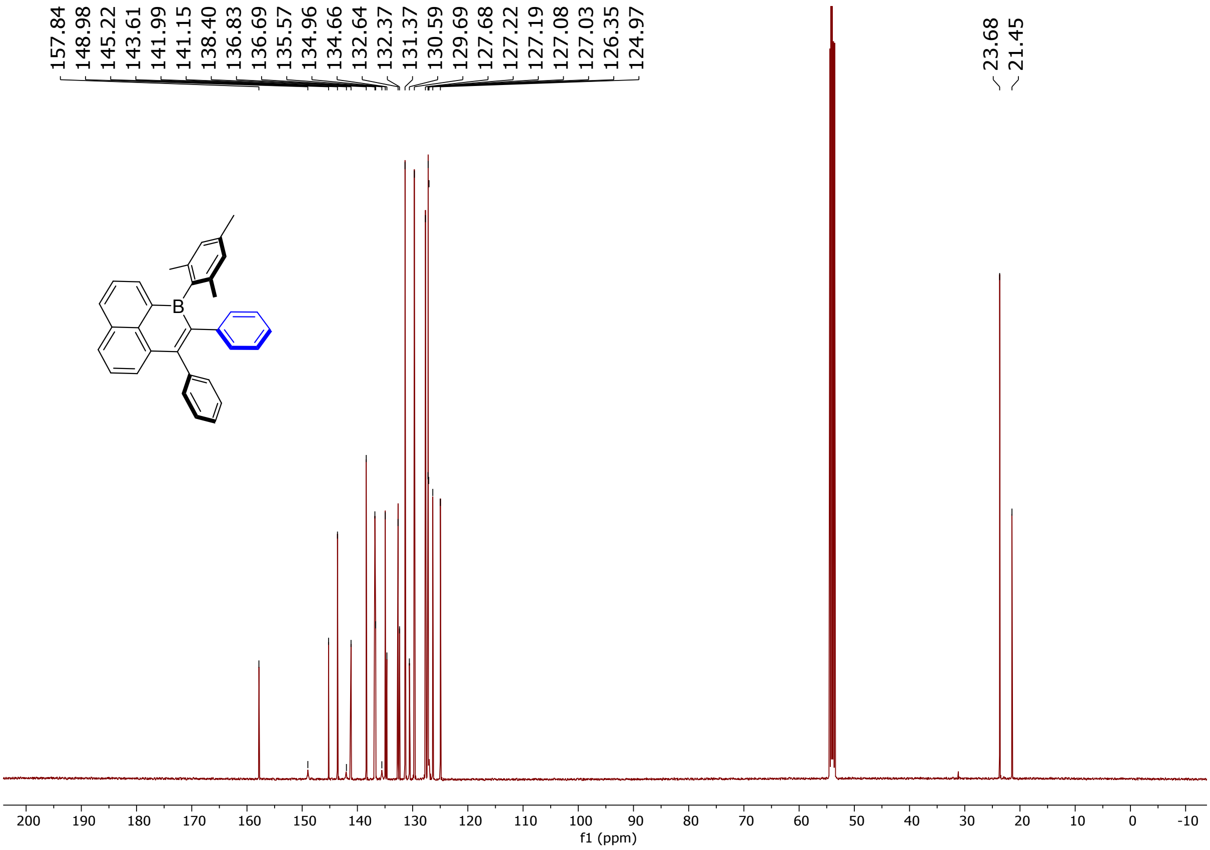

Figure **S14**. $^{13}C\{^1H\}$ NMR spectrum of compound **1** in CD_2Cl_2 (126 MHz).

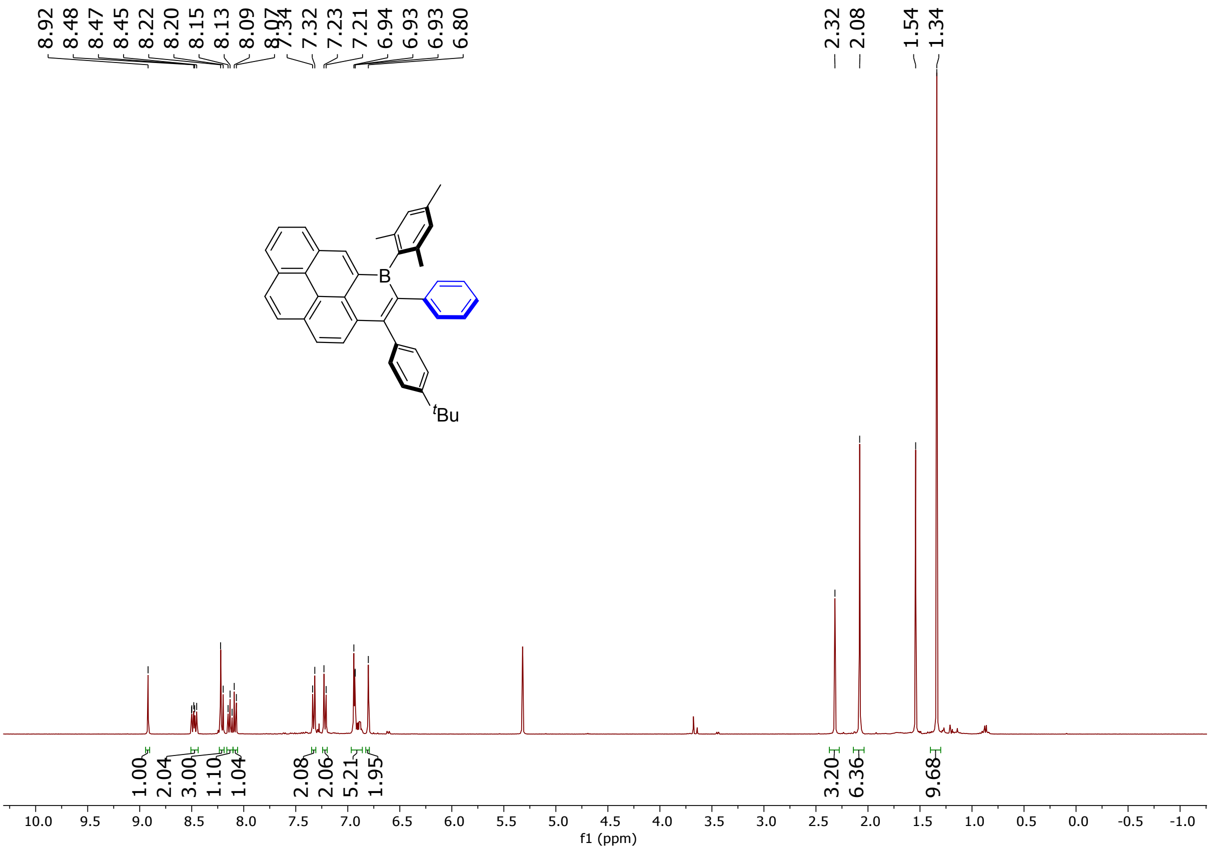

Figure **S15**. ^{1}H NMR spectrum of compound **2** in CD_2Cl_2 (400 MHz).

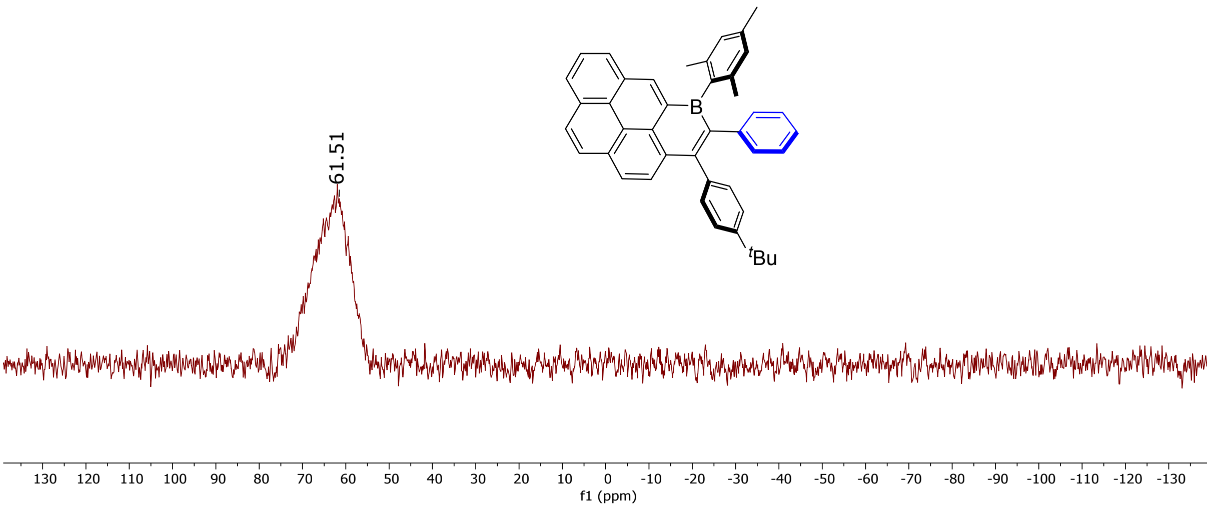

Figure **S16**. ^{11}B NMR spectrum of compound **2** in CD_2Cl_2 (128 MHz).

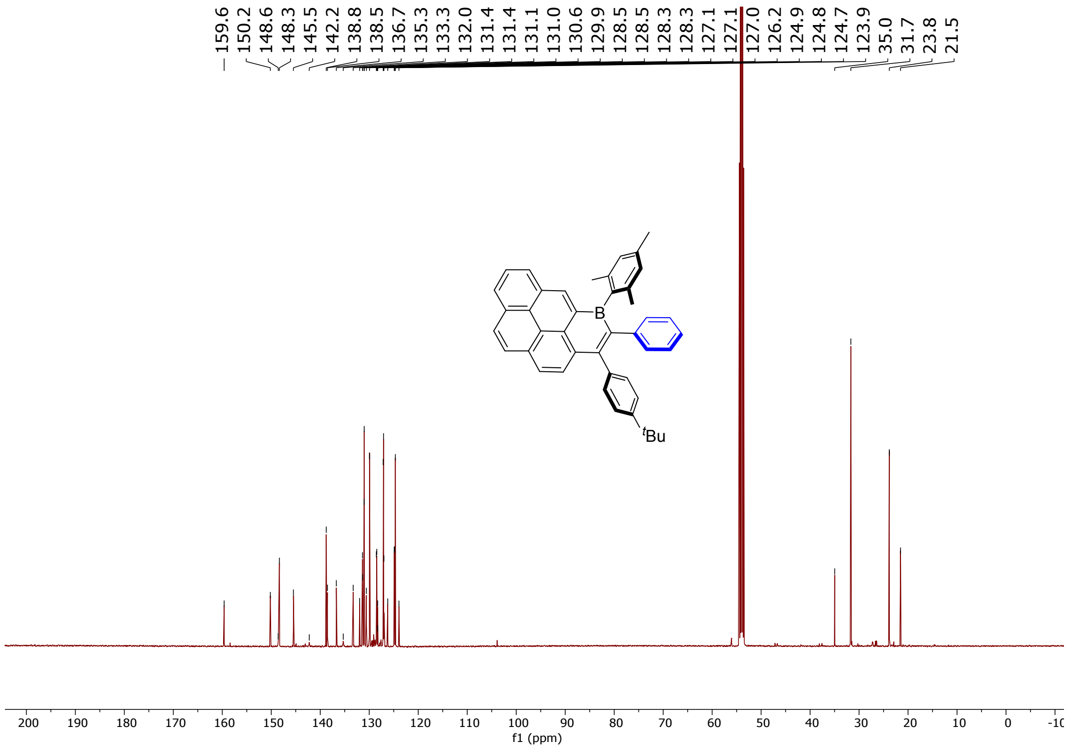

Figure **S17**. $^{13}C\{^1H\}$ NMR spectrum of compound **2** in CD_2Cl_2 (126 MHz).

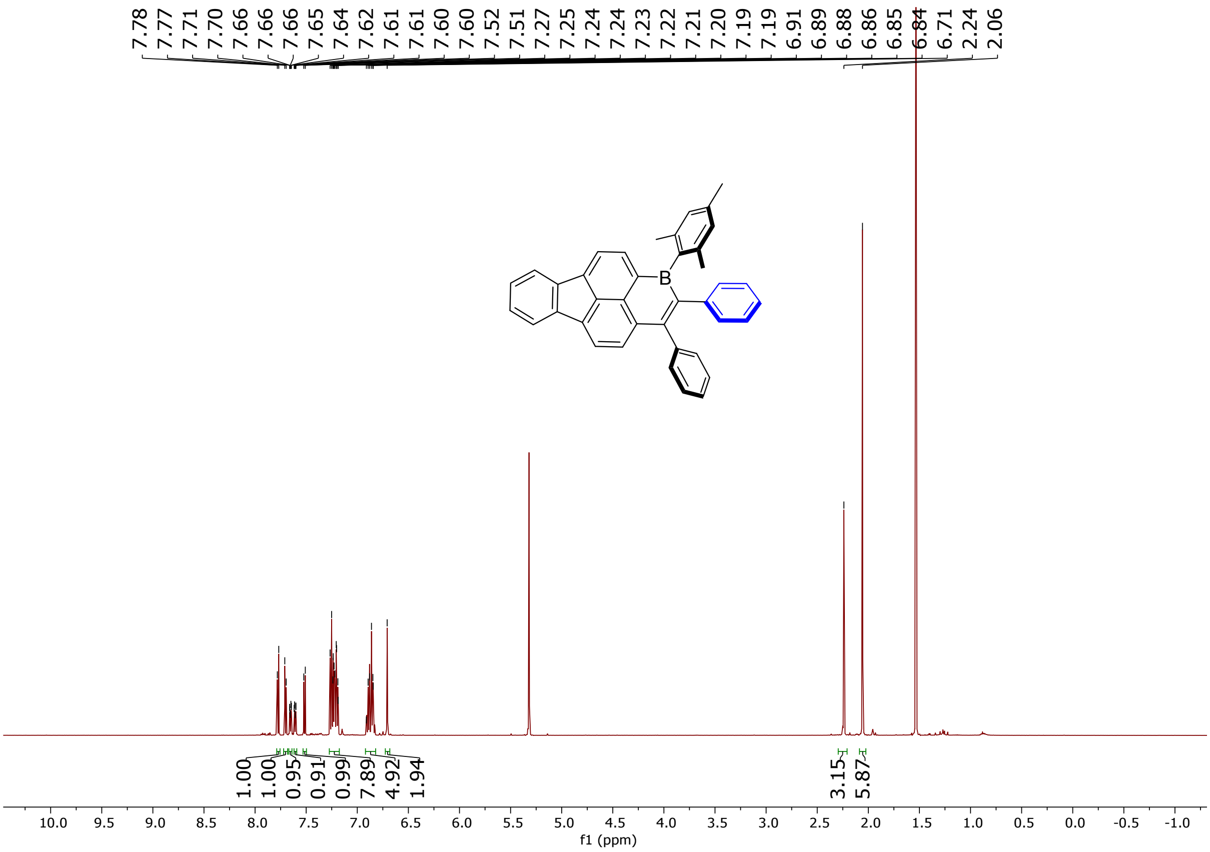

Figure **S18**. ^1H NMR spectrum of compound **3** in CD_2Cl_2 (500 MHz).

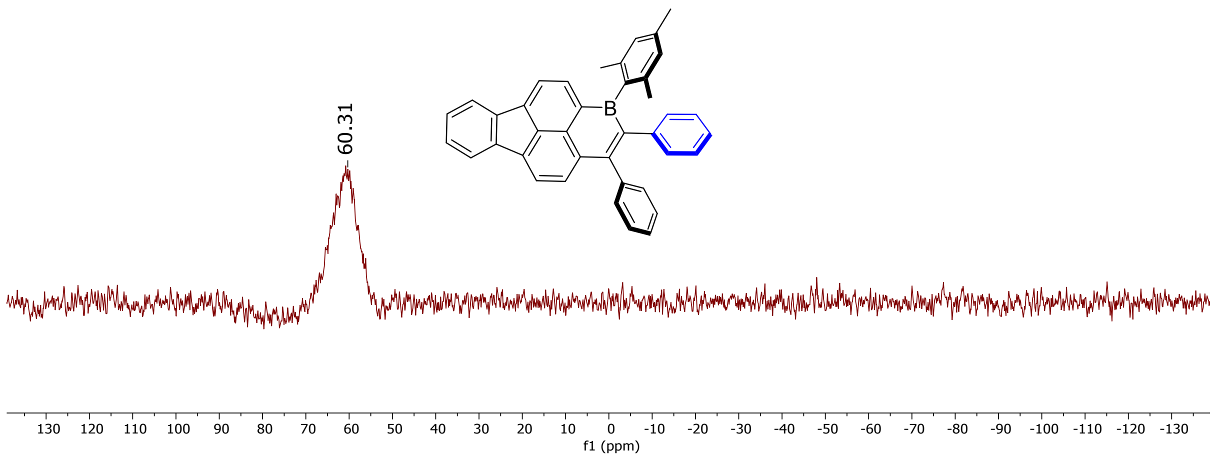

Figure **S19**. ^{11}B NMR spectrum of compound **3** in CD_2Cl_2 (128 MHz).

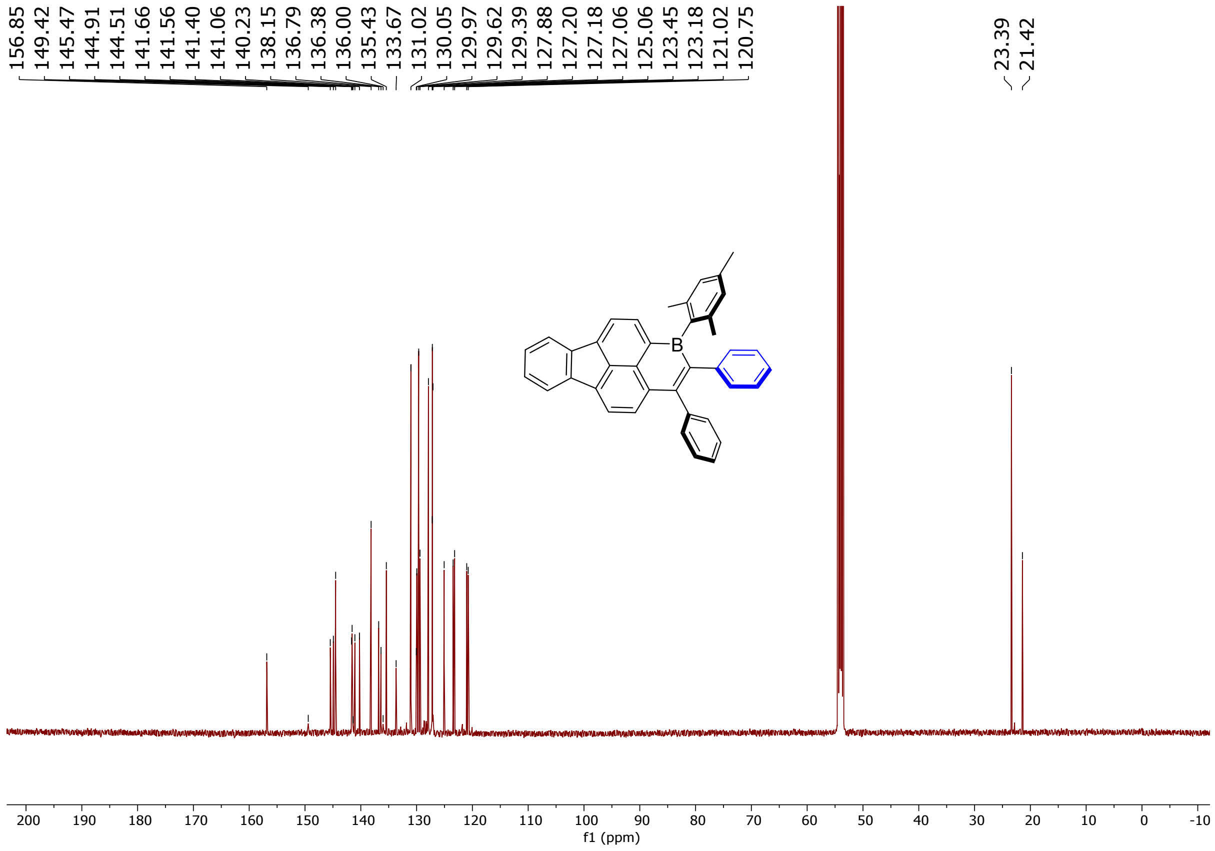

Figure **S20**. $^{13}C\{^{1}H\}$ NMR spectrum of compound **3** in CD_2Cl_2 (126 MHz).

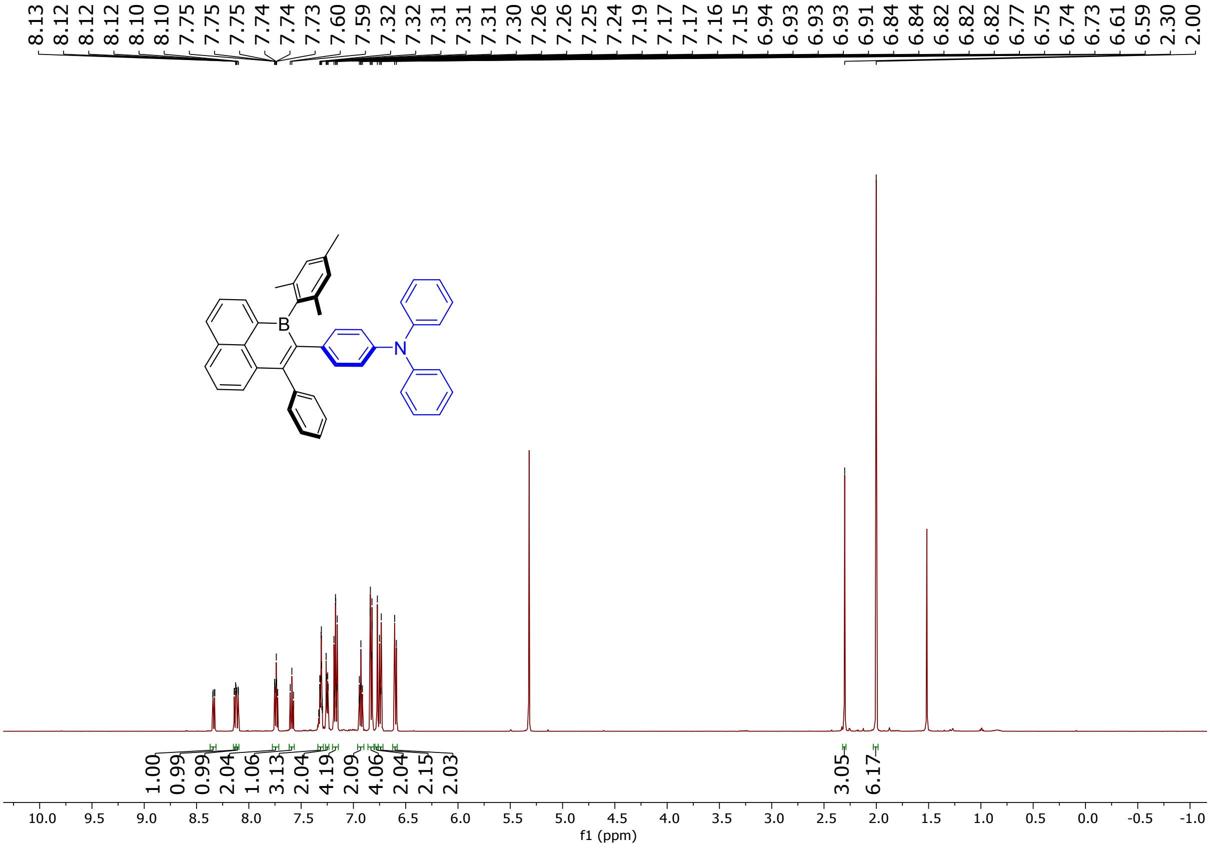

Figure **S21**. ^{1}H NMR spectrum of compound **4** in CD_2Cl_2 (500 MHz).

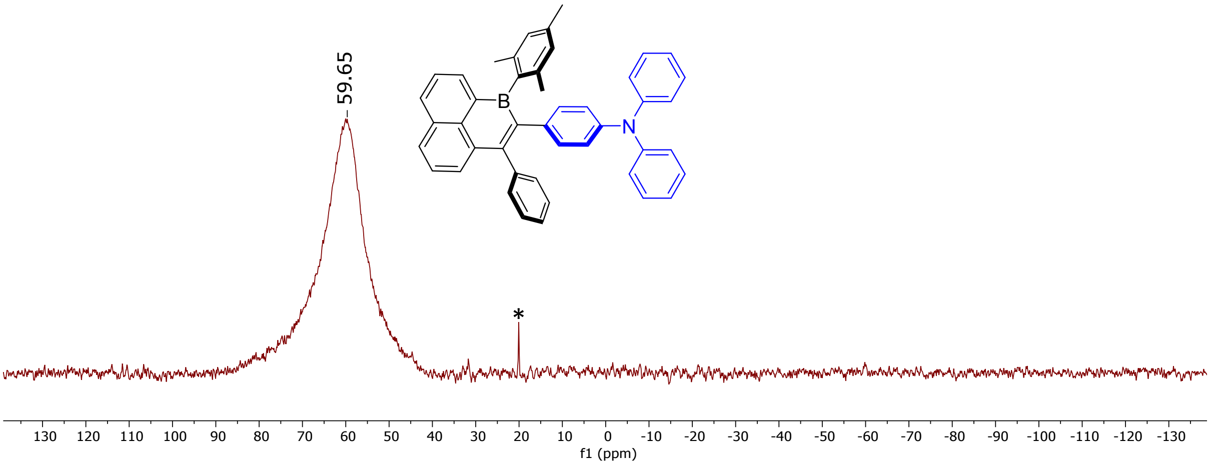

Figure **S22**. ^{11}B NMR spectrum of compound **4** in CD_2Cl_2(*$B(OH)_3$) (160 MHz).

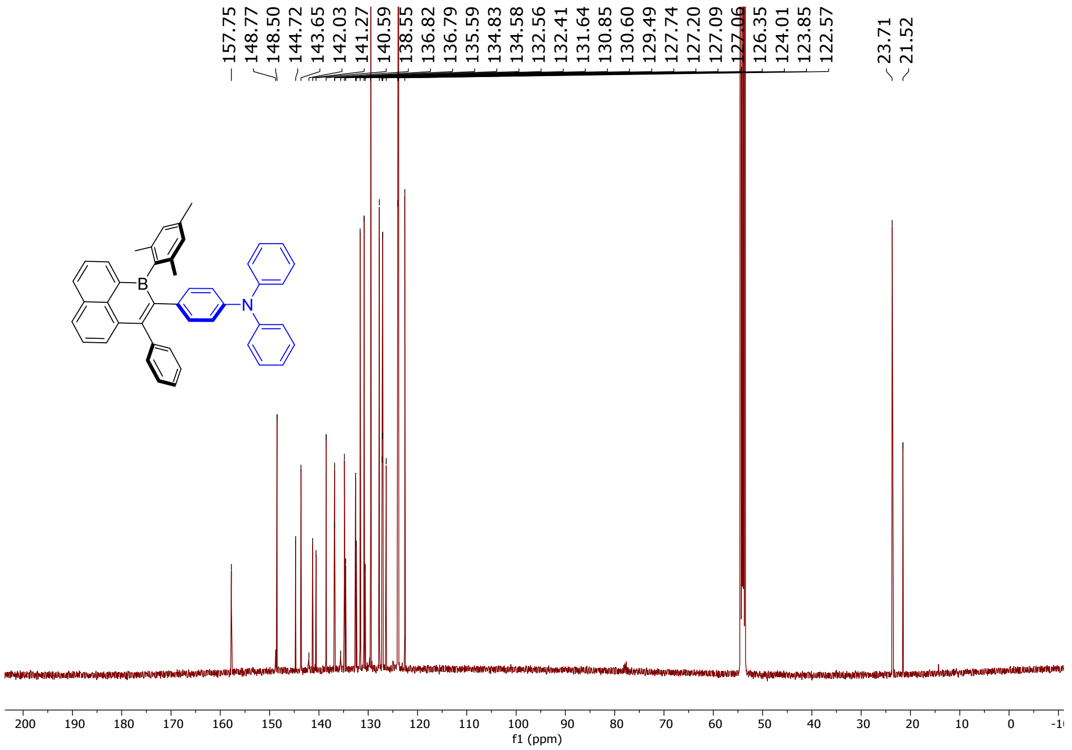

Figure **S23**. $^{13}C\{^{1}H\}$ NMR spectrum of compound **4** in CD_2Cl_2 (126 MHz).

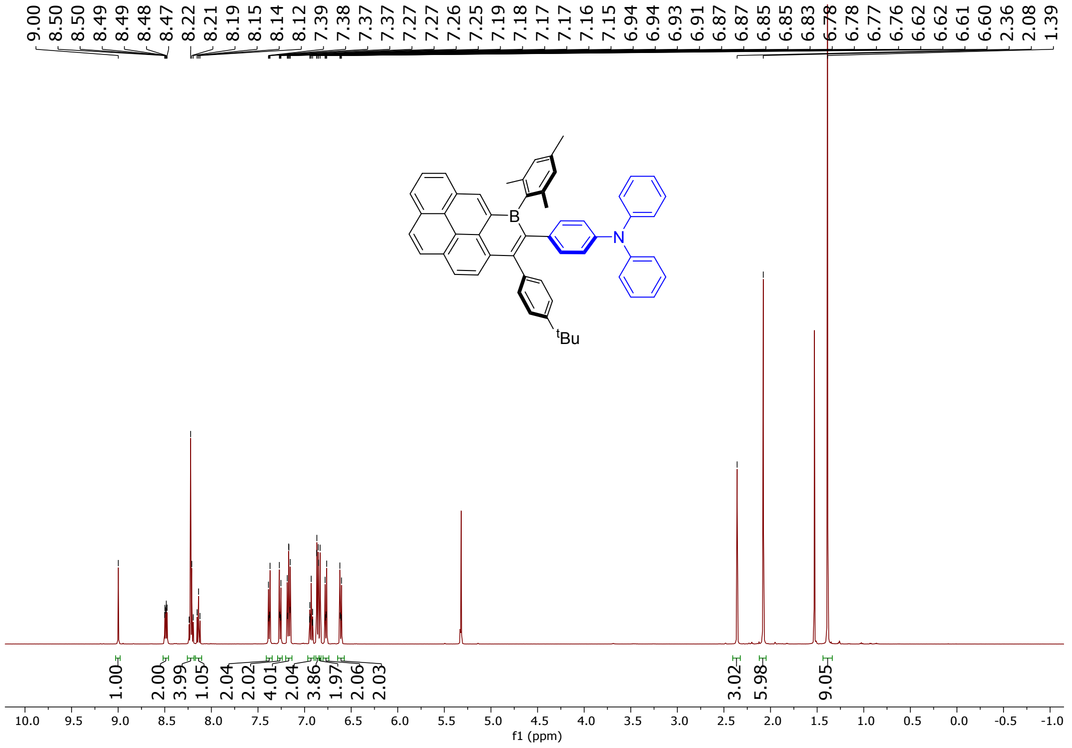

Figure **S24**. ^{1}H NMR spectrum of compound **5** in CD_2Cl_2 (500 MHz).

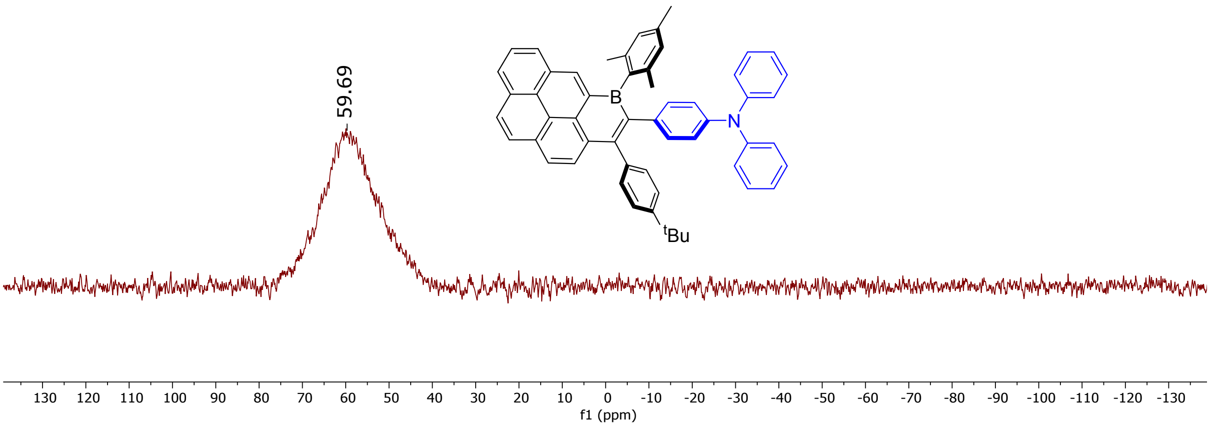

Figure **S25**. ^{11}B NMR spectrum of compound **5** in CD_2Cl_2 (160 MHz).

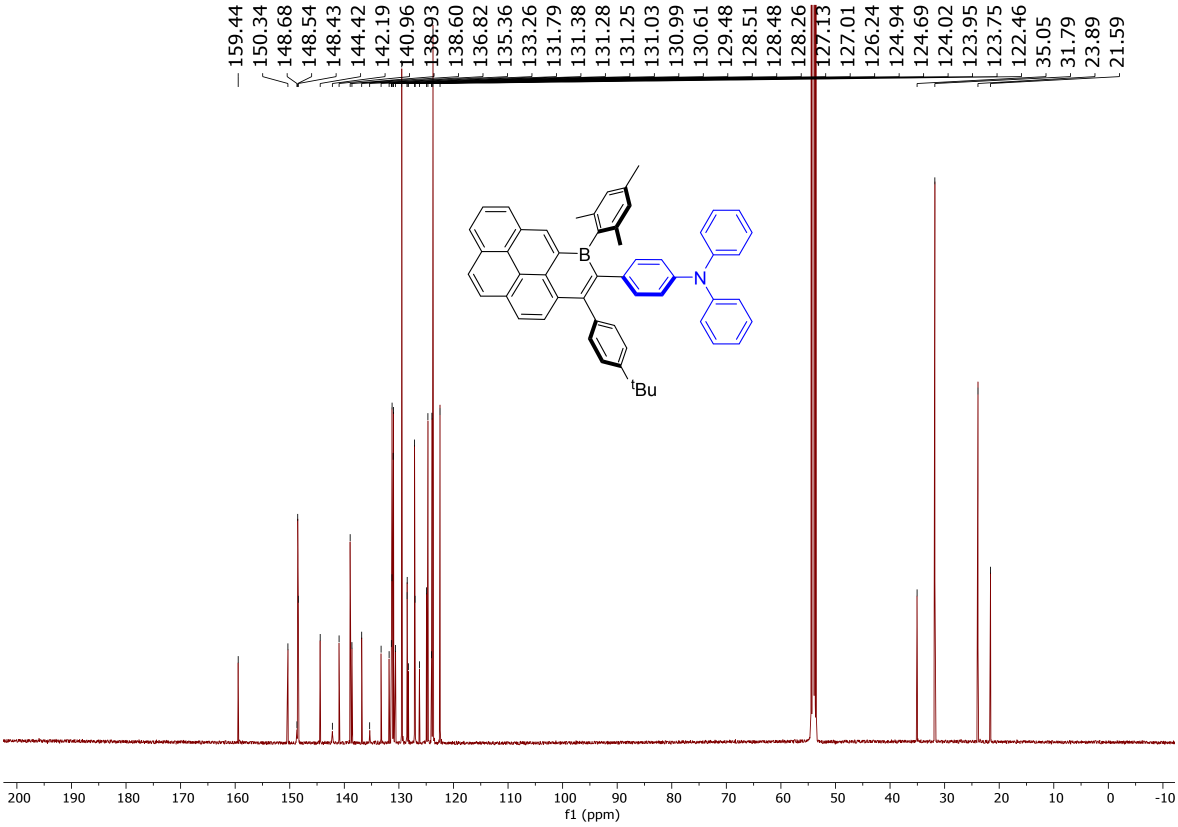

Figure **S26**. $^{13}C\{^{1}H\}$ NMR spectrum of compound **5** in CD_2Cl_2 (126 MHz).

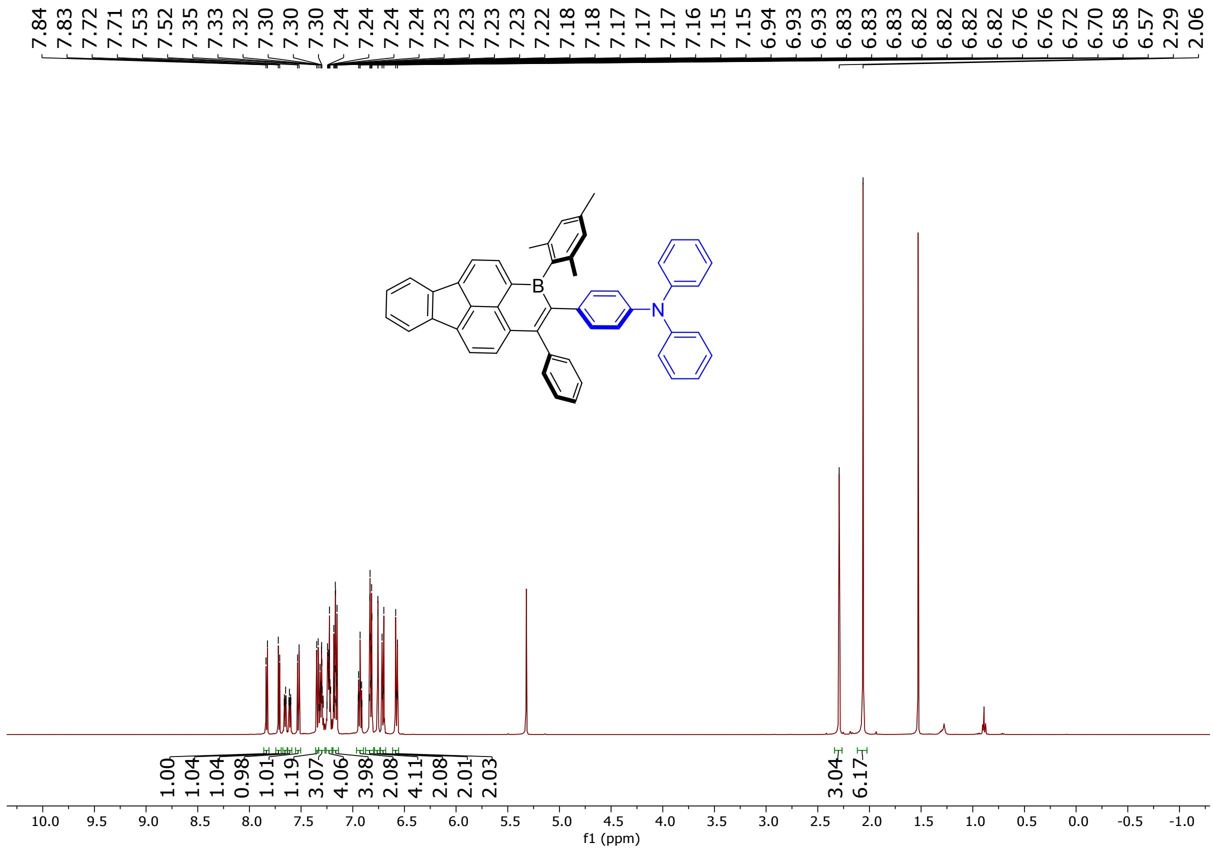

Figure **S27**. ^{1}H NMR spectrum of compound **6** in CD_2Cl_2 (500 MHz).

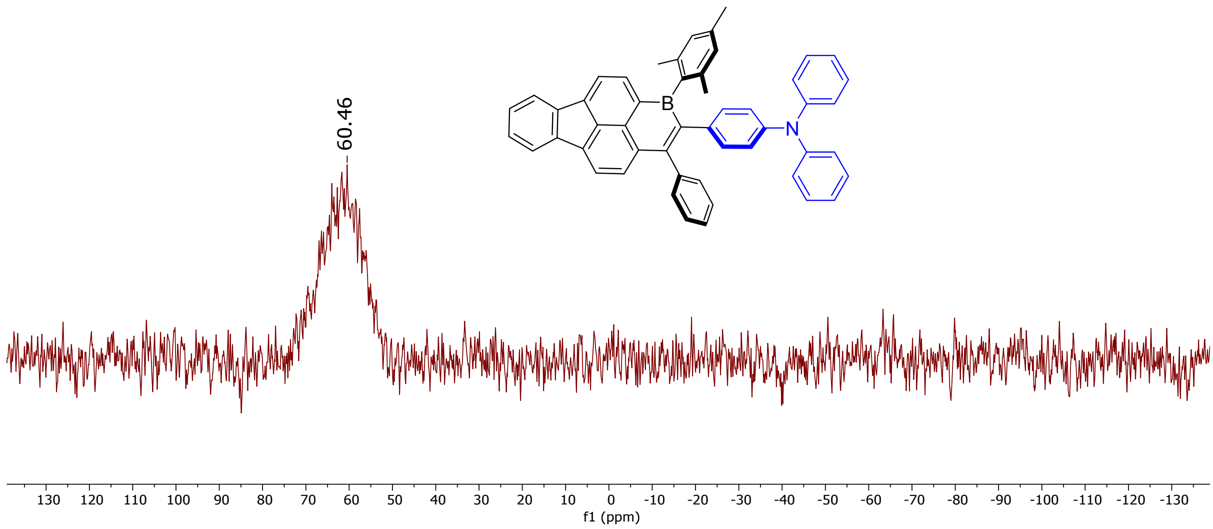

Figure **S28**. ^{11}B NMR spectrum of compound **6** in CD_2Cl_2 (128 MHz).

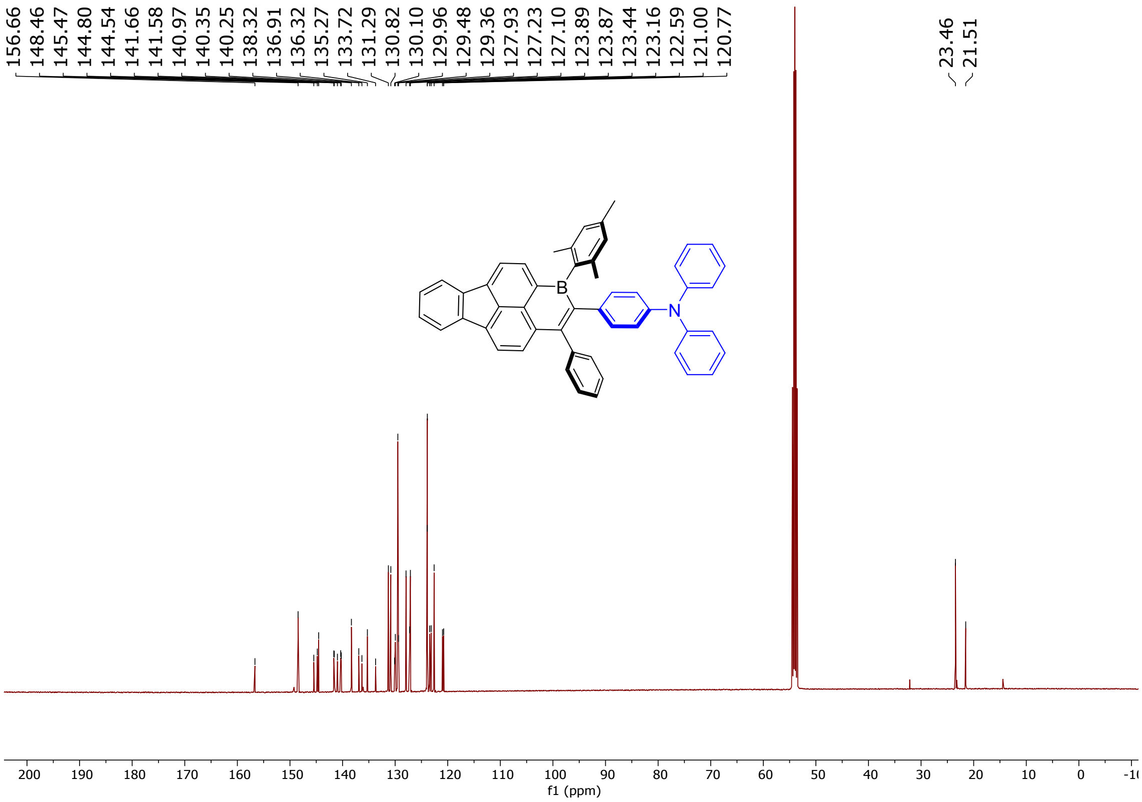

Figure **S29**. $^{13}C\{^{1}H\}$ NMR spectrum of compound **6** in CD_2Cl_2 (126 MHz).

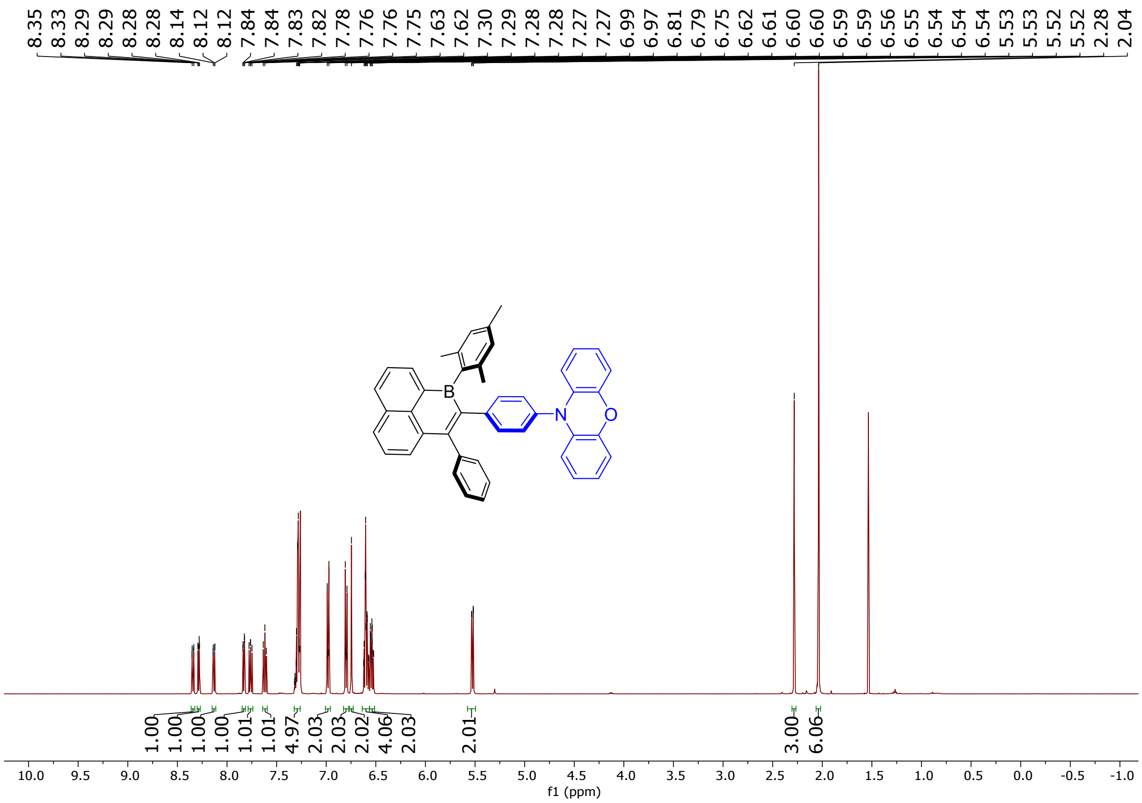

Figure **S30**. ^{1}H NMR spectrum of compound **7** in $CDCl_3$ (500 MHz).

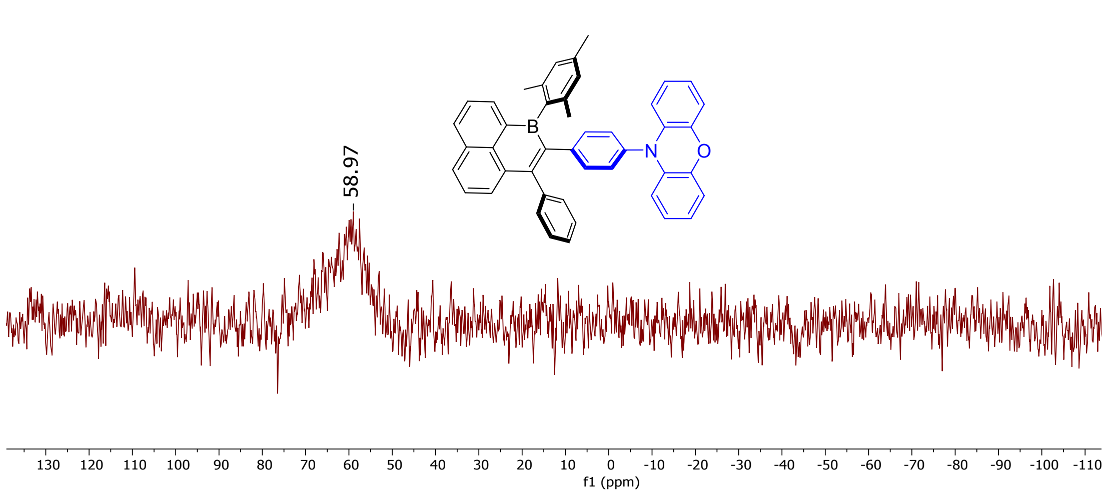

Figure **S31**. ^{11}B NMR spectrum of compound **7** in $CDCl_3$ (128 MHz).

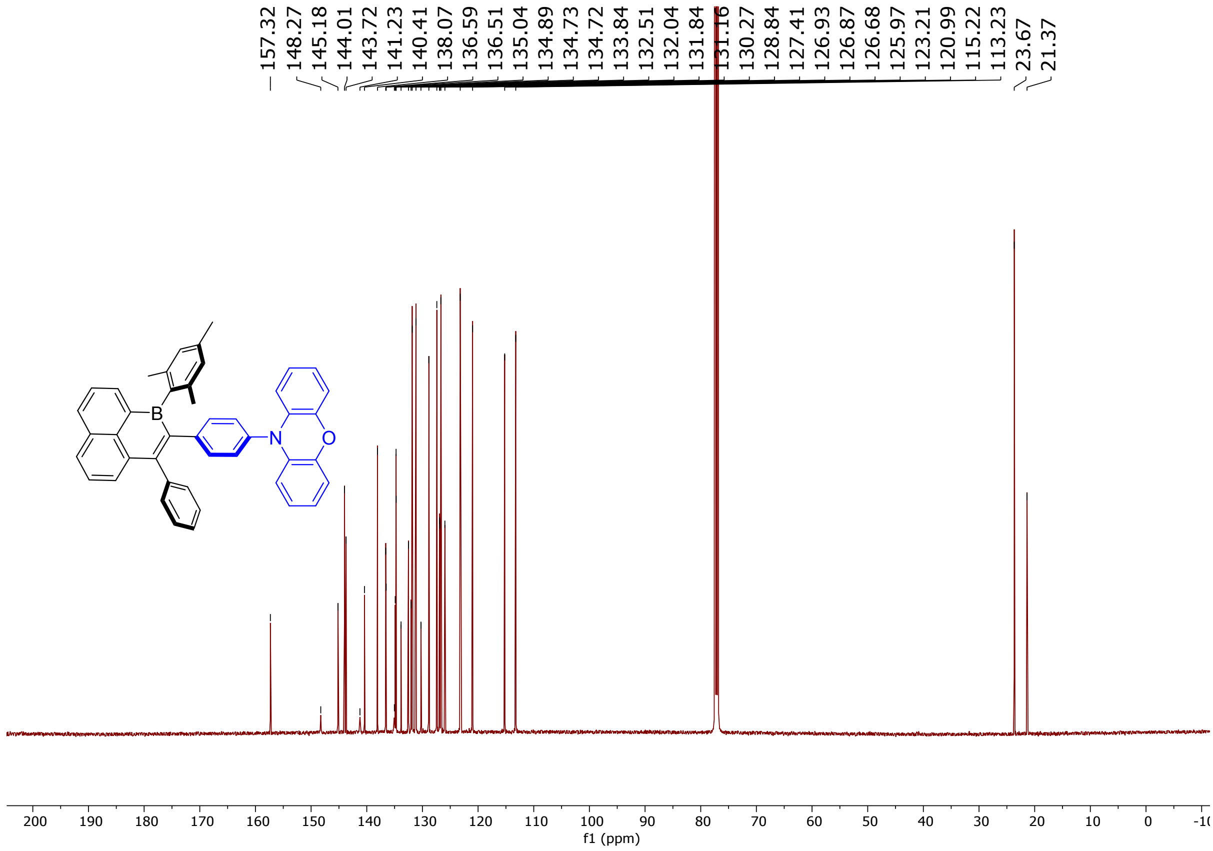

Figure **S32**. $^{13}C\{^{1}H\}$ NMR spectrum of compound **7** in $CDCl_3$ (126 MHz).

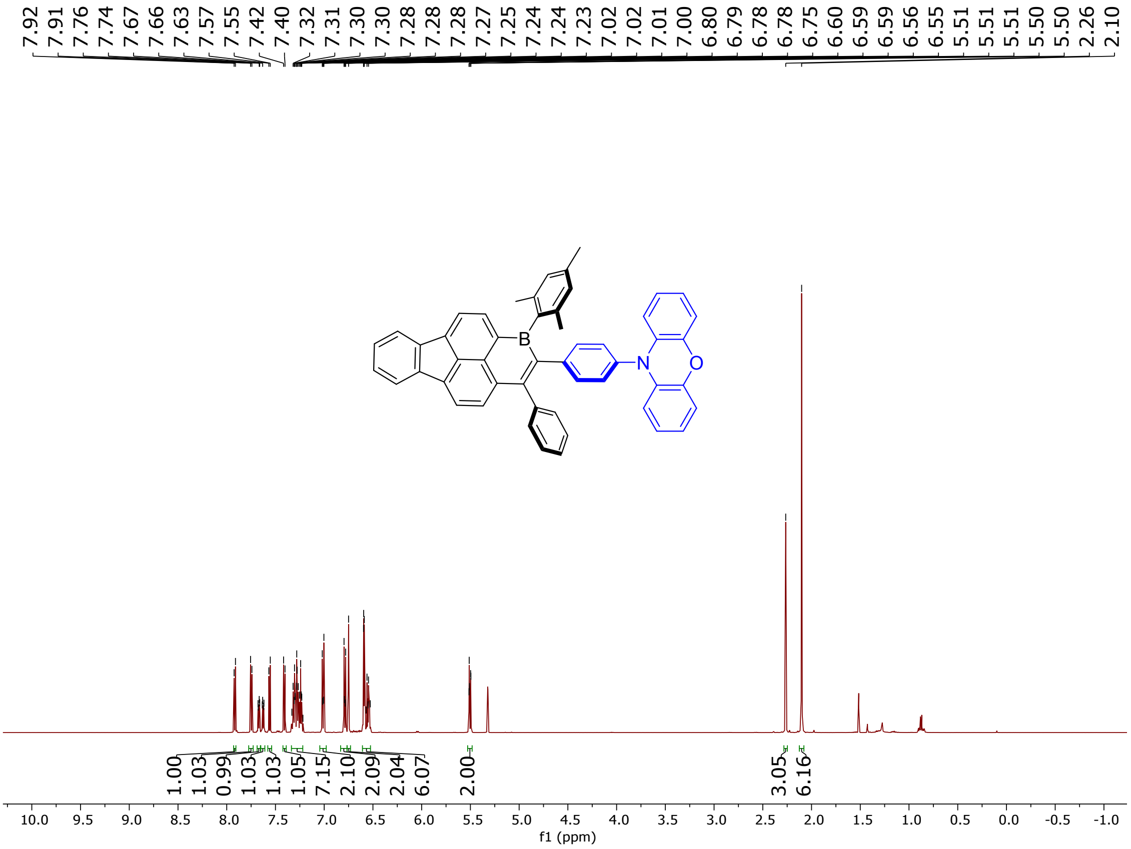

Figure **S33**. ^{1}H NMR spectrum of compound **8** in CD_2Cl_2 (500 MHz).

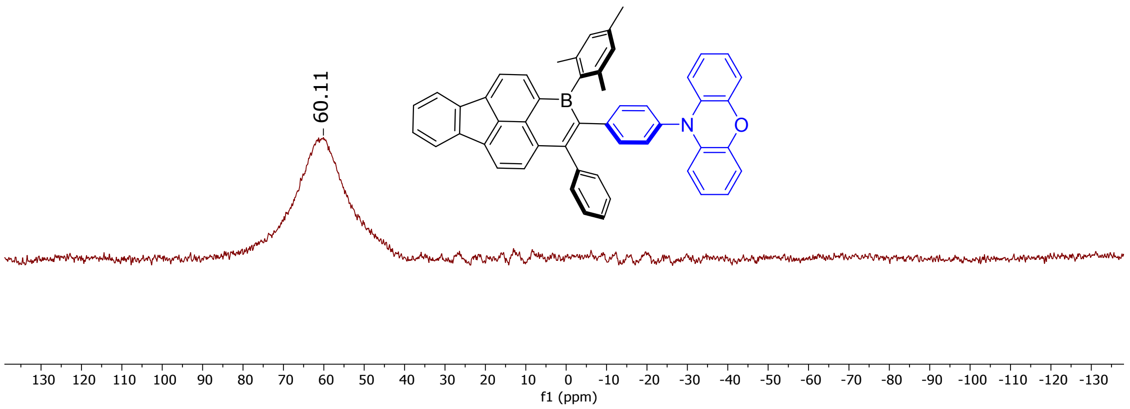

Figure **S34**. ^{11}B NMR spectrum of compound **8** in CD_2Cl_2 (160 MHz).

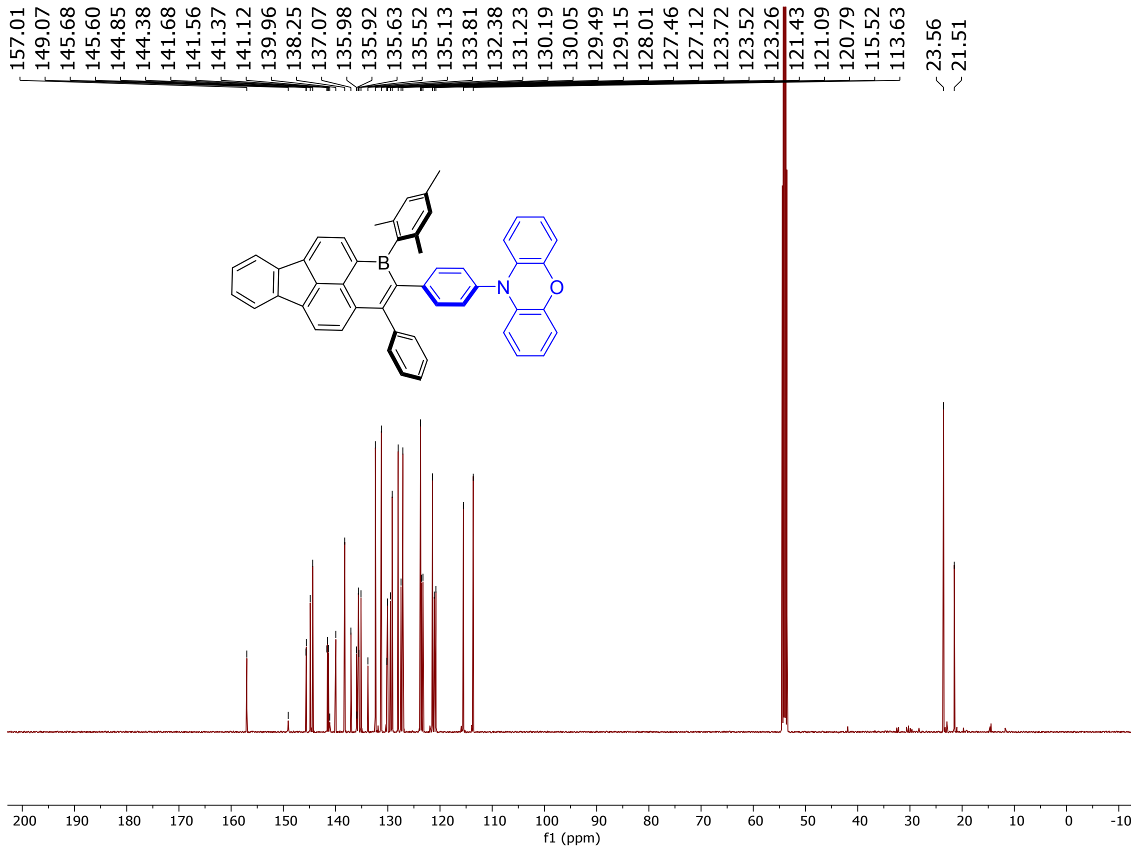

Figure **S35**. $^{13}C\{^{1}H\}$ NMR spectrum of compound **8** in CD_2Cl_2 (126 MHz).

S4. Crystal Structures

Crystallographic data were recorded on a Xcalibur, Eos diffractometer with Mo Kα radiation (mirror monochromator, λ=0.7107) at 120 K. The structure was solved with the ShelXT[5] solution program using dual methods and by using Olex2[6] as the graphical interface. The model was refined with ShelXL[7] using full matrix least squares minimisation on $\boldsymbol{F}^2$.Single crystals were obtained by slow evaporation of corresponding solutions (compound **5** in hexane/dichloromethane mixed solvents; compound **7** in hexane/ethyl acetate mixed solvents).

Selected crystallographic data are presented in the following table and full details in cif format can be obtained free of charge from the Cambridge Crystallographic Data Centre via www.ccdc.cam.uk/data_request/cif.

CCDC No	2265860	2265859
Empirical formula	$C_{56}H_{48}BCl_2N$	$C_{45}H_{34}BNO$
Formula weight	816.66	615.54
Temperature/K	120.01(11)	120.00(14)
Crystal system	monoclinic	monoclinic
Space group	$P2_1/n$	P21/c
a/Å	17.0991(4)	16.8494(2)
b/Å	9.8816(2)	12.0533(2)
c/Å	25.9164(5)	16.7996(2)
α/°	90	90
β/°	99.387(2)	102.3330(10)
γ/°	90	90
Volume/Å^3	4320.36(16)	3333.11(8)
Z	4	4
ρ_{calc}g/cm^3	1.256	1.227
μ/mm^{-1}	0.190	0.072
F(000)	1720.0	1296.0
Crystal size/mm^3	0.241 × 0.181 × 0.154	0.427 × 0.315 × 0.168
Radiation	Mo Kα (λ = 0.71073)	Mo Kα (λ = 0.71073)
2Θ range for data collection/°	6.736 to 58.494	6.76 to 58.9
Index ranges	-22 ≤ h ≤ 21, -13 ≤ k ≤ 13, -34 ≤ l ≤ 35	-21 ≤ h ≤ 23, -16 ≤ k ≤ 16, -21 ≤ l ≤ 22
Reflections collected	93192	71726
Independent reflections	10846 [R_{int} = 0.0523, R_{sigma} = 0.0362]	8491 [Rint = 0.0328, Rsigma = 0.0251]
Data/restraints/parameters	10846/0/547	8491/0/436
Goodness-of-fit on F^2	1.033	1.035
Final R indexes [I>=2σ (I)]	R_1 = 0.0494, wR_2 = 0.1200	R1 = 0.0473, wR2 = 0.1091
Final R indexes [all data]	R_1 = 0.0725, wR_2 = 0.1327	R1 = 0.0672, wR2 = 0.1198
Largest diff. peak/hole / e Å^{-3}	0.40/-0.58	0.29/-0.23

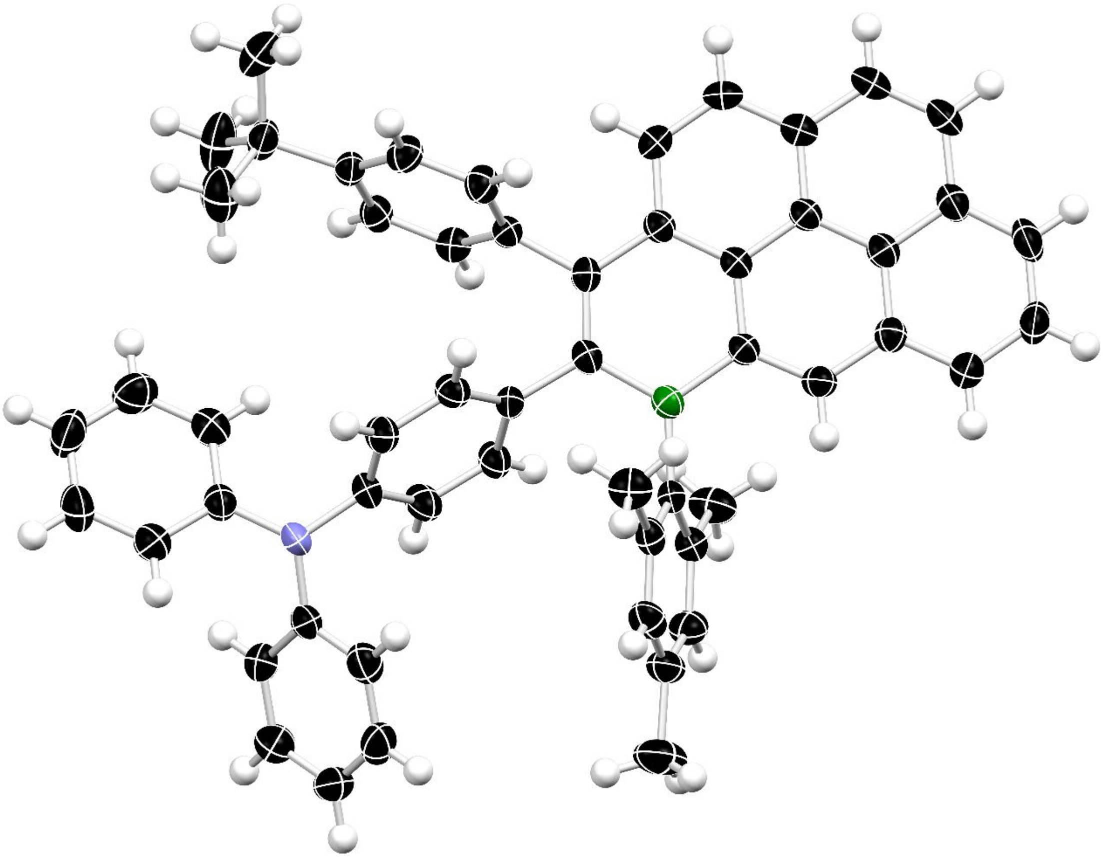

Figure **S36**. Thermal ellipsoid plot of compound **5** (70% probability). A solvent molecule (dichloromethane) was omitted for clarity.

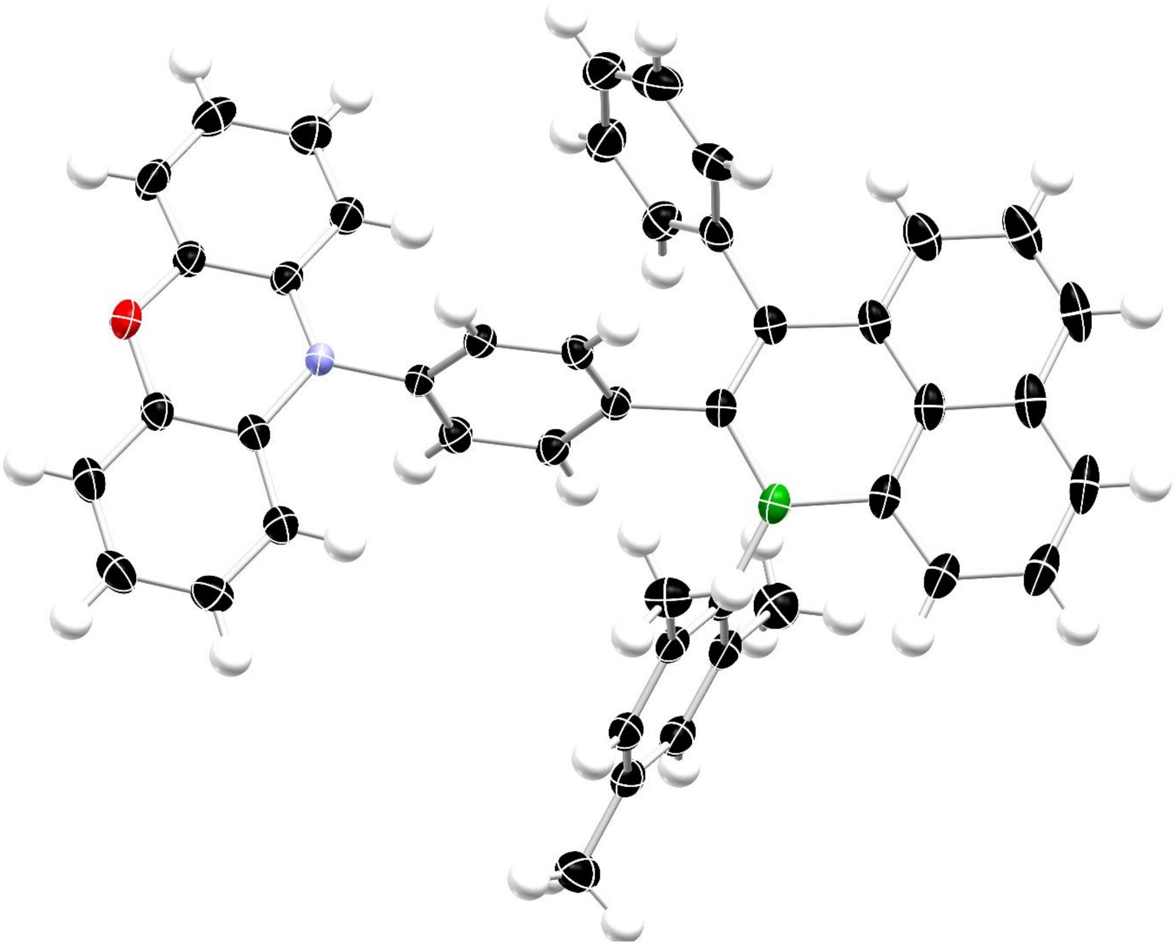

Figure **S37**. Thermal ellipsoid plot of compound **7** (70% probability).

S5. DFT Calculations

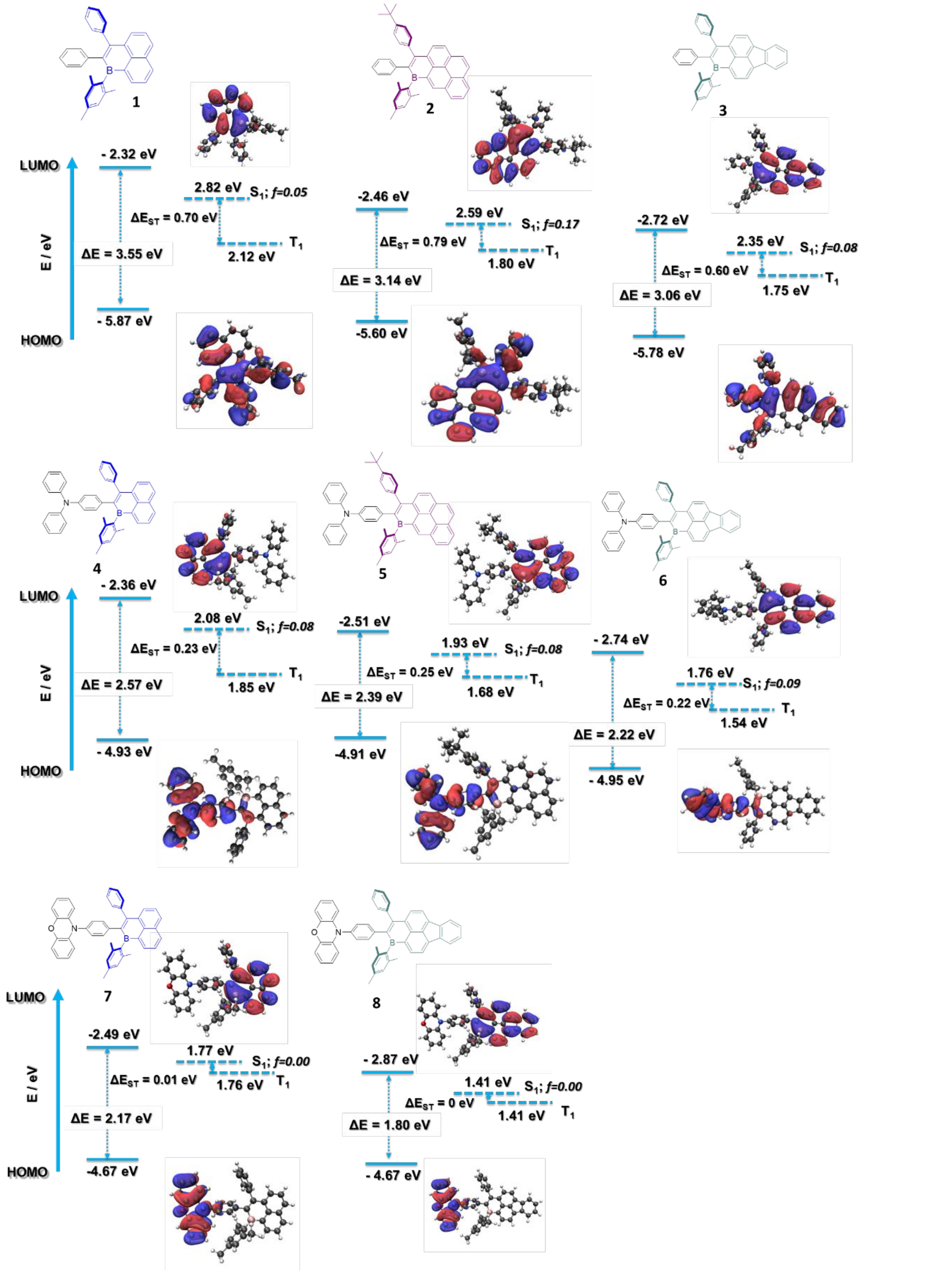

Figure **S38**. Theoretical modelling of the energies of the HOMO/LUMO orbitals and the S_1 and T_1 states and oscillator strength of compounds **1-8** and the electron density distribution (ISO value = 0.02) of the frontier molecular orbitals.

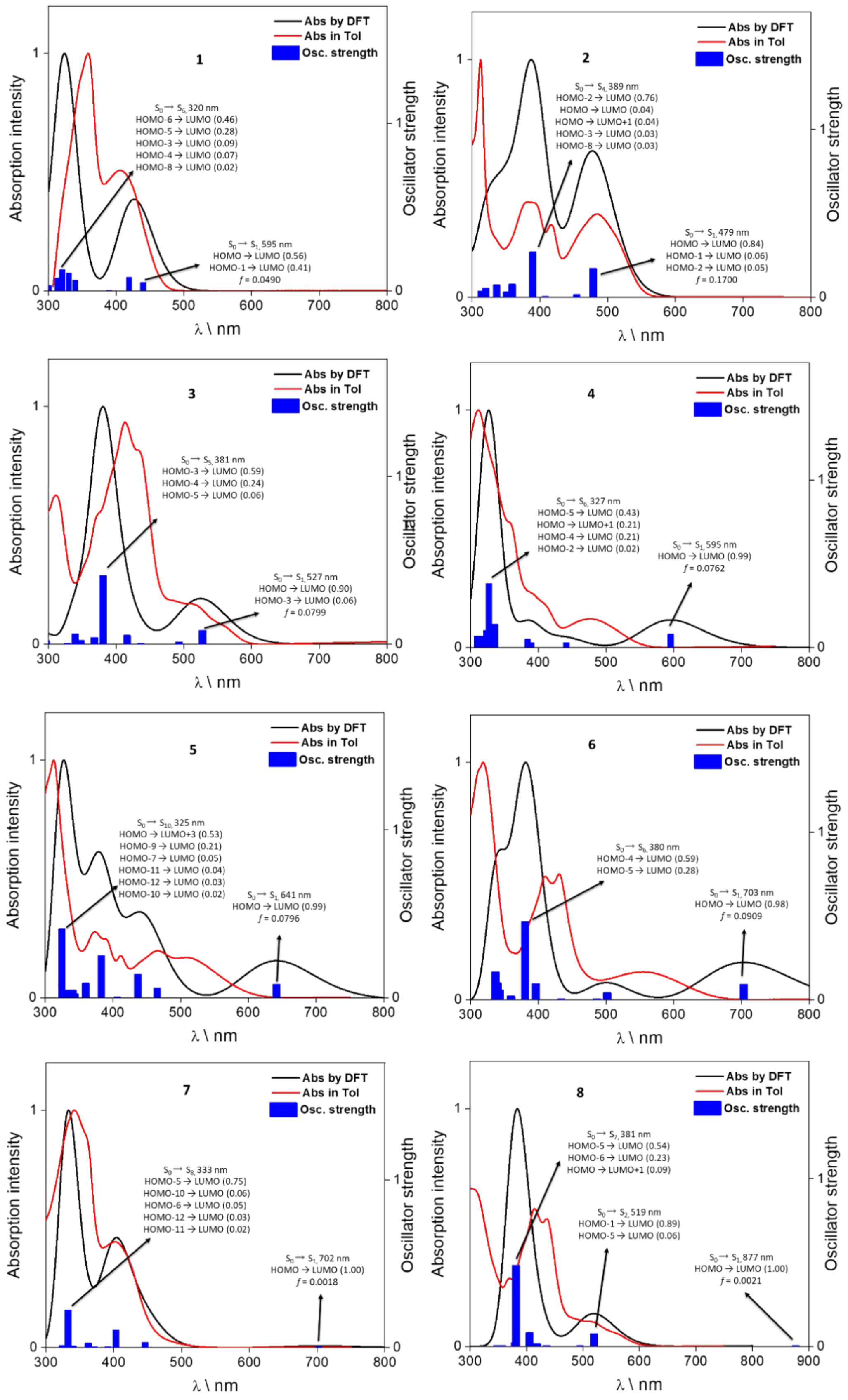

Figure **S39**. TDA-DFT simulation and experimental UV-Vis spectra of compounds **1-8** (fitted FWHM = 3000 cm^{-1}).

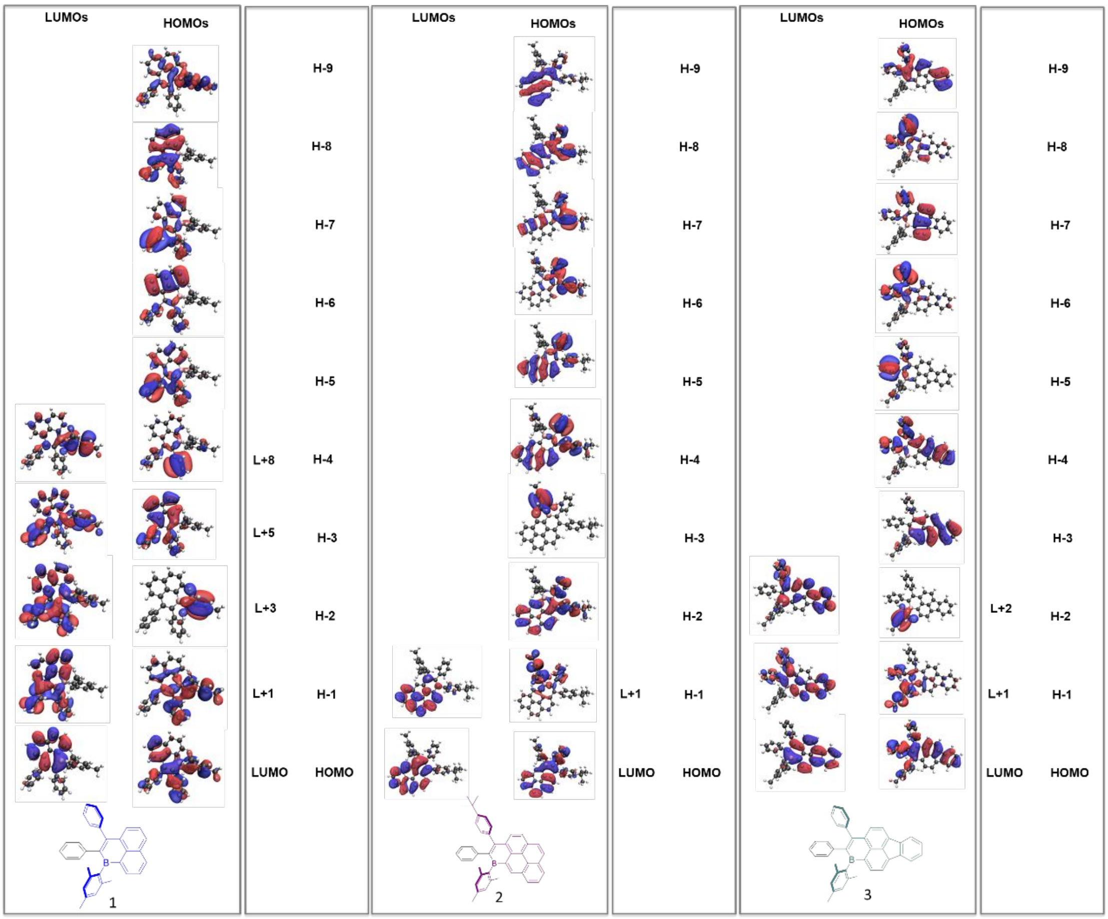

Figure **S40**. Calculated distribution of molecular orbitals for **1**, **2** and **3**. (isovalue= 0.02).

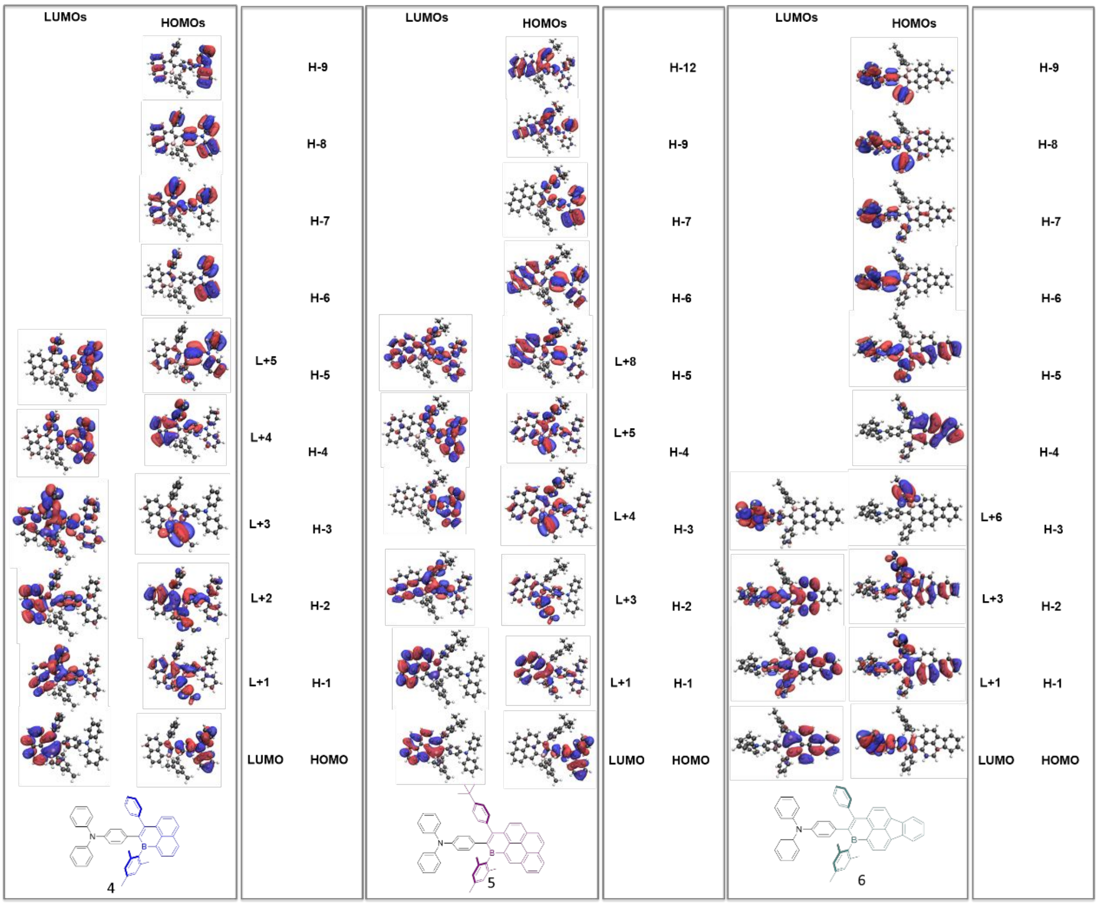

Figure **S41**. Calculated distribution of molecular orbitals for **4**, **5** and **6**. (isovalue= 0.02).

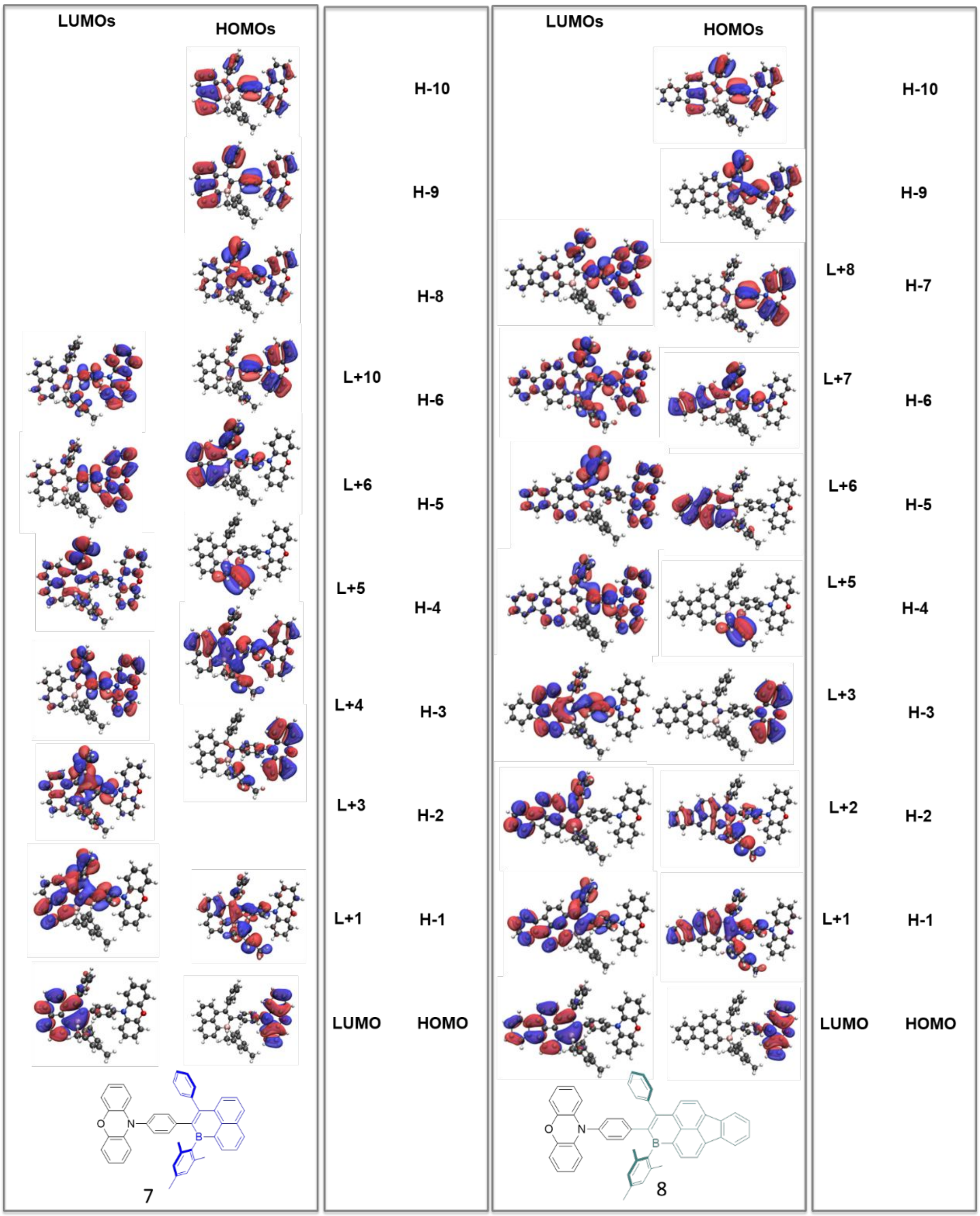

Figure **S42**. Calculated distribution of molecular orbitals for **7** and **8**. (isovalue= 0.02).

S6. Electrochemical Properties

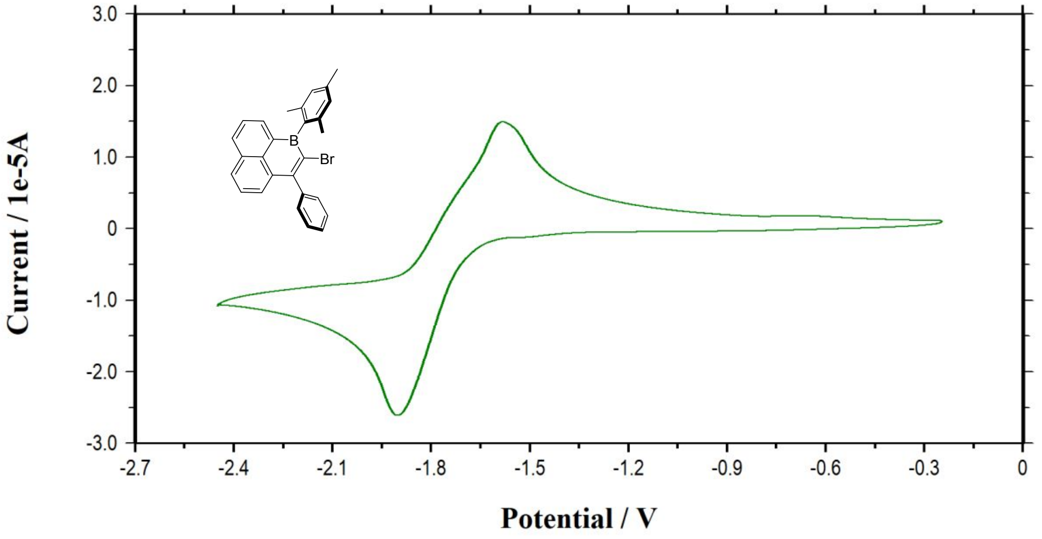

Figure **S43**. The Cyclic Voltammetry diagram of **BPAH1**. Measured in THF (1 mM), with [^{n}Bu$_4$N][PF$_6$] (0.1 M) as the supporting electrolyte at a scan rate of 100 mV/s at room temperature (Initial Potential: 0 V; Initial Scan Polarity: Negative). The data are reported in the IUPAC convention.

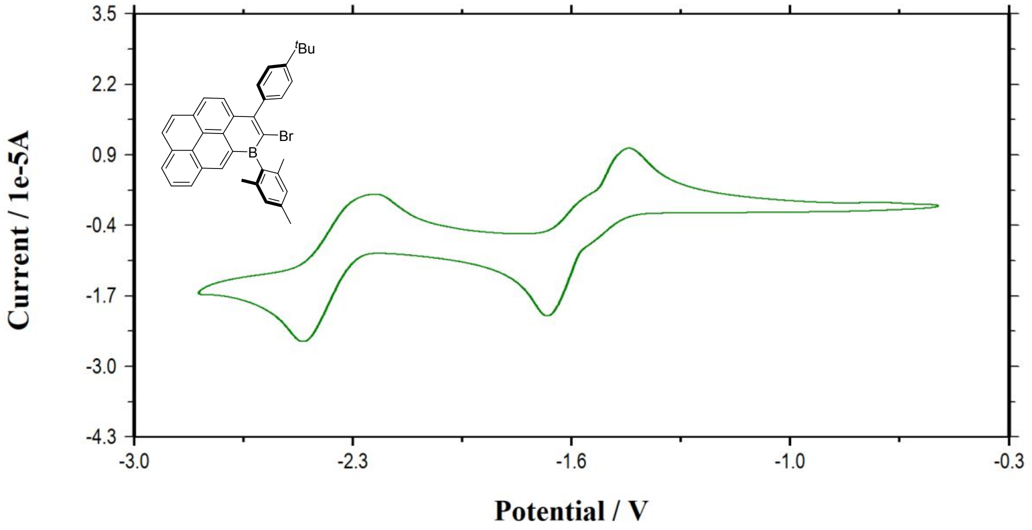

Figure **S44**. The Cyclic Voltammetry diagram of **BPAH2**. Measured in THF (1 mM), with [^{n}Bu$_4$N][PF$_6$] (0.1 M) as the supporting electrolyte at a scan rate of 100 mV/s at room temperature(Initial Potential: 0 V; Initial Scan Polarity: Positive). The data are reported in the IUPAC convention.

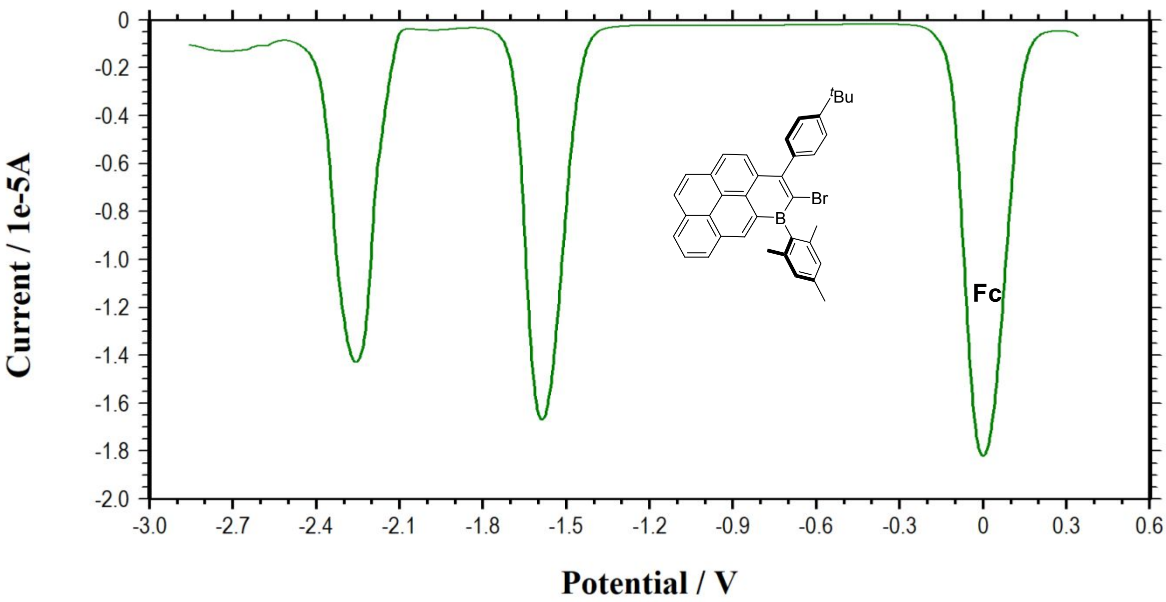

Figure **S45**. The differential pulse voltammetry diagram of **BPAH2**. Measured in THF (1 mM), with [^{n}Bu$_4$N][PF$_6$] (0.1 M) as the supporting electrolyte and ferrocene as the internal standard at room temperature. Incr E : 0.004 V, amplitude: 0.05 V, pulse width: 0.06 s, sampling width: 0.02 s, pulse period. The data are reported in the IUPAC convention.

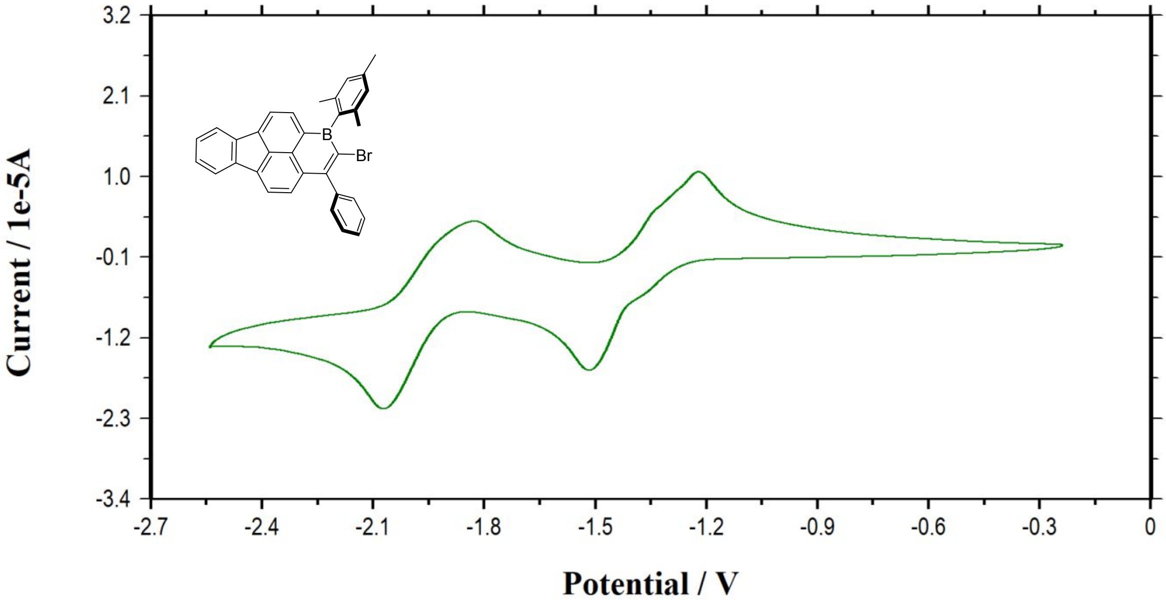

Figure **S46**. The Cyclic Voltammetry diagram of **BPAH3**. Measured in THF (1 mM), with [^{n}Bu$_4$N][PF$_6$] (0.1 M) as the supporting electrolyte at a scan rate of 100 mV/s at room temperature (Initial Potential: 0 V; Initial Scan Polarity: Negative). The data are reported in the IUPAC convention.

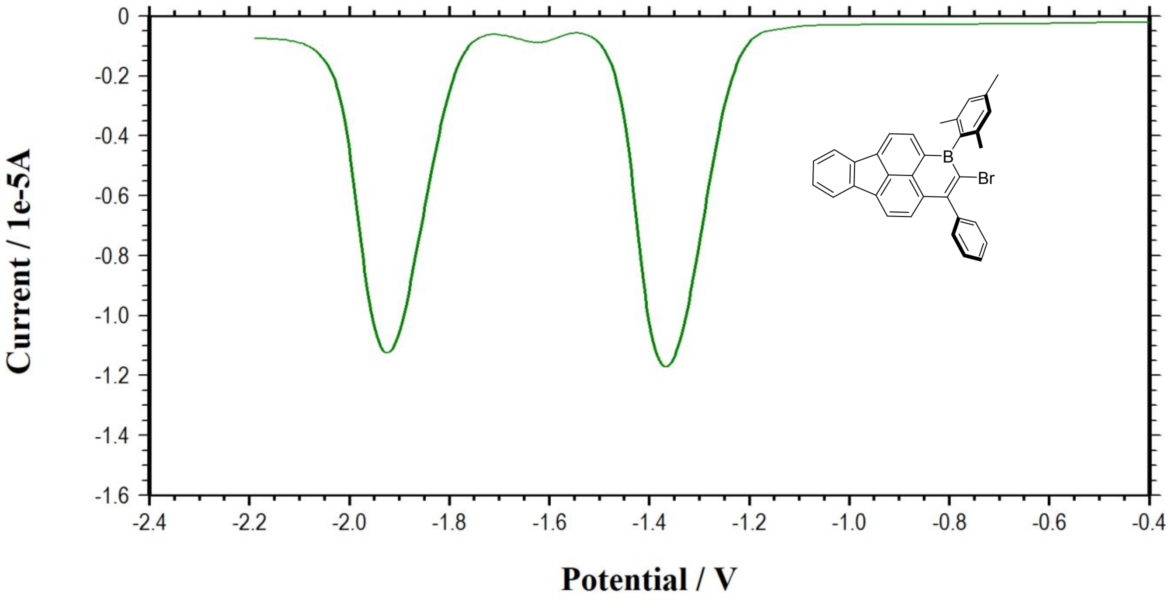

Figure **S47**. The differential pulse voltammetry diagram of **BPAH3**. Measured in THF (1 mM), with [$^{n}Bu_4N$][PF_6] (0.1 M) as the supporting electrolyte at room temperature. Incr E : 0.004 V, amplitude: 0.05 V, pulse width: 0.06 s, sampling width: 0.02 s, pulse period. The data are reported in the IUPAC convention

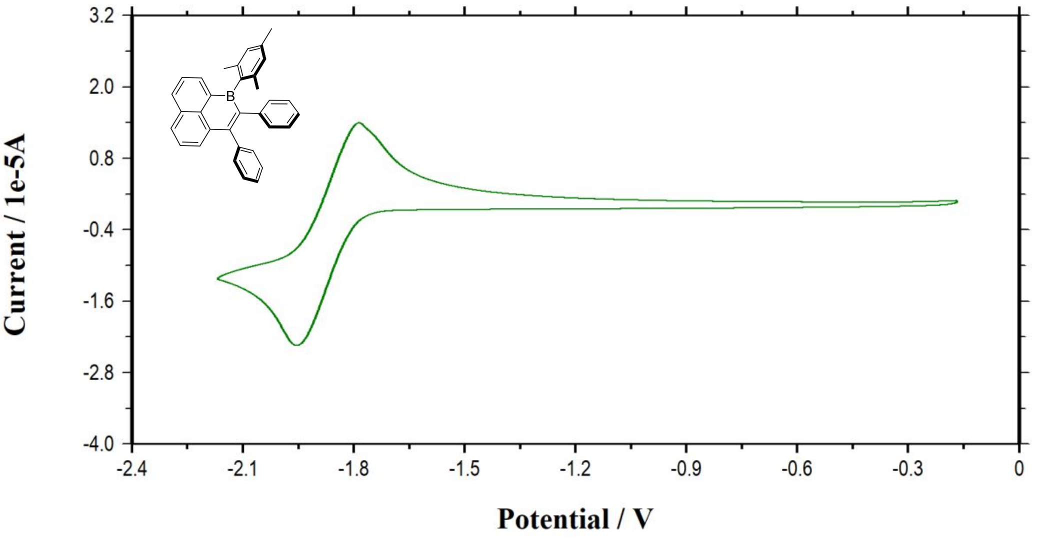

Figure **S48**. The Cyclic Voltammetry diagram of compound **1**. Measured in THF (1 mM), with [$^{n}Bu_4N$][PF_6] (0.1 M) as the supporting electrolyte at a scan rate of 100 mV/s at room temperature (Initial Potential: 0 V; Initial Scan Polarity: Negative). The data are reported in the IUPAC convention.

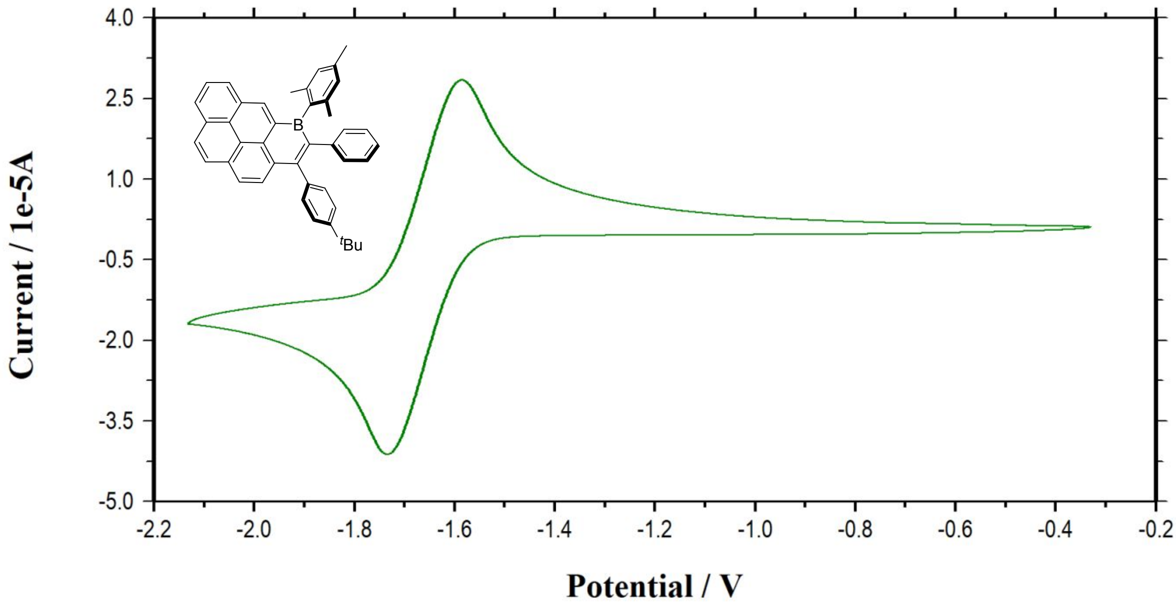

Figure **S49**. The Cyclic Voltammetry diagram of compound **2**. Measured in THF (1 mM), with [^{n}Bu$_4$N][PF$_6$] (0.1 M) as the supporting electrolyte at a scan rate of 100 mV/s at room temperature (Initial Potential: 0 V; Initial Scan Polarity: Negative). The data are reported in the IUPAC convention.

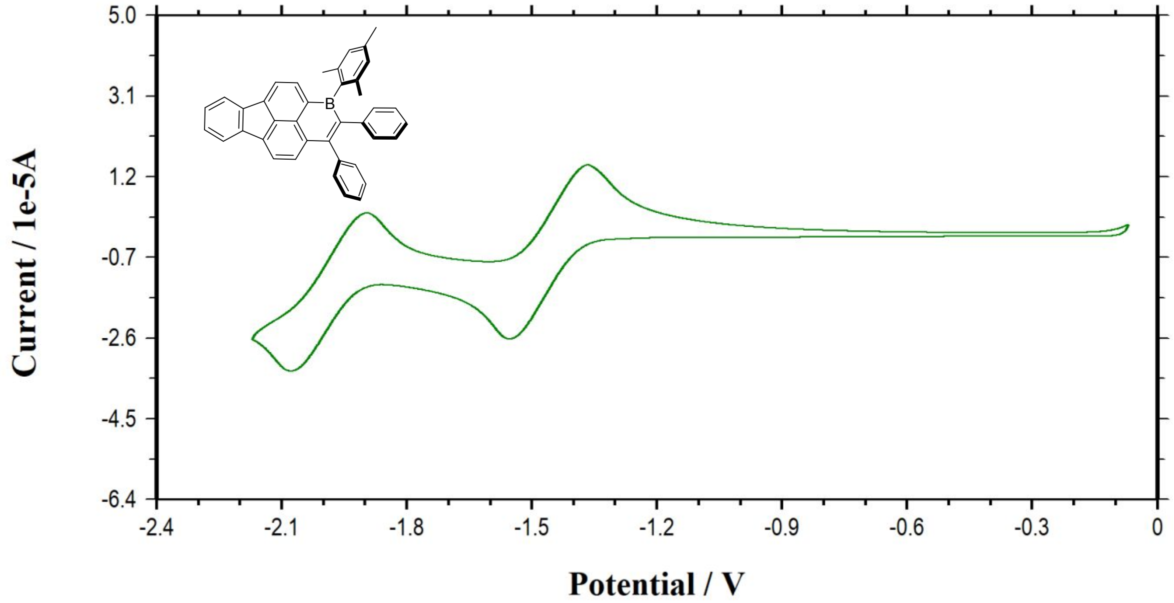

Figure **S50**. The Cyclic Voltammetry diagram of compound **3**. Measured in THF (1 mM), with [^{n}Bu$_4$N][PF$_6$] (0.1 M) as the supporting electrolyte at a scan rate of 100 mV/s at room temperature (Initial Potential: 0 V; Initial Scan Polarity: Negative). The data are reported in the IUPAC convention.

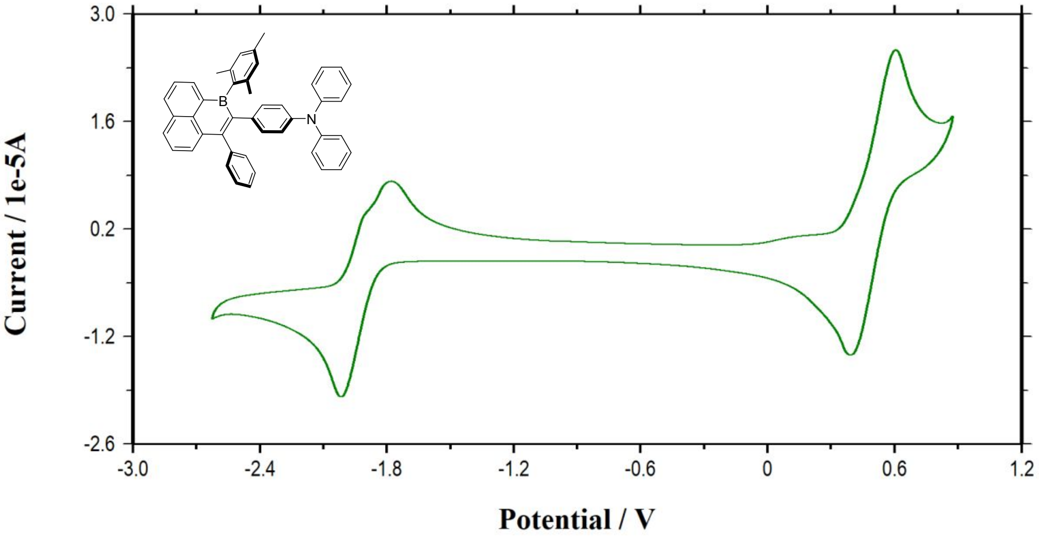

Figure **S51**. The Cyclic Voltammetry diagram of compound **4**. Measured in THF (1 mM), with [^{n}Bu$_4$N][PF$_6$] (0.1 M) as the supporting electrolyte at a scan rate of 100 mV/s at room temperature (Initial Potential: 0 V; Initial Scan Polarity: Positive). The data are reported in the IUPAC convention.

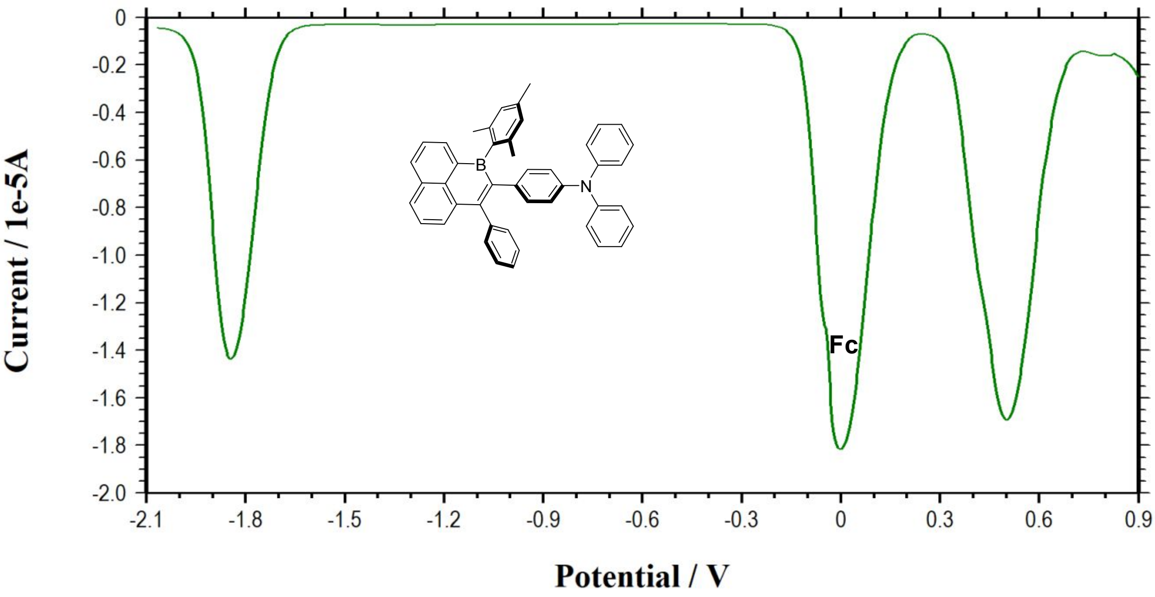

Figure **S52**. The differential pulse voltammetry diagram of compound **4**. Measured in THF (1 mM), with [^{n}Bu$_4$N][PF$_6$] (0.1 M) as the supporting electrolyte and ferrocene as the internal standard at room temperature. Incr E : 0.004 V, amplitude: 0.05 V, pulse width: 0.06 s, sampling width: 0.02 s, pulse period. The data are reported in the IUPAC convention.

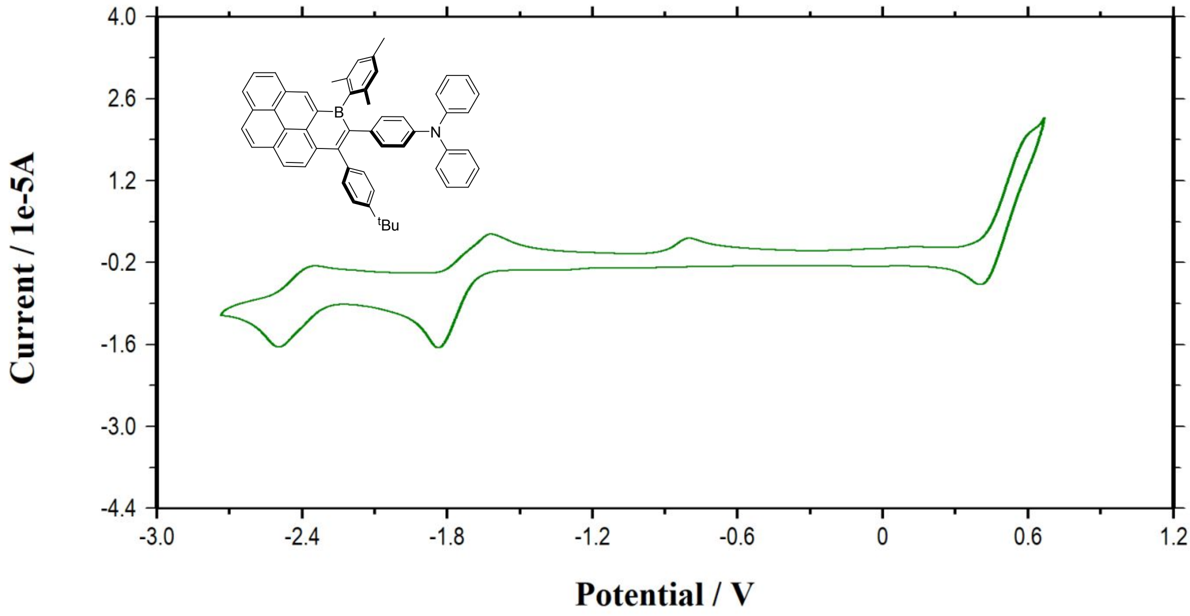

Figure **S53**. The Cyclic Voltammetry diagram of compound **5**. Measured in THF (1 mM), with [^{n}Bu$_4$N][PF$_6$] (0.1 M) as the supporting electrolyte at a scan rate of 100 mV/s at room temperature (Initial Potential: 0 V; Initial Scan Polarity: Negative). The data are reported in the IUPAC convention.

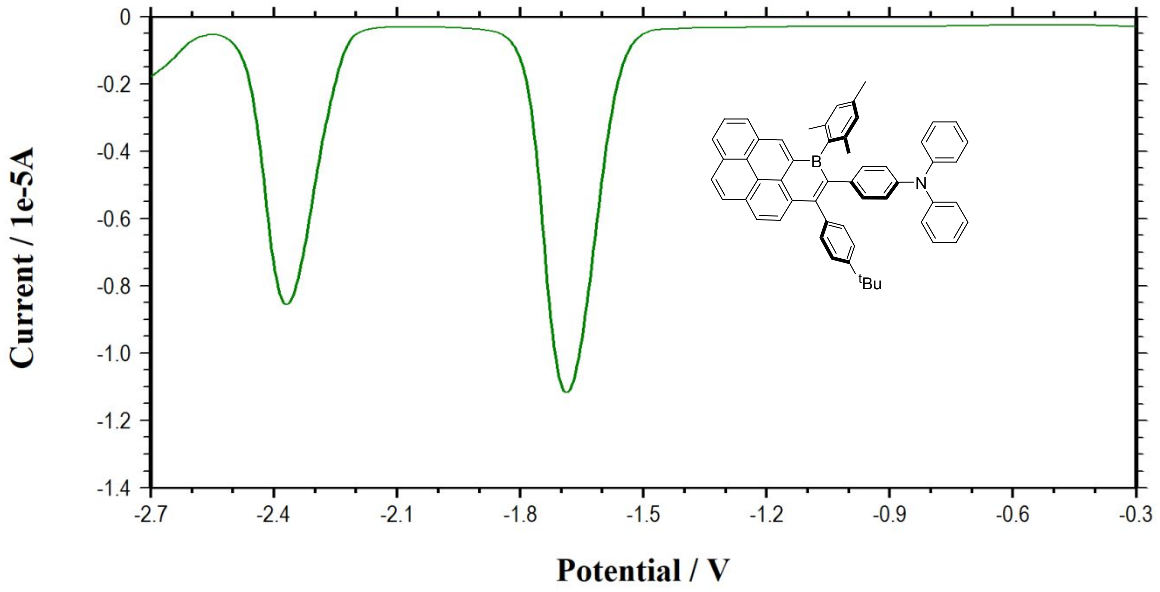

Figure **S54**. The differential pulse voltammetry diagram of compound **5**. Measured in THF (1 mM), with [^{n}Bu$_4$N][PF$_6$] (0.1 M) as the supporting electrolyte at room temperature. Incr E : 0.004 V, amplitude: 0.05 V, pulse width: 0.06 s, sampling width: 0.02 s, pulse period. The data are reported in the IUPAC convention.

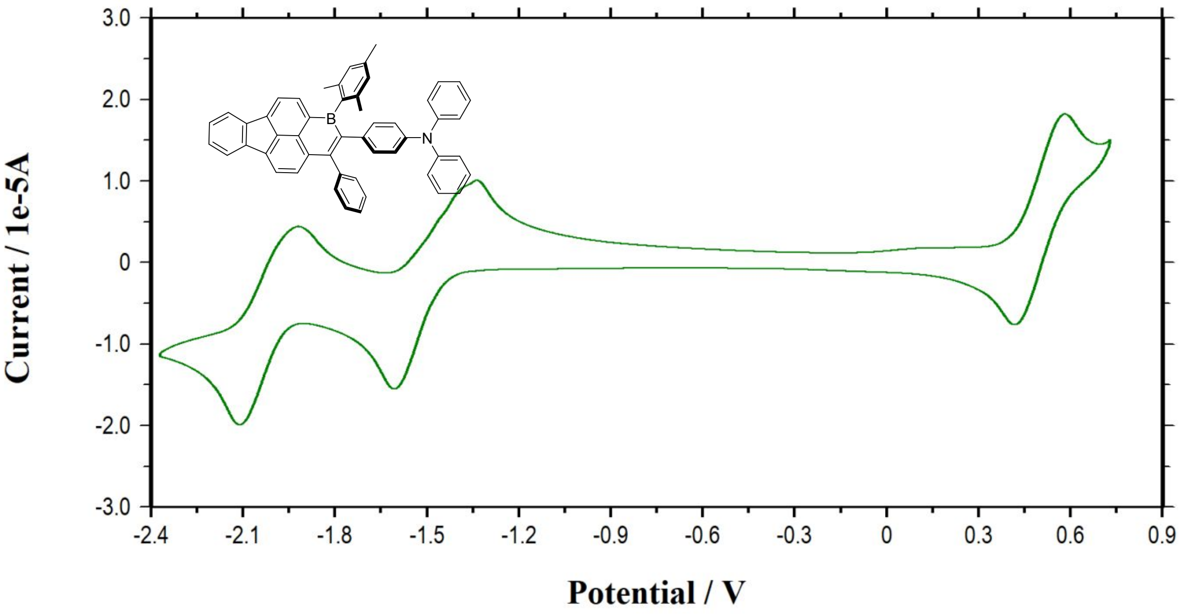

Figure **S55**. The Cyclic Voltammetry diagram of compound **6**. Measured in THF (1 mM), with [^{n}Bu$_4$N][PF$_6$] (0.1 M) as the supporting electrolyte at a scan rate of 100 mV/s at room temperature (Initial Potential: 0 V; Initial Scan Polarity: Negative). The data are reported in the IUPAC convention.

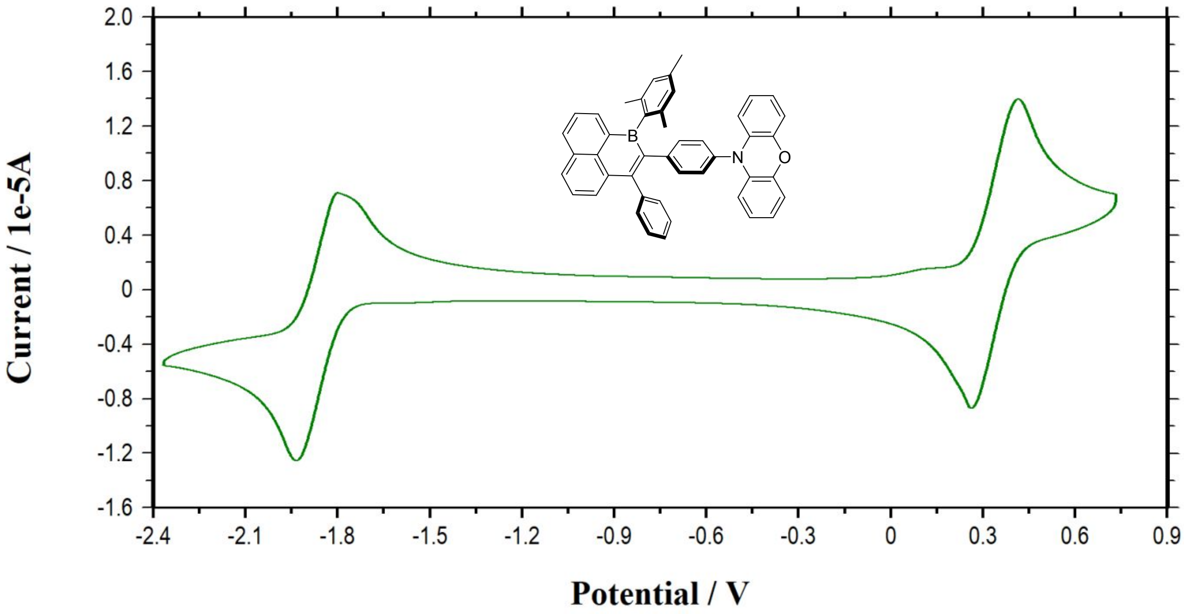

Figure **S56**. The Cyclic Voltammetry diagram of compound **7**. Measured in THF (1 mM), with [^{n}Bu$_4$N][PF$_6$] (0.1 M) as the supporting electrolyte at a scan rate of 100 mV/s at room temperature (Initial Potential: 0 V; Initial Scan Polarity: Negative). The data are reported in the IUPAC convention.

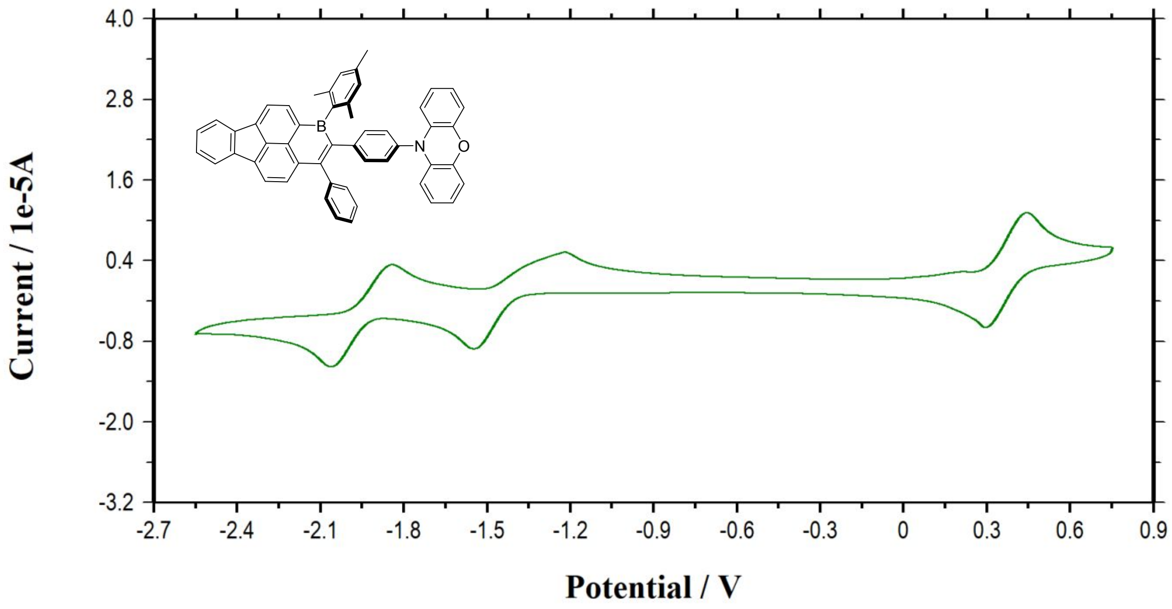

Figure **S57**. The Cyclic Voltammetry diagram of compound **8**. Measured in THF (1 mM), with [^{n}Bu$_4$N][PF$_6$] (0.1 M) as the supporting electrolyte at a scan rate of 100 mV/s at room temperature (Initial Potential: 0 V; Initial Scan Polarity: Negative). The data are reported in the IUPAC convention.

S6. Photophysical Properties

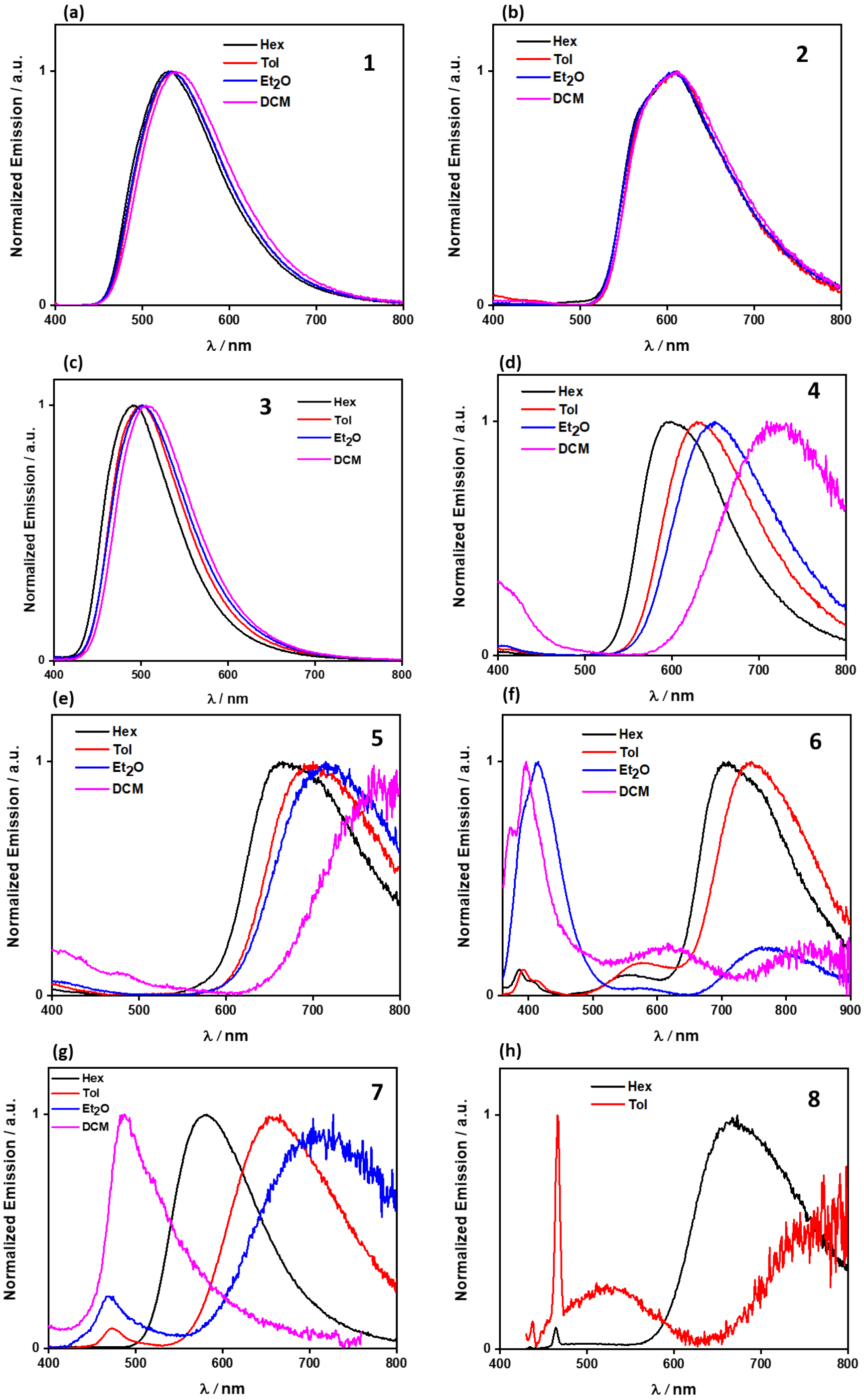

Figure **S58**. PL solvatochromism study of compounds **1-8.** (λ_{exc} = 330 nm).

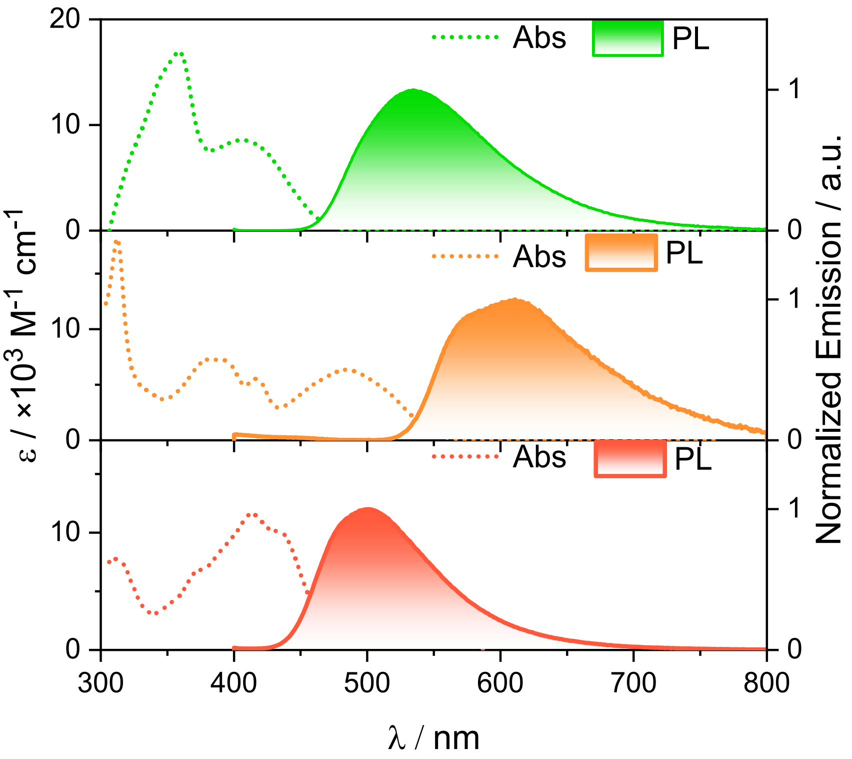

Figure **S59**. UV-vis absorption and PL spectra of **1-3** in toluene (λ_{exc} = 330 nm).

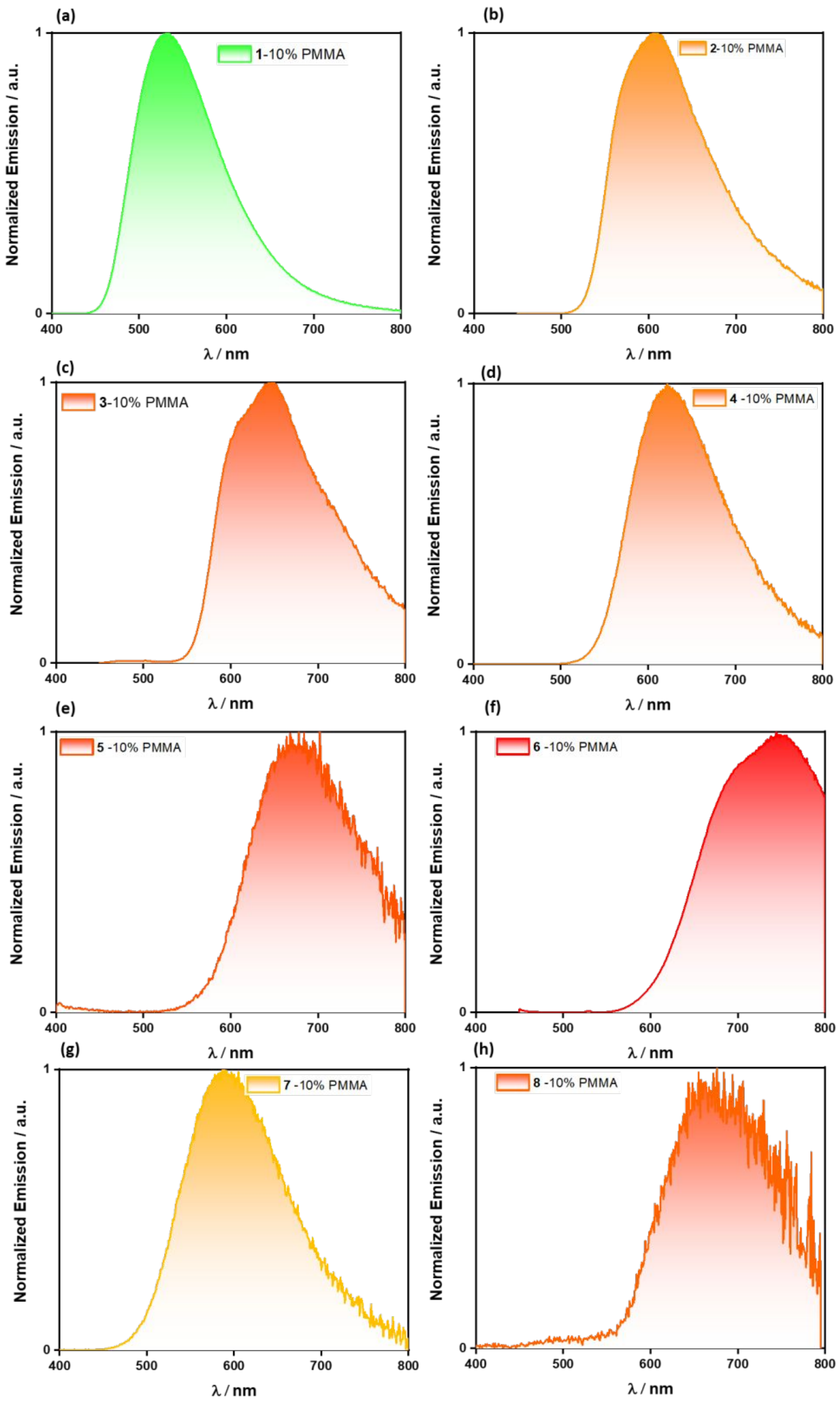

Figure **S60**. PL of compounds **1-8** 10 wt% doped films in PMMA (λ_{exc}= 330 nm).

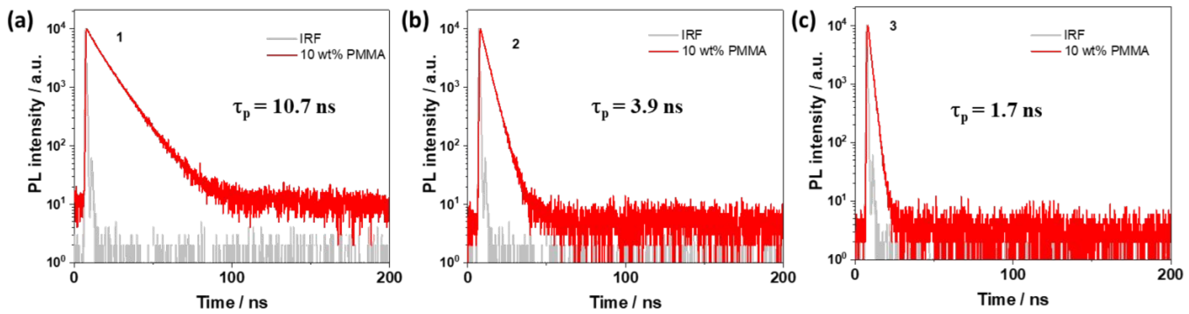

Figure **S61**. Time-resolved PL decay of 10 wt% doped films of (a) **1**, (b) **2** and (c) **3** in PMMA at room temperature (λ_{exc}= 379 nm).

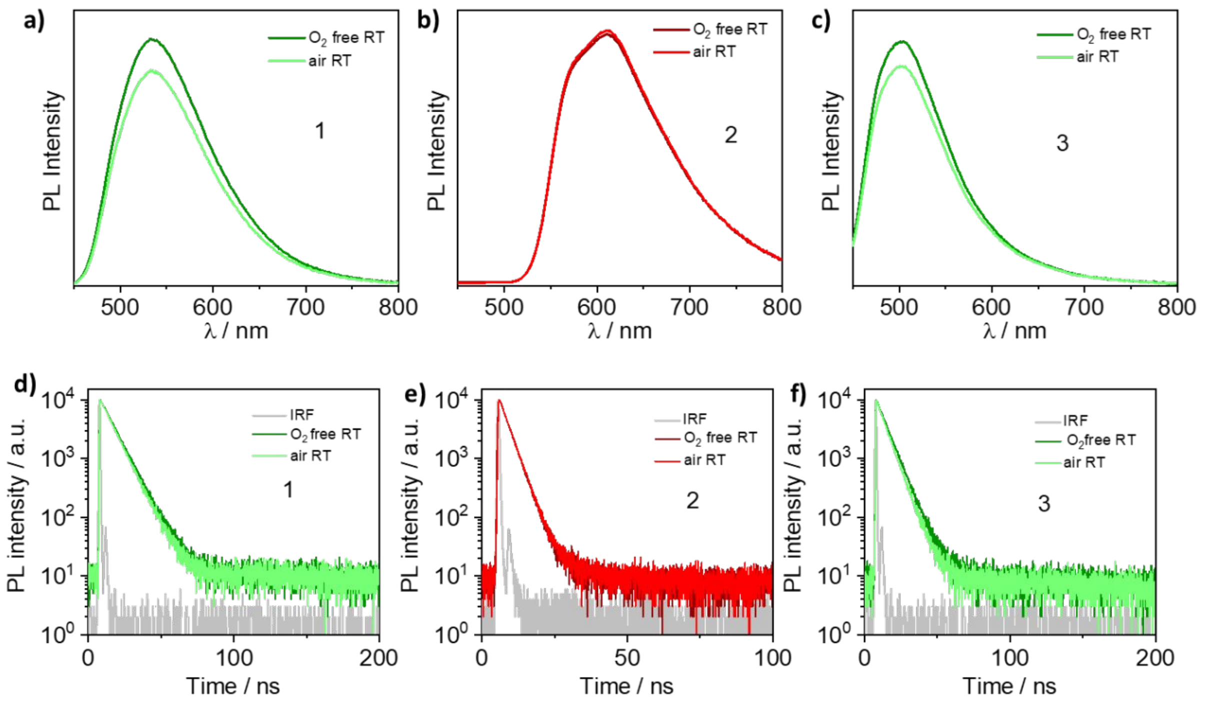

Figure **S62**. Steady-state PL spectra of 10^{-5} M (a) **1** (b) **2** and (c) **3** (λ_{exc} = 330 nm) and time-resolved PL decays of 10^{-5} M (d) **1** (e) **2** and (f) **3** (λ_{exc} = 379 nm) in toluene under aerated and degassed conditions.

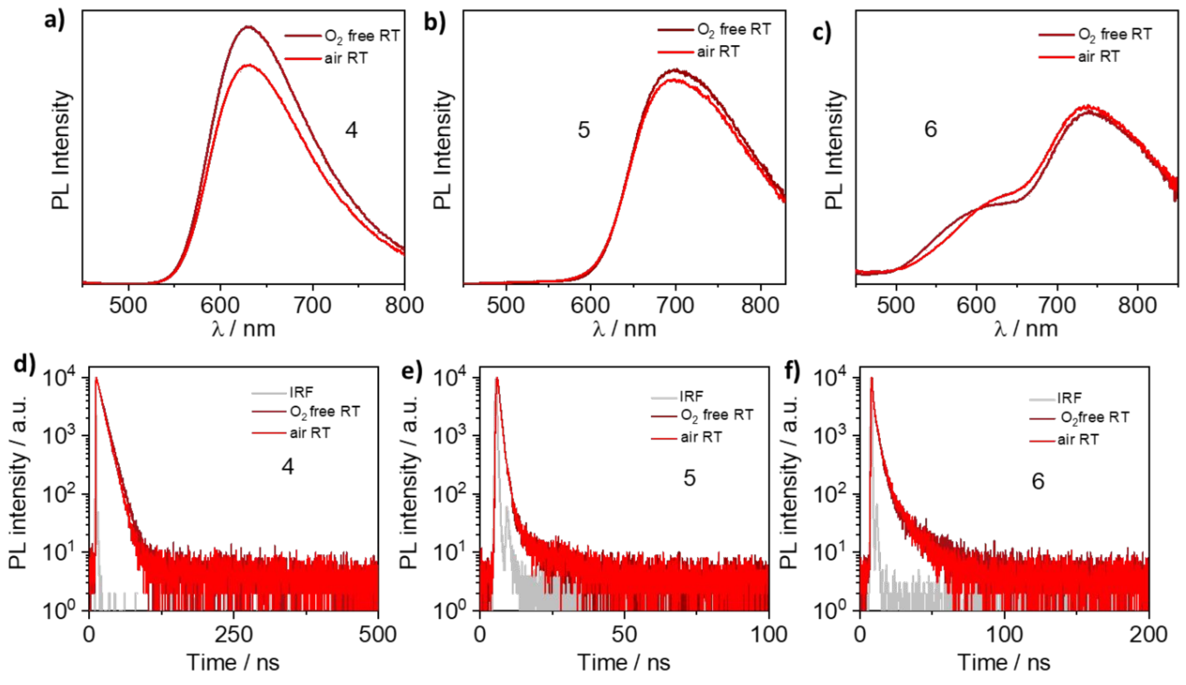

Figure **S63**. Steady-state PL spectra of 10^{-5} M (a) **4** (b) **5** and (c) **6** (λ_{exc} = 330 nm) and time-resolved PL decays of 10^{-5} M (d) **4** (e) **5** and (f) **6** (λ_{exc} = 379 nm) in toluene under aerated and degassed conditions.

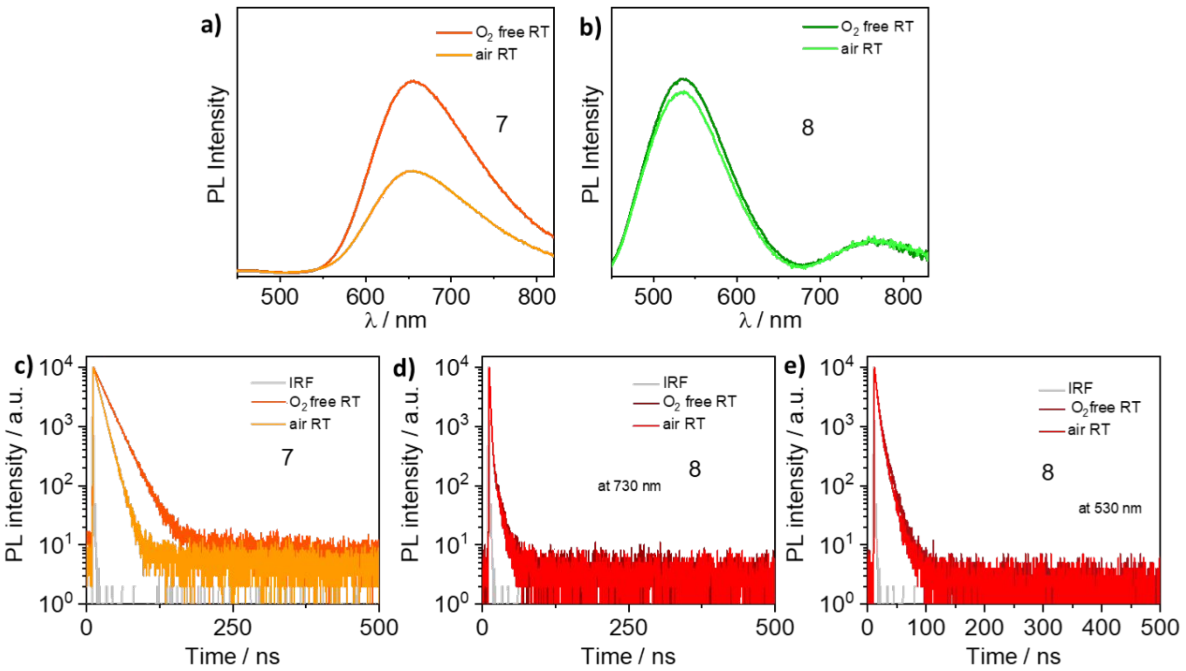

Figure **S64**. Steady-state PL spectra of 10^{-5} M (a) **7** and (b) **8** (λ_{exc} = 330 nm) and time-resolved PL decays of 10^{-5} M (c) **7**, (d) **8** (at λ_{em} = 730 nm) and (e) **8** (at λ_{em} = 530 nm), (λ_{exc} = 379 nm) in toluene under aerated and degassed conditions.

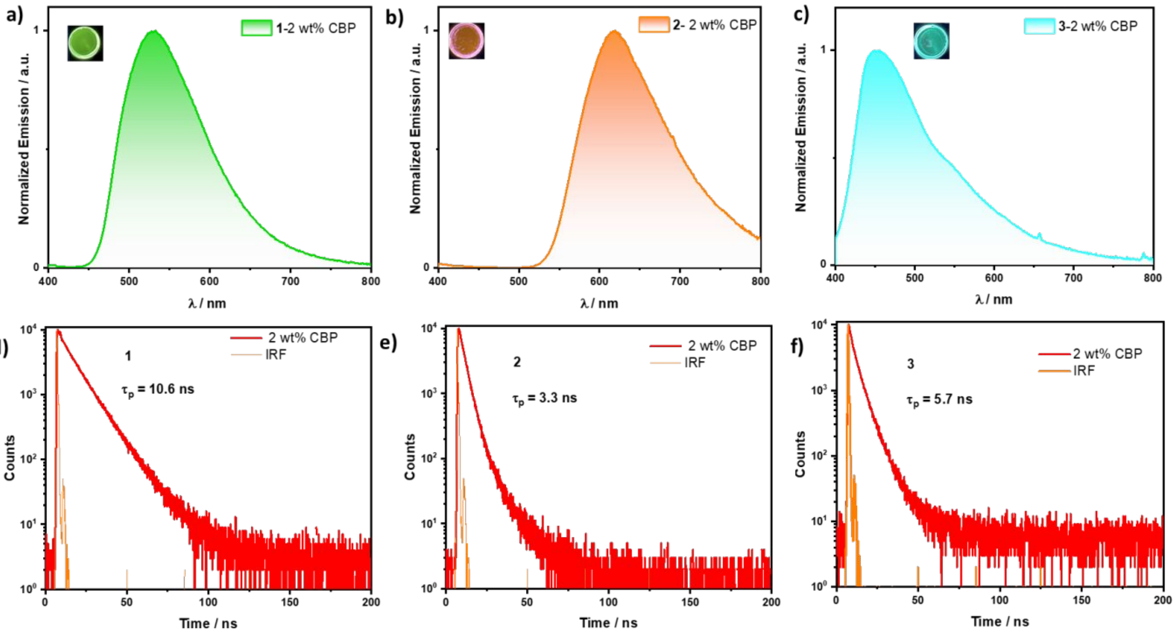

Figure **S65**. Steady-state PL spectra of 5 wt% doped films of a) **1** (b) **2** and (c) **3** in CBP (λ_{exc} = 330 nm) and time-resolved PL decays of 5 wt% films of (d) **1** (e) **2** and (f) **3** in CBP (λ_{exc} = 375 nm) under vacuum.

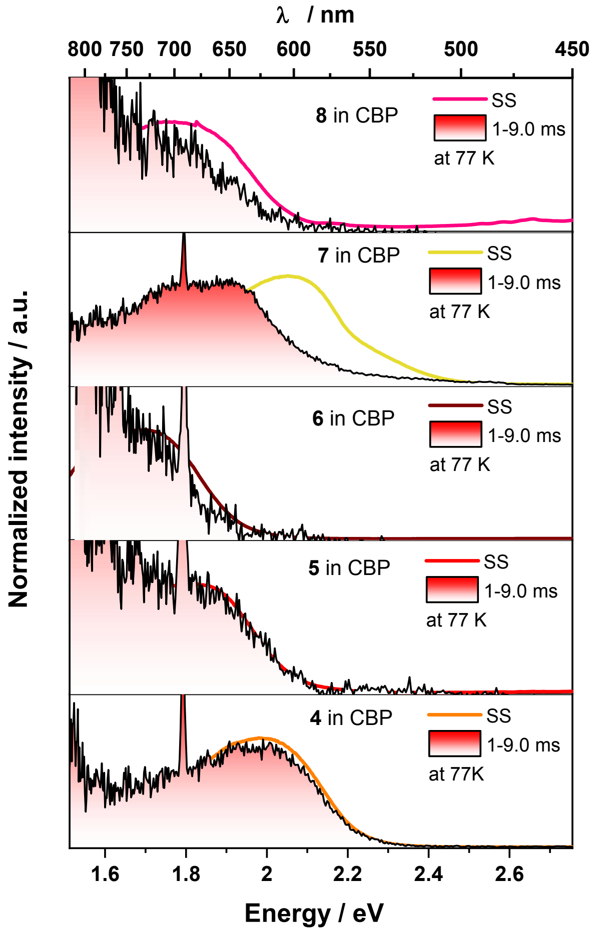

Figure **S66.** 77 K Steady-state PL and phosphorescence spectra of 5 wt% doped films of a) **4**, (b) **5** (c) **6**, (d) **7** and (e) **8** in CBP (λ_{exc} = 340 nm), the ΔE_{ST} value is taken from the onset value difference between the 77 K SSPL and phosphorescence spectra.

Table S1: Optoelectronic properties of compounds **1-3.**

	$E_{1/2}^{red}$ / V[a]	$LUMO^{exp}$ / eV[b]	Optical Gap/eV[c]	λ_{PL}^{Tol} / nm[d]	Φ_{PL}^{Tol} / %[e]	λ_{PL}^{CBP} / nm[f]	Φ_{PL} / %[g]	S_n/T_n / eV[h]	τ / ns[f]
**1**	-1.87	-2.93	2.67	533	42 (37)	530	51.0 (50.8)	2.63/2.08	10.6
**2**	-1.66	-3.14	2.25	612	14 (14)	618	19.5 (19.0)	-/-	3.3
**3**	-1.46	-3.34	2.09	501	5 (5)	495	25.4 (25.2)	2.82/2.43	5.7

a At 298 K in THF with 0.1 M $[nBu_4N]PF_6$ (vs Fc+/Fc). b $E_{LUMO} = -E_{1/2}red - 4.8$ eV. c From the onset of the absorption in toluene. d At 298 K, in degassed toluene at 1 × 10-5 M. e At 298 K, in degassed toluene with values in parentheses under aerated conditions. f In spin-coated 5 wt% doped films in CBP at 298 K. g Spin-coated films of 5 wt% emitters doped in CBP under N_2 with the values in parentheses in air. h Obtained from the onset of the steady-state photoluminescence and phosphorescence spectra (1 ms – 9 ms) at 77 K of 5 wt% films in CBP.

S8. References

[1] M. J. Frisch, G. W. Trucks, H. B. Schlegel, G. E. Scuseria, M. A. Robb, J. R. Cheeseman, G. Scalmani, V. Barone, G. A. Petersson, H. Nakatsuji, X. Li, M. Caricato, A. V. Marenich, J. Bloino, B. G. Janesko, R. Gomperts, B. Mennucci, H. P. Hratchian, J. V. Ortiz, A. F. Izmaylov, J. L. Sonnenberg, Williams, F. Ding, F. Lipparini, F. Egidi, J. Goings, B. Peng, A. Petrone, T. Henderson, D. Ranasinghe, V. G. Zakrzewski, J. Gao, N. Rega, G. Zheng, W. Liang, M. Hada, M. Ehara, K. Toyota, R. Fukuda, J. Hasegawa, M. Ishida, T. Nakajima, Y. Honda, O. Kitao, H. Nakai, T. Vreven, K. Throssell, J. A. Montgomery Jr., J. E. Peralta, F. Ogliaro, M. J. Bearpark, J. J. Heyd, E. N. Brothers, K. N. Kudin, V. N. Staroverov, T. A. Keith, R. Kobayashi, J. Normand, K. Raghavachari, A. P. Rendell, J. C. Burant, S. S. Iyengar, J. Tomasi, M. Cossi, J. M. Millam, M. Klene, C. Adamo, R. Cammi, J. W. Ochterski, R. L. Martin, K. Morokuma, O. Farkas, J. B. Foresman, D. J. Fox, Wallingford, CT **2016**.
[2] C. Adamo, V. Barone, *J. Chem. Phys.* **1999**, *110*, 6158.
[3] S. Grimme, *Chem. Phys. Lett.* **1996**, *259*, 128.
[4] S. Hirata, M. Head-Gordon, *Chem. Phys. Lett.* **1999**, *314*, 291.
[5] Sheldrick, G.M. . Acta Cryst. 2015, A71, 3-8.
[6] Dolomanov, O.V., Bourhis, L.J., Gildea, R.J, Howard, J.A.K. & Puschmann, H. J. Appl. Cryst. 2009, 42, 339-341.
[7] Sheldrick, G.M. . Acta Cryst. 2015, A71, 3-8.
